# Flavonoids: biological activities, nutritional and immunological aspects, therapeutic potential, food applications, and human health benefits—A comprehensive review

**DOI:** 10.3389/fnut.2026.1801674

**Published:** 2026-09-02

**Authors:** Mohamed T. El-Saadony, Ahmed M. Saad, Mohamed A. Fahmy, Dina Mostafa Mohammed, Dina Khodeer, Esmael M. Alyami, Samar Sami Alkafaas, Mthokozisi Dladla, Soumya Ghosh, Mohammad A. A. Al-Najjar, Synan F. AbuQamar, Khaled A. El-Tarabily

**Affiliations:** 1 Department of Agricultural Microbiology, Faculty of Agriculture, Zagazig University, Zagazig, Egypt; 2 Department of Biochemistry, Faculty of Agriculture, Zagazig University, Zagazig, Egypt; 3 Nutrition and Food Sciences Department, National Research Centre, Dokki, Giza, Egypt; 4 Department of Pharmacology, College of Medicine, Imam Mohammad Ibn Saud Islamic University (IMSIU), Riyadh, Saudi Arabia; 5 Department of Biology, College of Science, King Khalid University, Abha, Asir, Saudi Arabia; 6 Molecular Cell Biology Unit, Division of Biochemistry, Department of Chemistry, Faculty of Science, Tanta University, Tanta, Egypt; 7 Human Molecular Biology Unit, School of Biomedical Sciences, Faculty of Health Sciences, University of the Free State, Bloemfontein, South Africa; 8 Natural and Medical Sciences Research Center, University of Nizwa, Nizwa, Oman; 9 Department of Pharmaceutical Sciences and Pharmaceutics, Faculty of Pharmacy, Applied Science Private University, Amman, Jordan; 10 Department of Biology, College of Science, United Arab Emirates University, Al Ain, United Arab Emirates

**Keywords:** bioactive compounds, bioavailability, biosynthesis, clinical therapies, drug delivery systems, mechanisms of action

## Abstract

The global burden of infectious and non-communicable diseases presents a critical challenge, highlighting the urgent need for novel, safe, and effective therapies. Bioactive compounds from medicinal plants, especially flavonoids, offer a promising resource for drug discovery due to their diverse and potent pharmacological effects documented in preclinical studies. This comprehensive review summarizes current knowledge of flavonoids, including their classification into 14 main groups and their production via biosynthesis, chemical synthesis, modification, and extraction. While structural modification remains a key aspect of drug development, biosynthesis is increasingly seen as a potential transformative strategy for sustainable large-scale production. However, it requires a deeper understanding of metabolic pathways and optimization of synthetic methods. Aside from production challenges, a major translational hurdle is the inherently low bioavailability of many flavonoids. This review critically examines innovative solutions under investigation, such as advanced nanoparticles and colloidal drug delivery systems designed to address challenges in solubility, stability, and absorption. It also summarizes the current understanding of the mechanisms of action underlying their broad biological activities, primarily as evidenced by preclinical models, including nutritional, immunological, and disease-specific effects, and discusses potential drug interactions warranting clinical attention. Lastly, the review highlights key research gaps, including the need for more robust *in vivo* validation, standardized extracts to improve reproducibility, and well-designed clinical trials to verify efficacy in humans. Future directions include leveraging metabolic engineering and artificial intelligence to optimize biosynthesis, as well as targeted clinical studies of advanced delivery systems. By integrating these approaches, this review aims to establish a framework for ongoing research on flavonoids to inform future translational efforts that may eventually support their development as clinical therapies.

## 1 Introduction

Flavonoids are a major group of secondary metabolites found in many fruits, vegetables, herbs, cereals, nuts, and various other plant parts, including flowers, stems, and seeds (1). To date, over 10,000 distinct flavonoid structures have been identified and characterized (2, 3). Renowned for their powerful antioxidant and diverse biochemical properties, flavonoids show great potential in preventing and managing serious diseases, including cancer, cardiovascular disorders, and neurodegenerative conditions (2).

These broad health benefits make flavonoids highly attractive for applications in nutraceutical, pharmaceutical, and cosmetic formulations. Their therapeutic potential is primarily attributed to their antioxidative, anti-inflammatory, anti-carcinogenic, and anti-mutagenic activities, as well as their ability to modulate key cellular enzyme functions (4). Biosynthetically derived from the phenylpropanoid pathway, all flavonoids share a characteristic three-ring (C6–C3–C6) skeleton consisting of 15 carbon atoms, arranged into the A, B, and C rings (5). Flavonoids perform essential biological functions across plants, animals, and microorganisms (6).

In plants, flavonoids contribute to pigmentation, scent, and flavor, particularly in flowers, fruits, and leaves (7). As vital secondary metabolites, flavonoids regulate key physiological processes, including auxin transport, fertility, pollination, and seed development (8). Furthermore, flavonoids play a crucial defensive role against abiotic stresses, such as ultraviolet radiation, drought, and heavy metal exposure, as well as biotic threats posed by pathogens and herbivores (9). Based on their chemical structure, particularly the degree of unsaturation and oxidation of the central ring, flavonoids are classified into several groups, including flavones, flavanones, isoflavones, flavonols, chalcones, flavanols, and anthocyanins (10, 11).

Despite the extensive body of research on flavonoid bioactivity, significant knowledge gaps persist, impeding the translation of these compounds from promising preclinical candidates to reliable clinical applications. First, although the biosynthetic pathways of major flavonoid classes are well characterized, the regulatory mechanisms governing tissue-specific accumulation and stress-induced production remain incompletely understood, limiting efforts to engineer high-yield production systems.

Second, while numerous studies have documented the antioxidant and anti-inflammatory effects of flavonoids *in vitro*, the extent to which these mechanisms translate into *in vivo* efficacy is often confounded by the notoriously low oral bioavailability of these compounds, a challenge that current formulation strategies have yet to overcome, despite rigorous clinical validation.

Third, most of the evidence supporting the therapeutic potential of flavonoids in cancer, cardiovascular disease, and neurodegeneration derives from preclinical models, with a striking paucity of well-designed, adequately powered human trials demonstrating clinically meaningful outcomes. Fourth, despite growing interest in flavonoid-based nanoparticles, systematic evaluations of their safety, long-term toxicity, and comparative efficacy with conventional formulations remain lacking. Finally, inconsistencies in the composition and standardization of flavonoid-containing extracts across studies hamper reproducibility and complicate meta-analytic efforts to establish definitive conclusions regarding their health effects.

This current review systematically summarizes recent advances in flavonoid research, encompassing their classification, biosynthetic pathways, diverse biological activities, and expanding applications in the food, cosmetic, and pharmaceutical industries (11–13). We also examined the contribution of omics technologies to the discovery of functional genes and novel bioactive compounds and evaluate recent advances in the development of flavonoid-based nanoparticles for therapeutic applications.

Ultimately, this review provides a contemporary overview of flavonoid research, emphasizing the substantial therapeutic potential of flavonoid nanoparticles in addressing major diseases and improving public health. By explicitly identifying and critically evaluating these knowledge gaps, this review aims to provide a roadmap for future research priorities, prioritizing rigorous *in vivo* validation, standardization of experimental models, and well-designed clinical investigations to bridge the translational divide between flavonoid bioactivity and clinical applicability.

Adopting a systematic approach, this study conducted a comprehensive analysis of plant flavonoids, encompassing their classification, biosynthesis, regulatory mechanisms, biological functions, practical applications, omics-based insights, and nanoparticle synthesis. The search employed both individual and combined keywords to ensure broad coverage and retrieval of relevant studies. Using the PRISMA (Preferred Reporting Items for Systematic Reviews and Meta-Analyses) flow diagram, the literature selection procedure was methodically recorded (14, 15).

## 2 Methodology

This comprehensive review was conducted in accordance with the PRISMA guidelines to ensure a transparent and reproducible evaluation of flavonoid research.

### 2.1 Search strategy and data sources

A systematic literature search was performed across major scientific databases, including PubMed/MEDLINE, Web of Science, Scopus, and Google Scholar. The search strategy employed both individual key terms and Boolean combinations to capture the multidimensional aspects of the field.

**(i) Primary keywords:** “Flavonoids,” “Biosynthesis,” “Drug Delivery Systems,” “Bioactivity,” and “Nano-delivery systems.”

**(ii) Total sources:** This review synthesizes findings from over 470 peer-reviewed sources, encompassing original research articles, systematic reviews, and meta-analyses.

### 2.2 Inclusion and exclusion criteria

The selection process focused on high-impact studies published primarily within the last two decades to ensure the inclusion of recent advancements in omics and nanotechnology.

#### 2.2.1 Inclusion criteria

Studies detailing the classification of the 14 primary flavonoid groups; research on biosynthetic, chemical synthesis, and structural modification pathways; and investigations into biological mechanisms, including antioxidant, anti-inflammatory, and anticancer properties.

#### 2.2.2 Exclusion criteria

Studies from non-peer-reviewed sources or those with insufficient experimental detail were excluded to maintain scientific rigor.

### 2.3 Data synthesis and evidence qualification

Based on the reviewers' feedback, the retrieved literature was categorized by the level of evidence. While the review catalogs the diverse pharmacological potential of flavonoids identified across 10,000 distinct structures, the methodology explicitly distinguishes between:

**(i) Preclinical data:** Most current findings regarding nutritional, immunological, and disease-specific benefits are rooted in *in vitro* and animal models.

**(ii) Clinical evidence:** The limited number of human trials currently available identifies these as a critical “translational gap” that necessitates future evidence-based validation.

## 3 Classification of flavonoids

In terms of classification, flavonoids predominantly accumulate as glycosides within plant cell vacuoles. Their core structure is characterized by a C6–C3–C6 skeleton composed of three rings, designated as A, B, and C (16).

**Figure 1:**
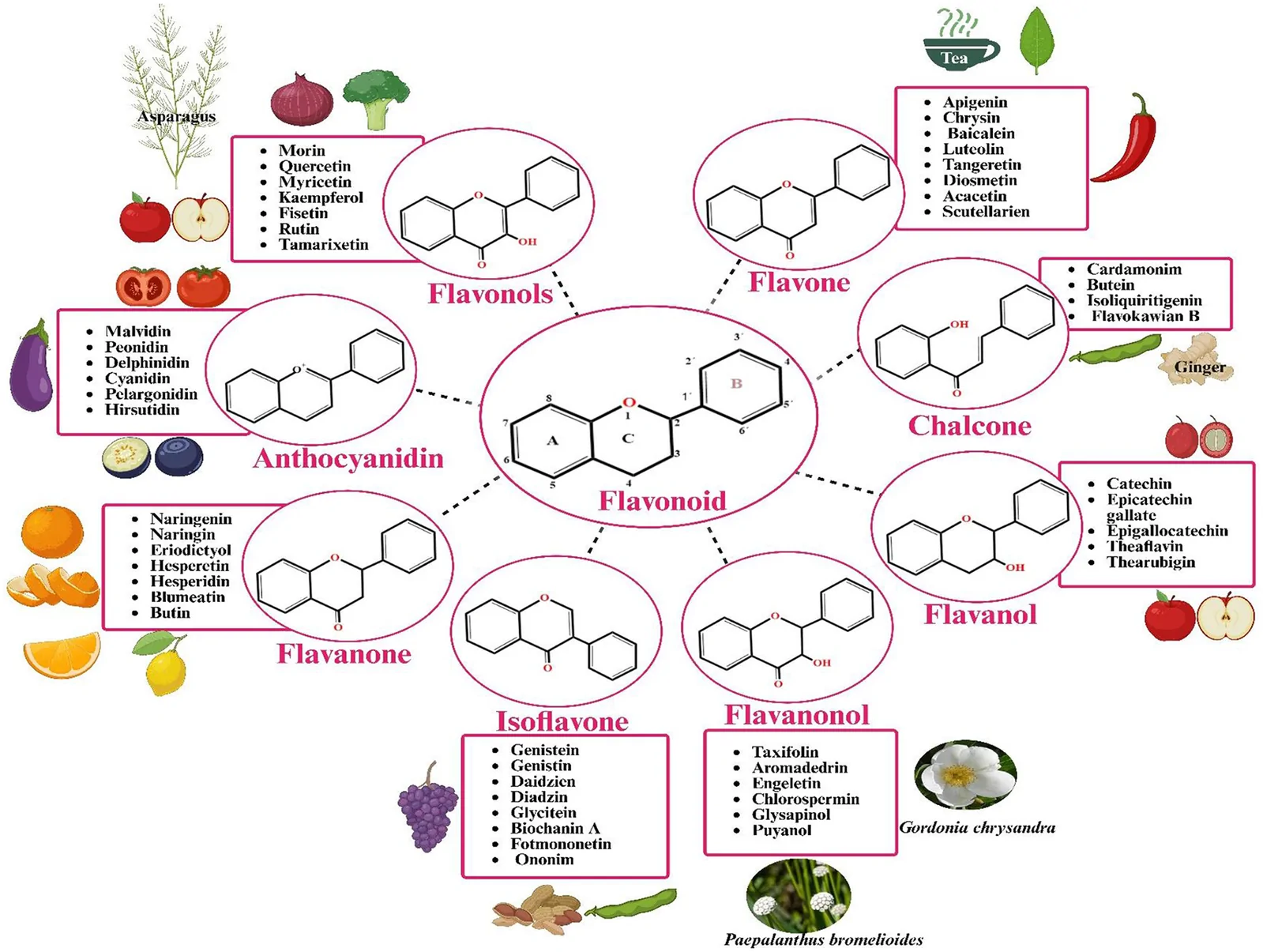
Classification and main food sources of flavonoids.

These compounds are systematically divided into seven major subclasses: flavonols, flavones, isoflavones, anthocyanidins, flavanones, flavanols, and chalcones. The oxidation state of the central heterocyclic C ring primarily determines this classification. Additional structural diversity arises from modifications, such as glycosylation and acylation, occurring at the methyl and hydroxyl groups on the A and B rings. Figure 1 illustrates the classification and main food sources of flavonoids.

### 3.1 Flavanols

Flavanols, a prominent subclass of flavonoids, are distinguished by the presence of a hydroxyl group at the 3-position of the C ring and the absence of a C2–C3 double bond. The A and B rings' various hydroxylation patterns give them their structural variety (16). These compounds serve as monomeric precursors to oligomeric and polymeric proanthocyanidins. In the human diet, flavanols are widely distributed across various fruits, including apples, berries, plums, and apricots, with the skins typically containing the highest concentrations (17).

The primary health benefits of flavanol consumption are attributed to their bioactive metabolites. Following ingestion, phase II and microbiota-derived metabolites circulate in the bloodstream, where they exert physiological effects. A key mechanism of action involves these metabolites enhancing nitric oxide release, thereby improving vascular function (18).

For instance, clinical studies in smokers have shown that consuming flavanol-rich beverages can alleviate certain tobacco-induced vascular impairments by increasing nitric oxide bioavailability. Consequently, regular intake of flavanol-rich foods is associated with long-term improvements in endothelial function and a reduced risk of cardiovascular diseases (19, 20).

### 3.2 Anthocyanidins

Anthocyanidins, the core aglycone structures of anthocyanins, are inherently chemically unstable. Consequently, they predominantly occur in nature in their glycosylated forms, collectively known as anthocyanins (21, 22). These molecules act as natural pigments, imparting the characteristic red, purple, and blue hues observed in many flowers and fruits (23).

Anthocyanins are chemically characterized as glycosides of polyhydroxy and polymethoxy derivatives. Their structural diversity arises from variations in the number and positions of hydroxyl and methoxyl groups attached to the flavylium cation backbone. To date, more than 650 distinct anthocyanins have been identified, with the most common representatives including delphinidin, cyanidin, petunidin, peonidin, malvidin, and pelargonidin (24).

These pigments are abundant in a wide range of fruits and vegetables, including blueberries, red cabbage, and purple sweet potatoes. One of the principal biological functions of anthocyanins is their potent antioxidant activity, which may act through both exogenous and intrinsic mechanisms. Their efficiency in scavenging reactive oxygen and nitrogen species is strongly dependent on structural characteristics. Key determinants of this activity include the orientation of the aromatic rings, the number and position of free hydroxyl groups on the pyrone ring, and the degree of ortho-hydroxylation and methoxylation on the B ring, the latter being particularly associated with enhanced antioxidant potency (25).

Although glycosylation typically diminishes antioxidant capacity, rendering anthocyanidins more active than their glycosylated counterparts (26), acylation can substantially enhance this activity. Beyond their antioxidant effects, anthocyanins confer numerous health benefits in humans, including supporting cholesterol metabolism, enhancing visual acuity, and reducing the risk of cardiovascular disease (27, 28).

Anthocyanins are also extensively used as natural colorants in the food industry. For example, carrots serve as a source of mono-acylated anthocyanins used for coloring, while the anthocyanins extracted from butterfly pea (*Clitoria ternatea*) provide a remarkably stable blue pigment (29, 30).

### 3.3 Flavanones

Flavanones, a subclass of dihydroflavones, are distinguished from other flavonoids by their saturated C ring, which does not have two carbon atoms linked together in a double bond. The Rutaceae family of citrus fruits is where citrus fruits are most often found, including oranges and lemons (31).

A notable feature of citrus flavanones is their propensity to form conjugates with various glycosides, resulting in a wide diversity of structurally distinct compounds. Characteristic flavanones such as naringin and pomelo rutin contribute to the distinctive bitterness of citrus juice and peel. Common examples include hesperidin and naringin in oranges, eriodictyol in lemons, and naringenin in grapefruit, with their concentrations varying considerably among different citrus cultivars (32).

Red citrus varieties, for instance, contain particularly high concentrations of both flavanones and anthocyanins. Functionally, flavanones act as potent antioxidants by donating hydrogen atoms from their phenolic groups. For example, naringin has been shown to enhance the activity of key antioxidant enzymes, including catalase (CAT), superoxide dismutase (SOD), glutathione peroxidase (GPx), and paraoxonase (PON) (33, 34). This enhanced enzymatic activity significantly strengthens the immune system and protects organs and tissues from oxidative damage. Experimental studies have further shown that naringenin and hesperetin can ameliorate age-related impairments in thyroid function in rats (35).

Furthermore, flavanones do not adversely affect liver histology and have been shown to reduce serum levels of hepatic enzymes, including alanine aminotransferase (ALT) and aspartate aminotransferase (AST) (36). The health-promoting effects of naringenin are partly attributed to its antioxidant capacity and ability to neutralize free radicals. In addition, naringenin effectively suppresses lipopolysaccharide-induced secretion of proinflammatory cytokines in macrophages and reduces nitrate and nitrite levels, actions associated with the attenuation of intestinal edema during inflammatory responses (37).

### 3.4 Flavonols

Flavonols, a subclass of flavonoids derived from the 3-hydroxyflavone backbone, are characterized by specific substitution patterns on the A and B rings connected through a three-carbon bridge (38). The A ring is typically hydroxylated at the 5- and 7-positions, while the presence of a hydroxyl group at the 3-position serves as the key structural feature distinguishing flavonols from other flavonoid subclasses. These compounds are predominantly localized in plant epidermal cells, where they act as protective agents against harmful solar radiation, particularly ultraviolet (UV) light, thereby safeguarding cellular DNA from photodamage (39).

Common flavonols include kaempferol, quercetin, myricetin, and galangin, which are abundantly present in a wide range of fruits and vegetables, such as onions, apples, broccoli, and asparagus (40). As bioavailable dietary constituents, flavonols confer a wide spectrum of health benefits, exhibiting antioxidant, cardioprotective, antibacterial, antiviral, and anticancer activities (41). Studies conducted on porcine and human hepatocytes have shown that myricetin enhances hepatic function by modulating the mitogen-activated protein kinase (MAPK) signaling pathway (42).

Quercetin counteracts reactive oxygen species (ROS)-mediated hepatocarcinogenesis by enhancing both enzymatic antioxidant defenses, such as SOD, GPx, and CAT, and non-enzymatic defenses like reduced glutathione (GSH). It inhibits ROS production by modulating signaling pathways, including Rac1-p66Shc, reduces cancer cell migration and proliferation, and induces apoptosis by regulating pro- and anti-apoptotic proteins. These mechanisms collectively contribute to its protective role against liver cancer development (43).

Furthermore, evidence from population-based studies has shown that, specifically in women and smokers, a high dietary intake of flavonols is substantially linked to a lower risk of gastric cancer (44).

### 3.5 Isoflavones

Isoflavones, based on the 3-phenylchromen-4-one backbone, constitute a class of compounds predominantly found in leguminous plants (45, 46). Soy and its derivatives represent the primary dietary sources of isoflavones, with alfalfa and chickpeas serving as secondary sources. Minor amounts are also found in various fruits, vegetables, nuts, and cereals. Soybeans contain twelve distinct isoflavone isomers, among which genistein and daidzein are the most prominent and act as potent natural antioxidants (47, 48).

Due to their structural similarity to estradiol-17β, isoflavones are classified as phytoestrogens (49). Certain phytoestrogens can bind to estrogen receptors, exhibiting both estrogenic and anti-estrogenic properties. Since immune cells, including thymocytes, lymphocytes, and macrophages, express estrogen receptors, these interactions can modulate immune function by suppressing excessive activation and attenuating hypersensitivity reactions. Moreover, isoflavones act as potent antioxidants; by mitigating free radical-induced DNA damage, they contribute to reducing long-term cancer risk, a property particularly notable for genistein and daidzein (50).

### 3.6 Flavones

Flavones, a major subclass of flavonoids, are characterized by a 4H-chromen-4-one core structure with a phenyl substituent at the C2 position (16). They are common constituents of the human diet and are abundant in foods such as celery, tea, red peppers, and oranges. Flavones frequently occur in nature as 7-O-glycosides, with apigenin and luteolin being the predominant dietary representatives. Apigenin typically exists in glycosylated forms, in which its aglycone is conjugated to a sugar moiety through O- or C-glycosidic linkages, forming compounds such as apiin, vitexin, and schaftoside (51).

Functionally, apigenin serves as a potent free radical scavenger and enhances the antioxidant defense mechanisms within pancreatic cells (52). It also exhibits pronounced anti-inflammatory activity; pretreatment with apigenin has been shown to attenuate inflammation associated with cancer, cardiovascular disorders, and neuroinflammatory conditions (53). These beneficial effects are mediated through the modulation of gene transcription, protein expression, and enzymatic activity, thereby preventing the depletion of antioxidant enzymes in cells exposed to streptozotocin (54).

### 3.7 Chalcones

Chalcones (1, 3-diaryl-2-propen-1-ones) are open-chain flavonoids characterized by the attachment of up to three C5-, C10-, or C15-prenyl groups to their aromatic rings (16, 55). Numerous plant groups, such as the Fabaceae, Moraceae, Zingiberaceae, and Cannabaceae, contain chalcones in large quantities. These substances have antiviral, antibacterial, anticancer, and antioxidant properties, among other pharmacological actions (56).

As essential biosynthetic precursors of flavonoids and isoflavonoids, chalcones possess a relatively simple chemical structure that allows for facile synthesis from basic aromatic precursors. This structural simplicity, coupled with their notable biological activity, has stimulated extensive research focused on the design of synthetic analogs and structural modification of natural chalcones, leading to the development of numerous potent derivatives (57). Notable examples such as xanthohumol and isobavachalcone exhibit particularly strong biological and pharmacological profiles (58).

## 4 Sources of bioactive compounds of flavonoids

Naturally occurring flavonoids represent a major class of polyphenolic compounds that function as essential phytochemicals, signaling molecules, and metabolic intermediates in plants that regulate key biological processes, including growth, development, and ripening (59).

They are especially abundant in angiosperms, with certain botanical families, such as Lamiaceae, Acanthaceae, and Asteraceae, accumulating particularly high levels. The distribution of specific flavonoid subclasses often carries taxonomic significance. For instance, flavonols are nearly ubiquitous in dicotyledons, dihydroflavonoids are especially prevalent in Rosaceae and Rutaceae, and isoflavonoids are characteristic of Leguminosae and Iridaceae (31).

**Figure 2:**
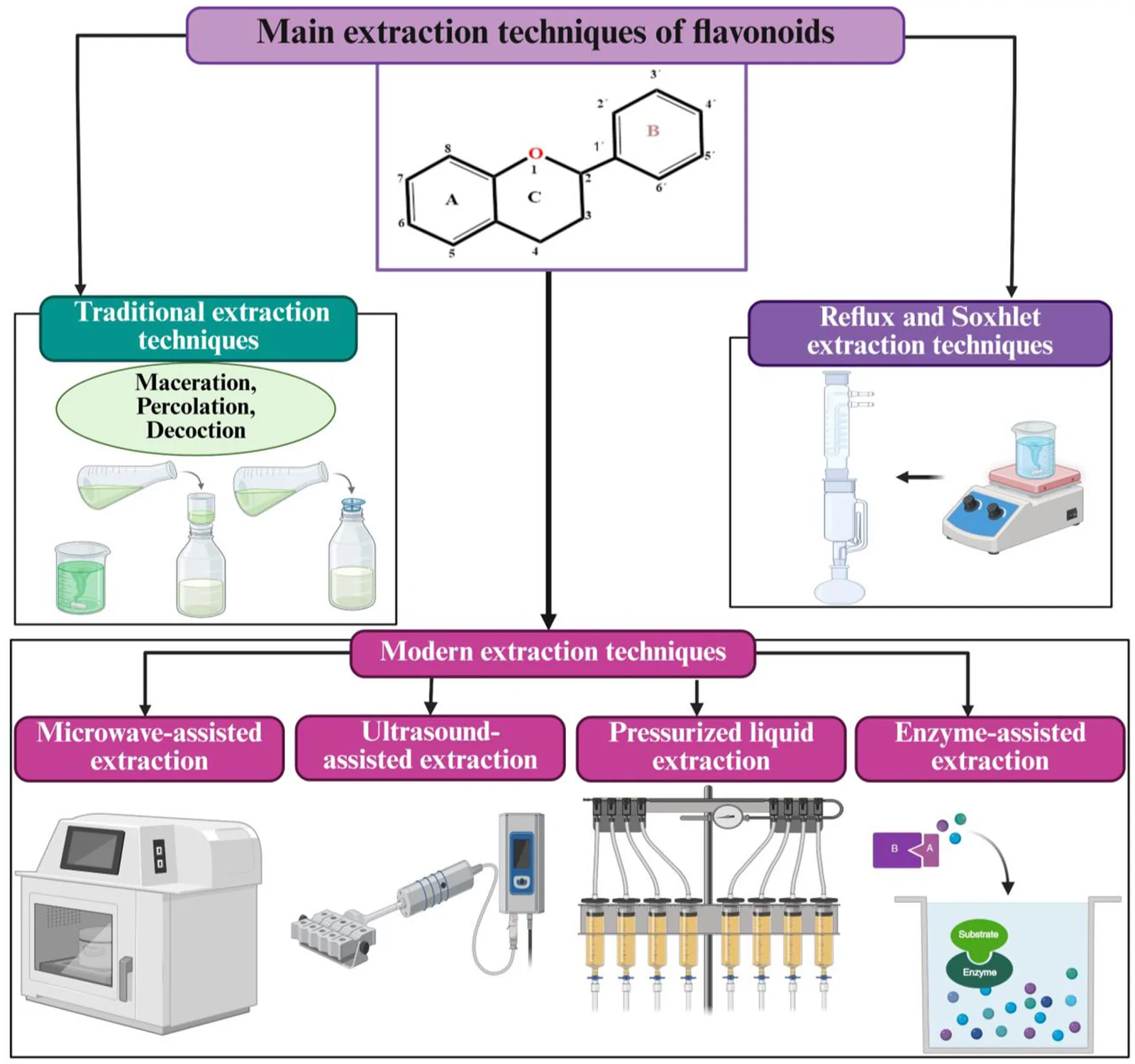
Main extraction techniques of flavonoids.

Although less widespread, flavonoids are also found in some gymnosperms, including bioflavonoids present in *Coniferopsida* and *Ginkgopsida*. Moreover, the edible portions of many common fruits and vegetables, such as *Capsicum annuum, Allium cepa, Citrus sinensis*, and *Vitis vinifera*, serve as important dietary sources of these bioactive compounds. Traditionally, solvent-based processes, including maceration, Soxhlet extraction, and ultrasonic-assisted extraction, are used to extract flavonoids from plant sources (60), and ultrasonic extraction (61) remains widely employed. These conventional techniques are often complemented by advanced methodologies, including microwave-assisted extraction (MAE), supercritical fluid extraction, and various chromatographic techniques (Figure 2). Recent research efforts have focused on optimizing these processes for higher efficiency and yield. For instance, Biswas et al. (62) employed response surface methodology (RSM) to optimize the parameters for ultrasound-assisted extraction (UAE) of total flavonoids from *Corchorus olitorius* leaves.

Subsequent analyses using high-performance liquid chromatography (HPLC) and liquid chromatography–tandem mass spectrometry (LC–MS) confirmed the presence of isoquercetin and hypericin, thereby validating the effectiveness of the optimized UAE protocol (62). In a related study on *Tamarindus indica*, a traditional medicinal plant rich in bioactive compounds, Cui and colleagues (63) employed the UAE method using a deep eutectic solvent (DES). By optimizing critical parameters, including a 20% water content in the solvent, an ultrasonic power of 300 W, an extraction time of 40 min, and a solid-to-liquid ratio of 60 mg L^−1^, the researchers achieved a maximum flavonoid yield of 26.54 mg g^−1^ (63).

Several advanced extraction methods have demonstrated superior flavonoid yields compared to conventional techniques. MAE, UAE, pressurized liquids, supercritical fluids, and DES-based systems are generally more environmentally friendly, with documented reductions in solvent use and energy per unit of flavonoid extracted compared with maceration/Soxhlet (64).

DES–UAE/MAE systems are specifically highlighted as green, recyclable, and suitable for large-scale industrialization, with demonstrated DES recyclability and resin-based product recovery (65). However, MAE is still described as being in an early stage of industrialization, with equipment design, continuous operation, and scale-up of energy delivery and safety as major unresolved issues (66).

Similarly, while microbial cell factories promise higher titers and a more controllable, land-sparing route than plant extraction or chemical synthesis, most strains remain below industrial benchmarks and are not yet commercialized due to metabolic bottlenecks and process complexity (67).

In *Acanthopanax senticosus*, the DES–UAE method achieved total flavonoids of 23.93 mg g^−1^, representing a 40.7% increase over ultrasonic ethanol extraction under identical conditions, with the DES reusable for at least two cycles (68). Similarly, the DES–MAE method on jujube yielded the highest total flavonoids at 8.03 mg g^−1^, significantly outperforming water- or ethanol-bath methods (69).

For *Fructus aurantii*, DES–UAE produced naringin and neosperidin concentrations of 48.18 mg g^−1^ and 34.50 mg g^−1^, respectively, markedly higher than those of extraction with 95% ethanol, methanol, or water controls (65). In sea buckthorn leaves, both DES–MAE and DES–UAE achieved total flavonoid yields of 20.82 mg g^−1^, representing a 1.3- to 2.4-fold increase over traditional extraction methods (70).

Among the methods applied to *Lactuca indica*, UAE yielded the highest flavonoid content at 48.01 mg g^−1^, outperforming both conventional solvent extraction and MAE (71). In citrus waste, a simple UAE bath with ethanol–water achieved the highest total flavonoid and key flavanone yields compared to MAE and maceration (72). Finally, across *Sidertius* species, assisted extraction methods, including MAE, UAE, and high-pressure extraction, achieved overall metabolite recovery approximately three times higher than conventional extraction (73). Figure 2 delineates the main extraction procedures for flavonoids.

### 4.1 Role of artificial intelligence in improving extraction efficiency

Artificial intelligence (AI) is primarily used to model, predict, and optimize extraction conditions to achieve higher yields or better antioxidant activity with fewer experiments.

AI models (ANN (artificial neural networks), ANFIS (adaptive neuro-fuzzy inference systems), random forest, gradient boosting, and eXtreme gradient boosting) capture complex non-linear relationships between factors (solvent, time, temperature, power, solid–liquid ratio) and responses (yield, total phenolic content (TPC), total flavonoid content (TFC), and antioxidant activity) more effectively than response surface methodology. This gives higher R^2^ and lower error, and thus more reliable optimal conditions (74, 75).

In multiple studies, ANN-based or hybrid models [response surface methodology (RSM)-ANN-genetic algorithms (GA), ANN-GA, ANN-particle swarm optimization (PSO), PSO-GA-backpropagation neural network (BPNN)] consistently outperformed RSM in predicting flavonoid yield and purification performance (76, 77).

AI-optimized ultrasonic/DES systems achieved significantly higher total flavonoid yields than conventional or RSM-only optimization. For example, 167.56 mg g^−1^ in sweet tea was predicted and achieved using natural deep eutectic solvents (NADES)- UAE via ANN (78).

Furthermore, higher yields for *Juglans mandshurica* flavonoids using RSM-ANN-GA UAE vs. traditional solvent extraction (75), and higher yields of six Astragali radix (*Astragalus propinquus*) flavonoids with PSO-GA-BPNN vs. RSM (77). Whilst similar advantages in creeping woodsorrel (*Oxalis corniculate*), barrenwort (*Epimedium* sp.), Baikal skullcap (*Scutellaria* sp.), and others (79). AI reduces the number of experimental runs, reagent use, and time needed to find optimal conditions (80).

ANNs and genetic algorithms, or other metaheuristics, allow simultaneous optimization of multiple responses (total phenols, flavonoids, flavonols, tannins) under ultrasound, rather than one output at a time (79). Models can optimize both extraction and downstream purification steps, improving adsorption/desorption capacities on macroporous resins beyond RSM-derived settings (77).

Machine learning can also directly optimize bioactivity endpoints (antioxidant activity), not only yields, and even predict antioxidant activity from process variables and flavonoid content using XGBoost or radio frequency (RF), enabling extraction design (81). Tree-based models and feature-importance analyses [RF, gradient boosted regression trees (GBRT), shapley additive explanations (SHAP)] rank the variables that most affect extraction efficiency (often: solid–liquid ratio > power > time > solvent concentration) (82). The sources of bioactive flavonoid compounds are delineated below.

### 4.2 Rice (*Oryza sativa*)

As a staple cereal crop, rice serves as a major global energy source and provides essential minerals along with diverse bioactive compounds (83). Among these, phenolics represent the primary class of bioactives, predominantly concentrated in the bran and germ. The principal flavonoids identified in rice include tricin-O-glycosides, flavone O-glycosides, flavonol-O-glycosides, and their acylated derivatives (84).

Colored rice varieties generally exhibit higher phenolic and flavonoid contents than white rice, as grain pigmentation is closely correlated with flavonoid accumulation, a characteristic also observed in related species such as *Zizania latifolia* and *Zizania palustris* (85).

### 4.3 Soybean (*Glycine max*)

Soy consumption has been associated with numerous health benefits, including the regulation of blood pressure, maintenance of thyroid and kidney function, and reduction of prostate cancer risk (86).

Soybeans are particularly rich in isoflavones, all of which contain compounds such as daidzein, genistein, daidzin, glycitein, genistin, glycitin, malonyldaidzin, and malonylgenistin (87). In processed soy products, the predominant flavonoids are genistein, daidzein, and glycitein, which typically occur in their glucosidic forms, genistin, daidzin, and glycitin, respectively (88).

### 4.4 Wheat (*Triticum aestivum*)

Among the major cereal crops, wheat provides approximately one-fifth of the global caloric intake and serves as a significant source of bioactive compounds, particularly phenolic acids and flavonoids (89). Conventional amber wheat varieties contain relatively low levels of anthocyanins, pigmented types, such as blue, purple, black, and red wheat, are rich in these compounds, as well as in carotenoids, which are primarily localized in the outer aleurone layer (90).

The distribution of these bioactive compounds within the kernel is heterogeneous. The bran contains the highest concentrations, followed by the germ and, lastly, the endosperm (91).

### 4.5 Corn (*Zea mays*)

Corn, another major food crop, is categorized as either field maize or sweet maize and can further be classified into pigmented and non-pigmented varieties (92). The characteristic red and purple pigmentation observed in certain maize types arises from flavonoids, primarily glucosides of cyanidin, pelargonidin, and peonidin (92).

Corn silk is also a rich source of flavonoids, including luteolin, apigenin, and formononetin. During grain development, the levels of anthocyanins, geranidin, and pelargonidin rose (93).

### 4.6 Citrus (*Citrus* species)

Nutritionally significant citrus fruits, such as oranges, lemons, and grapefruits, contain flavonoids that influence the fruit's color, flavor, and the plant's capacity to tolerate stress (94). In citrus species, these compounds generally occur in glycosylated forms. For instance, after consumption, naringenin is converted into its glycoside, naringin (95).

The dominant flavonoid varies among citrus species. For example, pomelo is abundant in naringin, lemon primarily contains eriocitrin, and orange is rich in hesperidin. In the peels of sweet oranges and kumquats, flavonoids are predominantly stored as polymethoxyflavones (96).

Certain flavanones, such as naringin and neohesperidin, found in grapefruit and pomelo, impart a bitter taste. In contrast, non-bitter varieties like sweet oranges and tangerines predominantly accumulate the odorless compound flavanone-7-O-rutinoside (97).

### 4.7 Apple (*Malus domestica*)

Among fruits consumed worldwide, apples are the most common. A defining characteristic is their peel color, which ranges from yellow and green to the more prevalent red. This red pigmentation arises from anthocyanins, with the precise hue and intensity determined by their composition and concentration (98).

According to Espley et al. (99), anthocyanins also influence the color of the fruit's flesh and its tendency to brown after slicing (99). The accumulation of these pigments is a tightly regulated process. The key proteins, including the transcription factors MdMYB1 (*Malus domestica* MYB1) and MdTCP46 (*Malus domestica* TEOSINTE BRANCHED1/CYCLOIDEA/PCF46), have been shown to promote anthocyanin biosynthesis under high-light stress (100).

Apples are additionally rich in various polyphenolic compounds, though their concentrations vary markedly depending on the cultivar, cultivation conditions, and even the fruit's position on the tree (101).

### 4.8 Grape (*Vitis vinifera*)

Similarly, the well-documented health benefits of grapes and their derivatives are largely attributed to their phenolic constituents, especially flavonoids, including flavonols, proanthocyanidins, and anthocyanins.

The dark coloration of grape skins arises directly from anthocyanin accumulation, making skin color a reliable indicator of anthocyanin content (102). Moreover, the mouthfeel and taste of wine are influenced by the concentration and structural complexity of grape-derived proanthocyanidins (103).

### 4.9 Tomato (*Lycopersicon esculentum*)

In tomatoes, flavonoid accumulation occurs predominantly during the final stages of ripening, as exemplified by anthocyanin-rich purple varieties (104). The ripening process involves the degradation of chlorophyll, accompanied by the simultaneous synthesis of carotenoids and flavonoids (105).

### 4.10 Ginkgo (*Ginkgo biloba*)

Beyond commonly consumed fruits, extracts from *G. biloba* leaves are widely used in health and functional food products due to their high levels of bioactive compounds (106). The principal active constituents are flavonoids, specifically flavonols, flavanols, and anthocyanins (107).

The distribution of flavonoids within the ginkgo tree is uneven, with the highest concentrations found in the leaves, moderate levels in the seed coats, and the lowest in the endosperm. The exceptional longevity of *G. biloba* is partly attributed to flavonoid glycosides and terpenes within its wood (xylem) (108).

Furthermore, flavonoids enhance the tree's tolerance to saline soils (109), while exposure to UV-B radiation has been shown to increase the total flavonoid glycoside content in its leaves (110).

### 4.11 Baikal skullcap (*Scutellaria baicalensis*)

According to the pharmacopoeial standards of both China and Europe, the dried root of *S. baicalensis* is the officially recognized source of the Huang-Qin extract (111). Its principal bioactive constituents are flavonoids, particularly baicalin, baicalein, wogonin, chrysin, and scutellarein (111).

The aerial parts of the plant are also a rich source of flavonoids, including scutellarin, apigenin, baicalin, and baicalein, along with their corresponding glucuronide derivatives. Moreover, extracts obtained from the flowers have been shown to contain up to 19 distinct flavonoid compounds (112).

### 4.12 Tartary buckwheat (*Fagopyrum tataricum*)

Tartary buckwheat (*F. tataricum*), an edible crop belonging to the Polygonaceae family, constitutes another major source of flavonoids, with as many as 147 distinct flavonoid compounds identified across its various tissues (113, 114).

The predominant flavonoid in *F. tataricum* is rutin (quercetin 3-O-rutinoside), which accounts for approximately 0.8–1.7% of the plant's dry weight. Malt derived from *F. tataricum* also contains several flavonoids, including orientin, isoorientin, vitexin, isovitexin, quercetin-3-O-robinobioside, and rutin (115). The distinctive red hue of this malt is attributed to the presence of the anthocyanin cyanidin 3-O-rutinoside. In contrast, common buckwheat (*Fagopyrum esculentum*) is characterized by a high flavonoid concentration in its leaves (116).

Table 1 illustrates the principal dietary sources of flavonoids, including their subclasses and quantified concentrations.

**Table 1:** Major dietary sources of flavonoids: subclasses and quantified concentrations.

Food source	Major flavonoid subclasses	Flavonoid compounds	Quantified concentration	References
Rice *(Oryza sativa)*	Flavones, flavonols.	Tricin-O-glycosides, flavone O-glycosides, flavonol-O-glycosides.	Higher concentrations in pigmented rice than in white rice; specific quantitative values vary by cultivar.	(83, 84)
Soy (*Glycine max*)	Isoflavones.	Daidzein, genistein, glycitein, daidzin, genistin, glycitin, malonyldaidzin, malonylgenistin.	Concentrations vary by processing; glucosidic forms (genistin, daidzin, glycitin) are predominant in processed products.	(86, 87)
Wheat (*Triticum aestivum*)	Anthocyanins, flavonoids.	Anthocyanins, carotenoids (pigmented varieties).	Higher total flavonoids in pigmented (blue, purple, black, red) vs. non-pigmented wheat; bran > germ > endosperm.	(90, 91)
Corn (*Zea mays*)	Anthocyanins, flavones.	Cyanidin glucosides, pelargonidin glucosides, peonidin glucosides, luteolin, apigenin, formononetin.	Anthocyanins increase during grain development; corn silk contains diverse flavonoids.	(92, 93)
Citrus (*Citrus* species)	Flavanones, flavones, polymethoxyflavones.	Naringin (pomelo), eriocitrin (lemon), hesperidin (orange), polymethoxyflavones (peels).	Concentration varies by species; naringin and neohesperidin impart bitterness; flavanone-7-O-rutinoside in non-bitter varieties.	(94–97)
Apple (*Malus domestica*)	Anthocyanins, flavonols, polyphenols.	Anthocyanins (peel color), MdMYB1- and MdTCP46-regulated pigments.	Concentrations vary markedly by cultivar, cultivation conditions, and canopy position.	(98, 99)
Grape (*Vitis vinifera*)	Anthocyanins, flavonols, proanthocyanidins.	Anthocyanins (skin), flavonols (UV protection), proanthocyanidins (mouthfeel).	Anthocyanin content correlates with skin color; flavonols enhance antioxidant capacity.	(102, 103)
Tomato (*Lycopersicon esculentum*)	Anthocyanins, flavonoids.	Anthocyanins (purple varieties), ripening-associated flavonoids.	Accumulation peaks during late ripening; anthocyanin-enriched cultivars exhibit extended shelf life.	(104, 105)
Ginkgo (*Ginkgo biloba*)	Flavonols, flavanols, anthocyanins.	Quercetin, kaempferol, isorhamnetin (glycosides).	Flavonol glycosides comprise up to 24% of the standardized extract; the highest concentrations are in leaves.	(106–110)
Baikal skullcap (*Scutellaria baicalensis*)	Flavones.	Baicalin, baicalein, wogonin, chrysin, scutellarein.	Root is the primary source; aerial parts and flowers contain up to 19 distinct flavonoid compounds	(112)
Tartary buckwheat (*Fagopyrum tataricum*)	Flavonols, anthocyanins, flavones.	Rutin (predominant), orientin, isoorientin, vitexin, isovitexin, cyanidin 3-O-rutinoside.	Rutin accounts for 0.8–1.7% dry weight; 147 distinct flavonoids identified across tissues.	(114)

## 5 Chemical synthesis and structural modification of flavonoids

As public attention to health and the notion of the “dual use of food and medicine” grows, flavonoids are increasingly used as beneficial components in food (117). The demand for efficient flavonoid synthesis has grown as natural extraction cannot meet industrial and biomedical needs. Flavanones such as eriodictyol, pinocembrin, and naringenin serve as versatile scaffolds for generating diverse bioactive derivatives (118).

Traditional chemical synthesis of flavonoids is constrained by multi-step sequences and low yields, whereas photocatalysis and the Algar-Flynn-Oyamada (AFO) reaction have emerged as the primary viable methods. Photocatalysis offers high efficiency and environmental sustainability for industrial applications, while the AFO method generates flavonols via condensation of benzaldehyde derivatives and acetophenone to form 2′-hydroxychalcone, followed by oxidative cyclization under alkaline conditions (119).

Three main methods for chemically altering flavonoids involve the addition of active functional groups, halogen substitution, and metal-ion chelation. Such structural alterations directly affect the biological activities and physicochemical characteristics of flavonoids (120). Flavonoids like rutin, quercetin, and baicalin possess a significant capacity to chelate metal ions such as copper, zinc, and iron, forming complexes that exhibit enhanced bioactivity through synergistic interactions. Furthermore, the strategic introduction of halogen atoms, following the efficiency order of fluorine > chlorine > bromine, represents a potent method for further augmenting the biological activity and therapeutic potential of these natural scaffolds (120).

Furthermore, the incorporation of active groups such as methyl, acyl, and hydroxyl moieties can modify the molecular framework of flavonoids. For example, Copmans et al. (121) screened several flavonoids, including naringenin and kaempferol, for anti-seizure activity using a zebrafish model. Their findings indicated that the methylated analog dimethylated naringenin (NRG-dm) exhibited the highest potency (121).

Increased hydroxylation enhances antioxidant activity and flavonoid solubility in water, but the specific position of hydroxyl groups is crucial for determining bioefficacy. The contribution of hydroxylation is site-dependent, with the most favorable position being C7, followed by C4′ and C3, while hydroxylation at C5 exerts a comparatively minor effect (122).

From a synthetic chemistry perspective, introducing modifications directly onto the C-ring of flavonoids remains particularly challenging, despite the profound impact this region has on the molecule's overall physicochemical and biological properties. Consequently, recent studies have shifted their focus toward functionalizing the more synthetically accessible A and B rings (121).

Illustrating this approach, Wang et al. (123) employed a Mannich reaction to introduce an aminomethylene group at the 8-position of lignan scaffolds, yielding compound 4, a potent cyclin-dependent kinase 1/cyclin B (CDK1/cyclin B) inhibitor. Among the resulting derivatives, 8-N-methylpiperazinylmethylidene xylophilus exhibited particularly strong inhibitory activity, with their half maximal inhibitory concentration (IC_50_) value of 0.92 μmol/L^−1^ (123).

Similarly, flavopiridol and P276-00, two flavonoid-derived cyclin-dependent kinase (CDK) inhibitors whose chemical backbones are derived from the natural compound rohitukine, have progressed to clinical trials for cancer therapy (124, 125). In a complementary investigation, the effectiveness of six naringenin substitutes, each modified at the 7- or 4′-position, in preventing the growth of HCT116 colon cancer cells and in inhibiting cyclin-dependent kinase 2/cyclin E (CDK2/cyclin E) activity was assessed (126).

All six derivatives demonstrated markedly greater potency than the unmodified naringenin parent compound. IC_50_ values for suppressing HCT116 cell growth (1.20 to 20.01 μmol L^−1^) represent a significant improvement over naringenin's IC_50_ of 36.75 μmol L^−1^ (126). Synthesized naringenin derivatives demonstrated higher potency than the parent compound, inhibiting CDK2/cyclin E activity by 42.0%-84.0% at 10 μmol L^−1^, compared to 14.9% for unmodified naringenin (126). Molecular docking revealed that these derivatives form more stable interactions within the enzyme's active site. These findings indicate that the introduction of bulky substituents at the C7 position or nitrogen-containing functional groups at the C4′ position markedly enhances anti-proliferative activity (126).

Further highlighting the advantages of chemical modification, Abualhasan et al. (127) synthesized a novel series of flavonoid derivatives by incorporating aryl groups and gallic acid analogs. The molecular structures of these newly developed compounds were validated through nuclear magnetic resonance (NMR) and infrared (IR) spectroscopy. Subsequent biological assessments revealed that compound 3 acted as a potent anti-proliferative agent against Caco-2 colorectal cancer cells, with an IC_50_ of 2.42 μg mL^−1^. In contrast, compound 4 displayed notable antioxidant activity (IC_50_ = 3.53 ± 0.1 μg mL^−1^) and served as a selective cyclooxygenase-2 (COX-2) enzyme inhibitor (IC_50_ = 6.02 ± 0.33 μg mL^−1^) (127).

These collective findings provide compelling evidence that targeted chemical modifications can substantially enhance the biological activities of flavonoids. This concept is exemplified by 8-chloro-3′,4′,5,7-tetrahydroxyflavone, which has been shown to modulate the oxidative burst in activated neutrophils more effectively than its unmodified precursor. Such improved efficacy positions this compound as a promising lead candidate for the development of novel anti-inflammatory therapeutics (128).

Beyond improving potency, structural modification and metal chelation of flavonoids have direct implications for nutritional immunity, which relies on tight control of bioavailable iron, zinc, and copper at host–pathogen interfaces (129, 130). Many canonical flavonoids (quercetin, rutin, catechin, baicalin, kaempferol) chelate Fe, Cu, and Zn with high affinity, thereby scavenging redox-active metals and attenuating metal-driven oxidative damage in host tissues (131).

Appropriately tuned chelators could therefore synergize with endogenous metal-sequestering proteins (e.g., transferrin, lactoferrin, calprotectin) to starve microbes of essential metal nutrients and potentiate antimicrobial defenses, particularly when combined with structural motifs (halogens, bulky or cationic substituents) that enhance direct antibacterial activity and membrane disruption (132, 133). Conversely, overly strong or redox-active complexes may perturb host metal homeostasis, interfere with metalloenzymes, or paradoxically promote Fenton chemistry and DNA damage, underscoring the need to balance antimicrobial nutritional-immunity with preservation of host metal physiology (134).

Chemical diversification also introduces new challenges in toxicity and selectivity. While many unmodified dietary flavonoids display a wide safety margin, high concentrations or certain substitution patterns (e.g., particular metal complexes, pro-oxidant Cu(II)–reduction systems) can induce ROS generation, lipid peroxidation, hemolysis, and genotoxicity in mammalian cells (135, 136).

Lipophilic or prenylated derivatives, halogenated analogs, and metal-flavonoid complexes often show markedly higher anticancer or antimicrobial potency but may also exhibit increased off-target cytotoxicity, mitochondrial dysfunction, or interference with drug-metabolizing enzymes and transporters (137). Achieving pathogen- or tumor-selective activity, therefore, requires careful modulation of metal-binding sites, overall lipophilicity, and charge, and steric bulk around key pharmacophores to exploit differences in membrane composition, metal handling, or kinase/COX-2 dependence between diseased and healthy cells (132).

Translational progress remains constrained by poor oral bioavailability, rapid metabolism, and limited *in vivo* exposure of most flavonoid scaffolds, which persist despite improved *in vitro* potency of many derivatives (138, 139). Extensive phase II conjugation, protein binding, and microbiota-driven transformations often yield circulating metabolites with distinct and incompletely characterized activities (140).

In addition, systematic pharmacokinetic, pharmacodynamic, and toxicological datasets are lacking for the majority of synthetically optimized or metal-complexed derivatives, hampering rational dose selection and regulatory advancement (141). Promising strategies to overcome these translational barriers include designing derivatives with optimized drug-likeness and controlled metal-chelation strength; integrating nanocarrier or metal-nanoparticle delivery systems to enhance stability, tissue targeting, and local metal-modulation in infected or tumor tissues; and embedding nutritional-immunity–relevant endpoints (metal distribution, microbiome responses, immune cell function) into early preclinical evaluation (139).

Together, these considerations highlight that future generations of flavonoid derivatives should be engineered not only for maximal *in vitro* potency but also for the safe modulation of host–pathogen metal homeostasis, high selectivity for host targets, and demonstrable translational feasibility.

## 6 Biosynthesis of flavonoids

Flavonoids constitute a major class of bioactive secondary metabolites synthesized by plant-associated endophytic fungi, which often mirror or even enhance the metabolic capabilities of their host plants (142–144). This has positioned the isolation of endophytic fungi from diverse plant hosts and the characterization of their flavonoid biosynthetic pathways as key frontiers in natural product research. For example, one study examined an endophytic fungal strain (AG-10) isolated from *Portulaca oleracea* (145).

Preliminary identification of its metabolites as flavonoids was achieved using chromogenic reactions and ultraviolet spectral analysis. Subsequent taxonomic characterization identified the fungus as belonging to the genus *Fusarium*, based on a combination of colony morphology, conidiospore structure, and 18S rDNA sequencing (146).

In a separate investigation involving *G. biloba*, researchers employed tissue culture techniques to isolate 116 endophytic fungal strains from the plant's roots and stems. Metabolite screening using chromogenic assays and HPLC revealed two strains with high flavonoid-producing potential (147). Spectrophotometric analysis showed that the total flavone content in their fermentation broths exceeded 20 mg. Using internal transcribed spacer (ITS) region sequencing in conjunction with morphological characteristics in phylogenetic analysis, these promising isolates were classified into the genera *Penicillium* and *Mucor*. Notably, this study represented the first report of a *Penicillium* species functioning as a flavonoid-producing endophyte in *G. biloba* (148).

Similarly, Zhou et al. (149) isolated and purified endophytic fungi from the roots, stems, and leaves of *Cinnamomum camphora* (spring variety) using tissue culture techniques. Screening of these isolates identified ten fungal strains capable of flavonoid biosynthesis, among which strain YZ-29 demonstrated a notably high flavonoid production capacity, highlighting its potential as a valuable microbial source for natural flavonoid synthesis (132).

Cheng et al. (150) utilized *Gentiana straminea* to further expand the recognized diversity of flavonoid-producing endophytes to characterize an endophytic fungal strain (Gs-6). This strain was identified as *Cadophora* sp. through an integrated approach combining morphological characterization and molecular phylogenetic analysis. Biochemical assessment confirmed its ability to synthesize distinct flavonoids, including isovitexin (0.824 mg L^−1^), quercetin (1.110 mg L^−1^), and isoorientin (1.569 mg L^−1^), thereby establishing *Cadophora* sp. as a promising microbial source of plant-derived polyphenolics (150).

Furthermore, an endophytic fungal strain (YS-101) isolated from *Saussurea involucrata* was screened for flavonoid production using chromogenic reactions and thin-layer chromatography (TLC). The strain was taxonomically identified as *Aspergillus tabacinus*, and subsequent HPLC quantification revealed that the rutin content in a 200 mL potato dextrose broth (PDB) culture exceeded 9.0 μg, confirming its capability to biosynthesize flavonoids under laboratory conditions (132).

The biosynthetic pathway for flavonoids is well known and involves two distinct metabolic pathways. The acetate (polyketide) pathway makes the A-ring by using three molecules of malonyl-CoA that come from glucose metabolism. At the same time, the shikimate pathway provides the B-ring and a C3 linking unit, which comes from the aromatic amino acid phenylalanine. Enzymes turn phenylalanine into 4-coumaroyl-CoA, which is the most important building block that starts the flavonoid pathway and builds the C6–C3–C6 carbon skeleton (151).

A pivotal step in flavonoid biosynthesis is catalyzed by chalcone synthase (CHS), which mediates the condensation of the A-ring and B-ring precursors to produce chalcone, the first committed intermediate in the pathway. This chalcone is subsequently isomerized into dihydroflavone by the enzyme chalcone isomerase (CHI).

Dihydroflavonoids represent critical metabolic branch points, channeling carbon flux toward the formation of diverse flavonoid subclasses through the action of specialized enzymes such as flavonol synthase (FNS), isoflavone synthase (IFS), and flavonoid 3′-hydroxylase (F3′H) (152). This central biosynthetic framework is supported by several upstream enzymes that supply essential precursors, which collectively drive the formation of 4-coumaroyl-CoA, the substrate entering the CHS-catalyzed step (151).

The plant *Ampelopsis grossedentata* is highly esteemed for its wide-ranging medicinal and culinary applications, a value primarily derived from its rich flavonoid content, complemented by significant levels of amino acids, vitamins, and trace elements (153, 154). In a notable advancement of flavonoid research, Hao et al. (132) elucidated key aspects of flavonoid metabolism in *Ampelopsis grossedentata* by employing reverse transcription polymerase chain reaction (RT-PCR) to clone the *CYP73A* gene, which encodes the enzyme trans-cinnamate 4-monooxygenase (C4H). Their findings confirmed the gene's essential role within the flavonoid biosynthetic pathway of *A. grossedentata*. This research laid the groundwork for building a genetic transformation system that expresses CYP73A and for creating a vector to overexpress this gene, marking a significant step toward achieving enhanced flavonoid production in this pharmaceutically valuable plant species (132).

In a related study, Jin et al. (155) applied integrated multi-omics analyses in conjunction with genetically segregated populations to identify two critical oxygen-methyltransferases, namely *Camellia sinensis* flavonoid O-methyltransferase 1 (CsFAOMT1) and *Camellia sinensis* flavonoid O-methyltransferase 1 (CsFAOMT2). These enzymes were shown to catalyze the biosynthesis of methylated catechin, providing crucial insight into the mechanistic basis for methylated catechin derivative formation in tea plants (*Camellia sinensis*). This discovery not only deepens understanding of flavonoid modification in plants but also offers valuable genetic targets for metabolic engineering and synthetic biology applications aimed at optimized flavonoid production (155).

Substantial progress has been made in the heterologous biosynthesis of flavonoids using genetically engineered microorganisms. For example, Tang et al. (156) constructed a specialized *Escherichia coli* strain designed for the targeted synthesis of baicalein or norbaicalein, utilizing phenylalanine or tyrosine as the sole precursor. The researchers reconstructed the apigenin biosynthetic pathway in *E. coli* by introducing six heterologous genes from various species, including parsley (4CL, FNS I), red yeast (PAL), petunia (CHS), and alfalfa (CHI). Production of baicalein and norbaicalein was subsequently achieved by incorporating F6H from *S. baicalensis* and AtCPR from *Arabidopsis thaliana*. To optimize yields, the framework included overexpression of malonyl-CoA and fatty acid synthesis genes (acs, fabF) and the introduction of *matB* and *matC* from *Rhizobium trifolii*. This metabolic engineering strategy successfully yielded final titers of 23.6 mg L^−1^ for baicalein and 106.5 mg L^−1^ for norbaicalein (157).

Metabolic engineering applied directly to plants has likewise produced remarkable results. A research team led by the renowned scientist Cathie Martin demonstrated that overexpression of the *AtMYB12* transcription factor from *A. thaliana* in tomato (*Solanum lycopersicum*) significantly enhanced the accumulation of flavonoids and other phenolic compounds, reaching concentrations of approximately 100 mg g^−1^ of dry plant mass (158).

Biotechnological advances have significantly boosted flavonoid production. In tomato, introduction of Delila and Rosea1 transcription factors from *Antirrhinum majus* enhanced anthocyanin accumulation, while expression of *Arabidopsis AtMYB12* yielded flavonoid and hydroxycinnamic acid esters comprising roughly 10% of fruit dry weight (159).

At the same time, microbial platforms have proven highly effective. For example, a yeast-based cell factory made 26.57 mg L^−1^ of kaempferol directly from glucose (160), and a modular *E. coli* co-culture system made 79.0 mg L^−1^ of the pharmacologically active flavanone sakuranetin by dividing the pathway between two optimized strains (161). The integration of *Trollius chinensis* C-glycosyltransferase with *Glycine max* sucrose synthase in a biocatalytic cascade represented a significant advancement in enzymatic methodologies. The method produced very high concentrations: 7,090 mg L^−1^ of orientin and 5,050 mg L^−1^ of vitexin. It also achieved near-complete conversion (162).

Despite these advancements, the overall efficiency of flavonoid biosynthesis remains constrained by the complexity of natural product metabolic networks and the suboptimal performance of plant-derived genetic components in microbial hosts. Overcoming these limitations will require advanced strategies in promoter engineering, codon optimization, and pathway balancing to enable scalable, high-yield industrial production.

## 7 Metabolism of flavonoids

**Figure 3:**
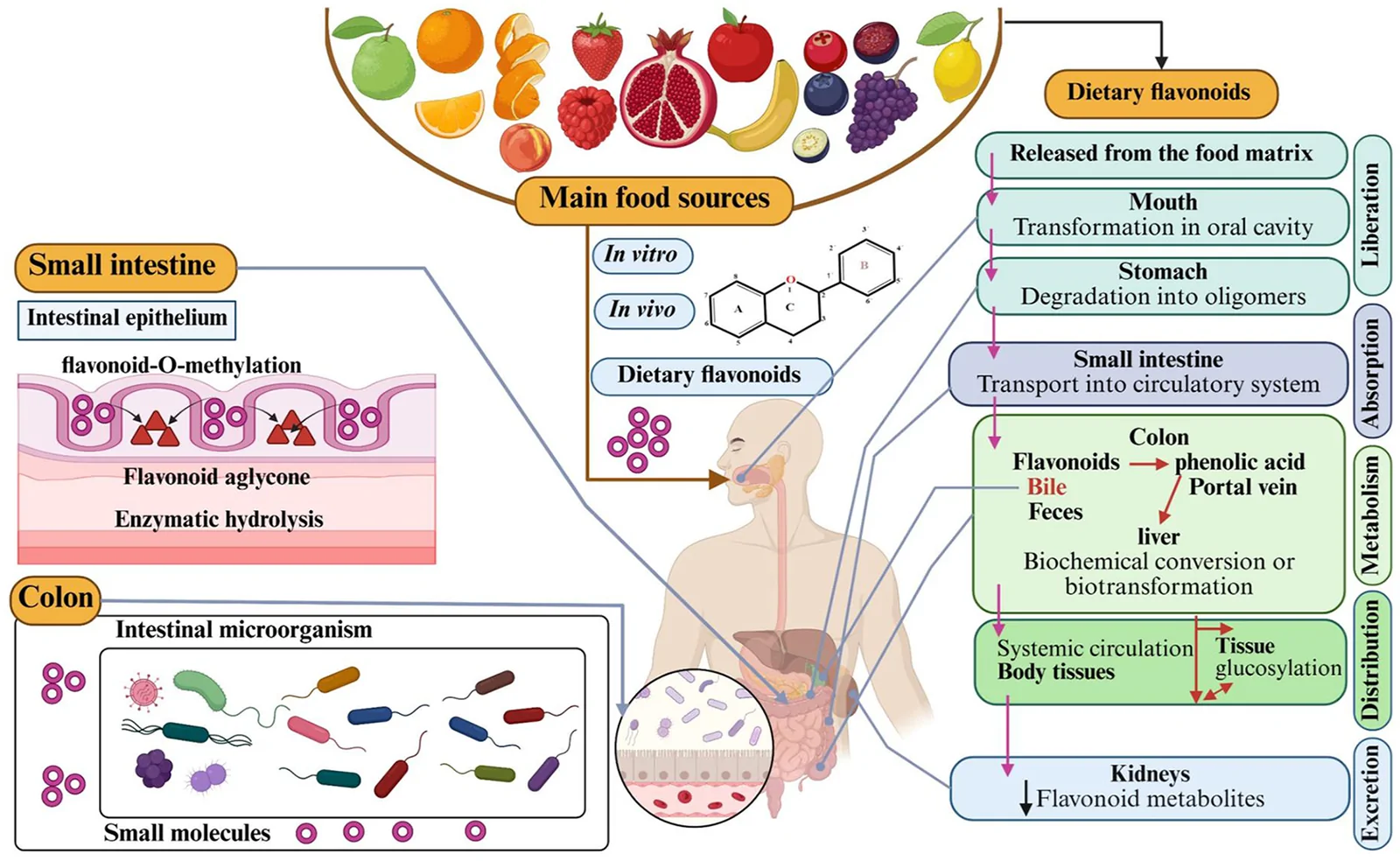
Metabolism and bioavailability of flavonoids.

For food-derived bioactive compounds to exert their intended physiological effects, efficient absorption and subsequent metabolism are indispensable prerequisites (163). Consequently, a thorough knowledge of dietary flavonoid metabolism and absorption within the digestive tract is vital for elucidating their biological functions, health benefits, and potential applications in nutrition and medicine (7, 164). Figure 3 depicts the metabolism of flavonoids.

### 7.1 Absorption of flavonoids by the small intestine

The intestines and liver are the principal sites of flavonoid metabolism, with absorption mechanisms differing markedly between aglycone and glycosylated forms (165). Owing to their hydrophobic nature and relatively low molecular weight, most aglycones, including polymethoxyflavonoids, are readily passively diffused into the small intestinal villous epithelial cells, allowing efficient entry into the systemic circulation (166, 167).

This absorption process is further supported by a variety of specialized membrane transporters located within the enterocytes of the small intestine. The principal transporter families implicated in the uptake, intracellular trafficking, and systemic distribution of flavonoids include the organic anion transporters (OATs), the solute carrier (SLC) families 22A and 21A, and the multidrug resistance-associated proteins (MRPs) (168).

These transport systems collectively regulate the bioavailability and metabolic fate of flavonoids following ingestion (169). In contrast, most dietary flavonoids exist in glycosylated forms, in which the flavonoid aglycone is conjugated to one or more sugar moieties. This glycosylation increases both the molecular weight and hydrophilicity of the compounds, thereby hindering direct passive absorption across the intestinal epithelium (170).

Although certain flavonoid glycosides can be absorbed intact, the predominant absorption route involves enzymatic hydrolysis, releasing the aglycone prior to uptake. Within the small intestine, two key enzymes are primarily responsible for the processing of flavonoid monoglucosides. The first is lactase phlorizin hydrolase (LPH), a brush-border membrane enzyme that cleaves the sugar moiety, releasing the corresponding aglycone. The liberated aglycone can then be absorbed by intestinal epithelial cells, frequently facilitated by transporters such as the sodium–glucose cotransporter 1 (SGLT1), and may undergo further hydrolysis by cytosolic broad-specific β-glucosidase (CBG) to complete the deglycosylation process (164).

### 7.2 Transformation and absorption of flavonoids in the large intestine

Consequently, the structural characteristics of the attached sugar moieties, including their chemical composition, number, and position of attachment on the flavonoid backbone, serve as critical determinants of the compound's absorption efficiency in the small intestine. These structural variations significantly influence enzyme recognition, transport affinity, and ultimately the bioavailability of dietary flavonoids (169, 171).

For example, *in vitro* studies have shown that *Blautia* sp. MRG-PMF1 possesses an O-methyltransferase enzyme capable of demethylating curcumin, thereby enhancing its metabolic conversion (172). Similarly, members of the Enterobacteriaceae family produce β-glucosidase and α-rhamnosidase enzymes that hydrolyze flavonoid glycosides into smaller, absorbable phenolic acids. Molecular docking analyses have further demonstrated that equol, a metabolite derived from isoflavones through the activity of *Lactobacillus paracasei* JS1, can be absorbed in the colon and function as a postbiotic compound with notable biological effects (173). Given its central role in the biotransformation of dietary components, the gut microbiota is now widely regarded as a key determinant of the bio-efficacy of functional foods, shaping both the metabolic fate and physiological impact of flavonoid compounds in the host (173).

The metabolic fate of flavonoids is strongly shaped by an individual's unique gut microbiome composition. The microbial community cleaves the flavonoid core structure, inducing ring fission and other chemical modifications that ultimately govern their subsequent absorption (169). This metabolic pathway frequently involves cross-feeding, a synergistic interaction in which the metabolic byproduct of one bacterial species serves as a substrate for another (171).

A representative example is the sequential hydrolysis of flavonoid glycosides by *Enterobacter*-derived α-rhamnosidase and β-glucuronidase, followed by their conversion into chalcones via flavonoid-cleaving reductases from *Clostridium* species. In turn, flavonoids can modulate the composition of the gut microbiota, often stimulating the growth of beneficial bacterial strains. This bidirectional interaction highlights the potential of specific flavonoids to act as prebiotics, thereby promoting a more favorable and resilient gut ecosystem (174–177).

### 7.3 Metabolism of flavonoids in the liver

Following absorption, flavonoids and their primary metabolites are transported to the liver via the portal vein (178). Serving as the central site of biotransformation, the liver subjects these compounds to extensive modification through two main pathways: oxidative reactions and conjugation processes (179).

Oxidative metabolism is primarily mediated by cytochrome P450 enzymes, with the CYP1A2 and CYP3A4 isoforms playing pivotal roles. These enzymes catalyze the oxidative demethylation of several flavonoids, for instance, the conversion of sakuranetin to naringenin, kaempferide to kaempferol, and tamarixetin to quercetin, as demonstrated in studies employing human liver microsomes (180, 181).

Conjugation, the second major metabolic pathway, entails the enzymatic attachment of hydrophilic moieties, such as glucuronic acid, sulfate, or methyl groups, to polar functional sites on the flavonoid structure. These reactions, including glucuronidation, sulfation, and methylation, markedly enhance the water solubility of the resulting metabolites, thereby promoting their efficient excretion (182).

A notable secondary phenomenon is auto-inhibition, in which certain flavonoids suppress the activity of the very metabolic enzymes responsible for their own biotransformation. This self-inhibitory effect can slow their metabolic rate, leading to a prolonged half-life, increased systemic exposure, and potentially enhanced therapeutic efficacy (181, 183). Following hepatic processing, the resulting metabolites re-enter the systemic circulation for distribution to target tissues, after which they are ultimately excreted by the kidneys in the urine (166).

### 7.4 Bioavailability of flavonoids

Despite their considerable potential to promote human health, the clinical use of dietary flavonoids is markedly constrained by their extremely poor oral bioavailability. A striking example is the flavonoid morin, which exhibits an absolute oral bioavailability of only 0.45% (184). This widespread limitation primarily stems from the complex molecular architecture of flavonoids, which are typically characterized by multiple aromatic rings and numerous hydroxyl groups (164).

Further compounding this issue, flavonoids undergo extensive hepatic metabolism following ingestion. This biotransformation often produces bioactive derivatives that exhibit reduced therapeutic efficacy compared to their parent compounds. To address these limitations, research efforts have focused on several key strategies, including targeted delivery systems, enhancement of intestinal absorption, and direct molecular modification (166).

Targeted delivery mechanisms are designed to transport flavonoid compounds directly to their intended physiological sites, thereby enhancing therapeutic efficacy while minimizing pre-target degradation. This approach encompasses advanced techniques such as nanoparticle formulation, carrier complexation, and solid dispersion. Among these, nanotechnology, particularly nanoencapsulation, has demonstrated exceptional promise, significantly improving both the bioavailability and biological activity of flavonoids (166, 185, 186).

Complexation with chelating agents constitutes another promising strategy, as it alters the physicochemical properties of flavonoids, thereby enhancing their stability and facilitating site-specific release (187). A well-documented example is curcumin, which readily forms complexes with a variety of biomolecules, including proteins, carbohydrates, lipids, and other natural compounds such as resveratrol and quercetin. These complexes have been consistently reported to enhance both their biological activity and bioavailability (188).

Solid dispersion, a widely adopted pharmaceutical strategy, involves dispersing a hydrophobic flavonoid within a hydrophilic, inert carrier matrix. This technique markedly improves the solubility and dissolution rate of poorly soluble compounds, ultimately enhancing bioavailability (189). A second key strategy focuses on enhancing the intestinal absorption of flavonoids. The concurrent administration of flavonoids with specific dietary constituents can yield additive or synergistic health benefits, while potentially mitigating associated cellular toxicity. For example, co-administration of luteolin with doxorubicin has been shown to promote autophagy in U2OS cells, whereas co-administration of silymarin with doxorubicin significantly suppresses proliferation in HepG2 cells (190).

Moreover, specific dietary components can modulate the intestinal environment, thereby influencing flavonoid absorption. For instance, maltitol consumption has been shown to reduce flavonol absorption compared to sucrose, whereas ethanol enhances the uptake of flavanoids by intestinal epithelial cells and facilitates flavonoid transport via the GLUT2 transporter (191, 192). Lastly, direct molecular modification of the flavonoid scaffold provides a straightforward approach to optimizing its physicochemical and biological properties. Glycosylation, the attachment of sugar moieties, can enhance flavonoid stability, increase water solubility, and modulate both its pharmacokinetic behavior and antioxidant potential (193).

Similarly, methylated flavonoids, such as 5,7-dimethoxyflavone and 3′,4′-dimethoxyflavone, exhibit markedly greater metabolic stability than their polyhydroxylated counterparts, a property that leads to substantially improved bioavailability (194). Figure 3 illustrates the bioavailability of flavonoids.

The clinical translation of flavonoids is primarily limited by their low oral bioavailability, often reported to be less than 10% for many high-potential compounds (195). This limitation is not merely a result of poor aqueous solubility but is driven by a combination of rapid first-pass metabolism in the liver and intestinal mucosa, and the active expulsion of these molecules by ATP-binding cassette (ABC) efflux transporters (196, 197).

Specifically, transporters such as P-glycoprotein (P-gp) and multidrug resistance-associated proteins (MRPs) on the apical side of enterocytes recognize flavonoids as substrates, pumping them back into the intestinal lumen and preventing systemic absorption (198). Furthermore, many flavonoids are chemically unstable, prone to oxidation and degradation under the varying pH conditions of the gastrointestinal tract (195).

To bypass these biological hurdles, this review examines the design of an advanced nano drug delivery system (NDDS) that encapsulates flavonoids within protective matrices (Table 2), i.e.

**Table 2:** Comparative analysis of advanced nano drug delivery system (NDDS) for enhancing flavonoid bioavailability: architectures, performance advantages, and translational hurdles.

Delivery system	Composition	Primary advantages	Limitations and translational hurdles
Liposomes	Phospholipid bilayers.	Excellent biocompatibility; can carry both hydrophilic and lipophilic flavonoids.	Low encapsulation efficiency for some polyphenols; physical instability (leakage) during storage.
Polymeric nanoparticles	Synthetic polymers such as poly (lactic-co-glycolic acid) (PLGA) or natural (Chitosan) polymers.	Highly stable; provides controlled and sustained release over long periods.	Potential toxicity of synthetic polymer degradation products; complex, multi-step synthesis.
Solid lipid nanoparticles (SLN)	Solid fats/lipids at room temperature.	Protects flavonoids from chemical degradation; avoids first-pass metabolism via lymphatic uptake.	Limited drug loading capacity; risk of “drug expulsion” during lipid crystallization over time.
Nanostructured lipid carriers (NLC)	Blend of solid and liquid lipids.	Higher drug loading than solid lipid nanoparticles; improved long-term stability and minimal drug leakage.	Relatively new technology; requires more robust long-term toxicity and regulatory data.
Nanoemulsions/ micelles	Surfactants and oil/water phases.	Significantly increases aqueous solubility; simple to manufacture at a laboratory scale.	High surfactant concentration may cause gastrointestinal irritation; sensitive to pH changes.

(i)- Lipid-based nanocarriers: Including solid lipid nanoparticles (SLNs) and nanostructured lipid carriers (NLCs), which offer superior biocompatibility and can facilitate lymphatic transport, thereby avoiding first-pass hepatic metabolism (199).

(ii)- Polymeric systems: Such as self-assembled micelles and nanospheres, which enhance the solubility of hydrophobic flavonoids and provide sustained-release kinetics, maintaining therapeutic concentrations over longer durations (200).

(iii)- Surface-modified formulations: Where ligands or polymers like PEG are used to increase circulation time and target specific tissue microenvironments (201).

Despite the promising performance of these platforms in preclinical models, significant hurdles remain in industrial scale-up and regulatory standardization. Achieving batch-to-batch reproducibility under good manufacturing practice (GMP) standards is complex for multi-component nanosystems. Consequently, while nanotechnology offers a pathway to overcome bioavailability limitations, the transition from laboratory prototypes to standardized clinical therapies requires more robust, longitudinal evidence of their long-term safety and pharmacokinetic predictability in humans.

## 8 Biological activities of flavonoids

### 8.1 Antioxidant activity

Flavonoids are widely recognized for their potent antioxidant and free radical–scavenging properties, as shown in Table 3. Their antioxidant action primarily arises from donating hydrogen atoms to neutralize reactive free radicals. The efficiency of this hydrogen atom transfer is critical, as molecular configurations that facilitate faster, more facile hydrogen donation correspond to stronger antioxidant activity. Consequently, flavonoids possessing multiple hydroxyl groups within their structure are considered among the most powerful natural antioxidants (202).

**Table 3:** Antioxidant activity of flavonoids.

Subclass (with examples)	Study trial	Common natural sources	Primary mechanisms of antioxidant action	Observed outcomes	Reference
Flavanols (e.g., epigallocatechin gallate, epicatechin, catechin, and gallocatechin)	*In vivo*	Green tea (*Camellia sinensis*), cocoa, grapes, apples, and berries.	Potent radical scavengers and metal chelators activate Nrf2 signaling, thereby upregulating SOD, CAT, GPx, and HO-1.	Decrease lipid peroxidation, LDL oxidation, and oxidative DNA damage. Increase glutathione and plasma antioxidant capacity in animals and humans.	(389, 390)
Anthocyanidins/anthocyanins (e.g., Cyanidin, delphinidin, malvidin, pelargonidin, peonidin, and petunidin)	*In vivo*	Berries (blueberry, blackberry, strawberry), grapes, red cabbage, and purple sweet potato.	Donate electrons to neutralize ROS, form stable metal complexes, activate Nrf2, and suppress NF-κB signaling.	Lowering oxidative stress markers improves vascular and endothelial function and enhances antioxidant enzyme activity.	(391)
Flavanones (e.g., hesperidin, naringenin, naringin, and eriodictyol)	*In vivo*	Citrus fruits (oranges, lemons, grapefruits), tomatoes, and oregano.	Scavenges radicals, chelates metals, inhibits xanthine and NADPH oxidases, and stimulates the Nrf2–ARE pathway.	Reduce lipid peroxidation and oxidative metabolites. Improve mitochondrial and antioxidant status in cardiometabolic models.	(392, 393)
Flavonols (e.g., quercetin, kaempferol, myricetin, isorhamnetin, and fisetin)	*In vivo*	Onions, apples, kale, broccoli, berries, and tea.	Neutralize ROS, chelate transition metals, inhibit oxidases, and enhance HO-1 via Nrf2.	Reduce lipid and DNA oxidation, elevate antioxidant enzyme levels, and protect cardiovascular and neural tissues.	(394–396)
Isoflavones (e.g., Genistein, daidzein, glycitein, biochanin A, and formononetin)	*In vivo*	Soybeans, chickpeas, red clover, and legumes.	Act as phytoestrogens activating ER–Nrf2 and AMPK pathways. Suppresses oxidative enzymes.	Reduce oxidative DNA damage and increase antioxidant enzyme activity. Modestly raises serum antioxidant capacity in humans.	(397, 398)
Flavones (e.g., Apigenin, luteolin, baicalein, chrysin, and tangeretin)	*In vivo*	Parsley, celery, chamomile, *Scutellaria baicalensis* (Chinese skullcap), and citrus peel.	Donate hydrogen/electrons, inhibit xanthine oxidase. Activate Nrf2 and stabilize mitochondria.	Decrease in ROS generation and lipid peroxidation. Protect skin and neurons against oxidative injury.	(394, 399)
Chalcones (e.g., Xanthohumol, licochalcone A, isoliquiritigenin, and butein)	*In vivo*	Hops (*Humulus lupulus*), licorice (*Glycyrrhiza* spp.), *Angelica keiskei* (ashitaba), and *Alpinia* species.	It possesses a reactive α, β-unsaturated carbonyl structure. It scavenges free radicals, chelates metals, and activates Nrf2 genes.	Reduce ROS and lipid peroxidation. Elevate HO-1 and glutathione. Nanoparticle forms improve stability and bioactivity.	(400, 401)
Proanthocyanidins (e.g., Procyanidin B1/B2, prodelphinidin, and procyanidin C1)	*In vivo*	Cranberries, cocoa, grapes, apples, and pine bark.	Strong hydrogen donors and metal chelators modulate Nrf2. Inhibit NADPH oxidase.	Lower oxidative damage in plasma, liver, and kidney. They protect lipids and DNA and improve total antioxidant status *in vivo*.	(402, 403)
Dihydroflavonols (e.g., Taxifolin, aromadendrin, and dihydromyricetin)	*In vivo*	Douglas fir bark, milk thistle, citrus fruits, and grapes.	Quench ROS, chelate metals, and prevent lipid oxidation. They stabilize membranes and DNA.	Decrease MDA and ROS levels in oxidative stress models and enhance antioxidant enzyme activities.	(404, 405)
Flavonolignans (e.g., Silybin, silychristin, silydianin, and isosilybin)	*In vivo*	Milk thistle (*Silybum marianum*) seeds.	Combine flavonoid and lignan structures. Directly scavenge radicals and induce Nrf2-regulated enzymes.	Protection of hepatic cells from oxidative damage. Reduce lipid peroxidation and restore glutathione levels.	(406)
Aurones (e.g., Aureusidin, sulfuretin, 4,6-dihydroxyaurone, and 4-hydroxyaurone)	*In vitro*	Snapdragons (*Antirrhinum majus*), daisies, *Coreopsis*, and some legumes.	Contain conjugated systems allowing radical stabilization. Inhibit lipid oxidation and activate antioxidant genes.	Demonstrate potent antioxidant effects *in vitro* and protect against oxidative cytotoxicity in cell models.	(407, 408)

SOD, superoxide dismutase; CAT, catalase; GPx, glutathione peroxidase; LDL (low-density lipoprotein) cholesterol; ROS, reactive oxygen species; NF-κB, nuclear factor kappa-light-chain-enhancer of activated B cells; AMPK, AMP-activated protein kinase; MDA, malondialdehyde.

Flavonoids mitigate the complex chain reactions of lipid peroxidation through both direct and indirect mechanisms. They directly intercept and neutralize free radicals, thereby terminating propagation and halting chain reactions initiated by physicochemical stressors. By inhibiting the peroxidation of unsaturated fatty acids such as arachidonic acid, flavonoids help protect cellular membranes (203).

Additionally, they can quench various ROS, including singlet oxygen and hydroxyl radicals, often via single-electron transfer. Indirectly, flavonoids modulate radical levels in biological systems by coprecipitating with proteins, thereby enabling them to bind and inhibit radical-generating enzymes. Quercetin serves as a prominent example, mitigating oxidative damage by lowering intracellular ROS concentrations, altering signaling pathways linked to oxidative stress, and controlling glutathione-dependent enzyme activity (203).

Another critical antioxidant mechanism of flavonoids involves chelating pro-oxidant metal ions, such as Fe^2+^, which serve as potent catalysts in numerous oxidative reactions. The strong antioxidant activity of quercetin and rutin in Fe^2+^-rich systems is largely attributed to their metal-chelating capacity (204). Likewise, naringin and apigenin enhance skin health by strengthening the body's overall antioxidant defenses, as evidenced by reduced levels of malondialdehyde and lipid peroxides, along with increased CAT activity and total antioxidant capacity (204). Moreover, total flavonoids extracted from *Acer truncatum* leaves have demonstrated protective effects against juglone-induced oxidative stress. This protection is achieved through the upregulation of key antioxidant enzymes, including SOD and CAT, accompanied by a concurrent reduction in malondialdehyde and ROS levels (205).

Furthermore, flavonoids can act synergistically with other antioxidants; for instance, the efficacy of catechins is markedly enhanced in the presence of vitamins C and E. Studies on a flavonoid-rich ethanolic extract (EE) have confirmed significant *in vitro* radical-scavenging activity. The IC_50_ values for 2,2-diphenyl-1-picrylhydrazyl (DPPH), 22′-azino-bis [3-ethylbenzothiazoline-6-sulfonic acid (ABTS)], and hydroxyl radicals were 296.95 ± 13.24 μg mL^−1^, 94.31 ± 9.13 μg mL^−1^, and 9.21 ± 0.15 μg mL^−1^, respectively, indicating that the extract exhibited formidable antioxidant qualities (206). By inhibiting β-glucosidase activity and dramatically lowering intracellular ROS levels in a high-glucose-induced L02 cell culture, this ethanol extract (EE) reduced oxidative damage and inflammation (206).

*Camellia oleifera* total flavonoid glycosides (FG) have demonstrated potent DPPH radical-scavenging activity and have successfully shielded vascular endothelial cells from hydrogen peroxide (H2O_2_)-induced oxidative damage (207). The lipid peroxidation process was considerably slowed down in studies involving rat liver microsomes by 6^′′^-O-acetylgenistin (10.6 μM) and 6^′′^-O-acetyldaidzin (8.2 μM) (132, 208).

### 8.2 Nutritional immunity, anti-inflammatory, and analgesic activities

The inflammatory response is orchestrated through a complex network of signaling mechanisms, predominantly mediated by four major pathways: the interleukin-6/signal transducer and activator of transcription 3 (IL-6/STAT3), tumor necrosis factor-alpha/nuclear factor kappa-B essential modulator/inhibitor of kappa-B (TNF-α/NEMO/IκB), Toll-like receptor/nuclear factor kappa-B (Toll/NF-κB), and ROS/mitogen-activated protein kinase/NOD-like receptor protein 3/nuclear factor kappa-B (ROS/MAPK/NLRP3/NF-κB) cascades (209–211).

Although the anti-inflammatory effects of flavonoids are multifaceted, their primary mechanism of action involves interrupting the activation cascades of key inflammatory signaling pathways (212). This interference is achieved by modulating key regulatory proteins and transcription factors, ultimately reducing the secretion of pro-inflammatory mediators and cytokines. A major aspect of this mechanism is the inhibition of NF-κB pathway activity, wherein flavonoids suppress the phosphorylation and nuclear translocation of the p50 and p65 subunits and prevent the phosphorylation of IκB, thereby maintaining its inhibitory function and attenuating downstream inflammatory responses (213–215).

By preventing the activation of vital kinases such as JNK/SAPK, ERK1/2, and p38 MAPK, or by reducing the transcription of target genes such as perilipin and PDE3B, flavonoids also alter MAPK signaling. Through these actions, they effectively attenuate the propagation of inflammatory signals and the expression of pro-inflammatory mediators (216–219).

Additionally, flavonoids reduce the secretion of inducible nitric oxide synthase (iNOS) and nitric oxide in macrophages by inhibiting the phosphorylation of the upstream kinase JAK2, thereby dampening downstream pro-inflammatory signaling and nitric oxide–mediated oxidative stress (220, 221).

Furthermore, flavonoids diminish the activity of inflammatory mediators and proteins by attenuating or blocking signaling pathways associated with nuclear factor kappa-B (NF-κB), mitogen-activated protein kinase/extracellular signal-regulated kinase (MAPK/ERK), and signal transducer and activator of transcription 1 (STAT1), thereby suppressing the transcriptional activation of inflammation-related genes (222). Furthermore, vascular endothelial growth factor (VEGF), MAPK, and phosphoinositide 3-kinase/protein kinase B (PI3K/Akt) pathways are among the other important signaling pathways that flavonoids block. Critical cellular functions, including migration, angiogenesis, tumorigenesis, persistence, growth, differentiation, and proliferation, are regulated by these cascades (223, 224).

To support this, Shao et al. (225) showed that an extract from *Artemisia anomala* has anti-inflammatory properties by activating the TLR4–MyD88–NF-κB signaling pathway, which suppresses phagocytic activity, nitric oxide production, and the release of pro-inflammatory cytokines such as interleukin-6 (IL-6), interleukin-10 (IL-10), and tumor necrosis factor-alpha (TNF-α) (225).

Multiple studies have confirmed that several flavonoids, including rutin, hydroxyrutin, and dihydroquercetin, possess strong anti-inflammatory and analgesic effects (226). These compounds effectively reduce inflammation in various experimental models, including carrageenan-, 5-hydroxytryptamine (5-HT)-, and prostaglandin E2 (PGE2)-induced rat paw edema, as well as formaldehyde-induced arthritis and granuloma formation. Additionally, total flavonoid extracts from *G. biloba* demonstrated significant pain-relieving effects, highlighting their therapeutic potential (227).

The flavonoid naringenin has been shown to alleviate neuropathic pain by downregulating matrix metalloproteinase (MMP) expression and reducing the levels of TNF-α and transforming growth factor-beta 1 (TGF-β1) (228). Hesperidin exhibits intrinsic anti-inflammatory and analgesic properties, and its co-administration with nonsteroidal anti-inflammatory drugs (NSAIDs) produces a synergistic effect, enabling effective pain management at lower drug doses (229).

Its methylated derivative, hesperidin methyl chalcone (HMC), directly inhibits the NF-κB pathway in macrophages by interacting with the p65 subunit at Ser276. A reduction in arthritic pain is caused by this action because it lowers the amounts of cytokines that are reliant on NF-κB, such as IL-33, TNF-α, and IL-6 (230). Luteolin is recognized as a particularly potent anti-inflammatory agent, as demonstrated in both *in vitro* and *in vivo* studies. Its derivative, luteolin-3′-O-phosphate, exhibits efficacy comparable to, and in many cases exceeding, that of luteolin itself. This superior performance has been observed across diverse experimental models and is attributed to its stronger inhibitory effects on the MAPK and NF-κB signaling pathways (227).

Furthermore, with IC_50_ values of 16.4 μM and 10.8 μM, respectively, the flavonoid negletein exhibits substantial anti-inflammatory activity by inhibiting the production of the key pro-inflammatory cytokines TNF-α and interleukin-1 beta (IL-1β). Another substance, the homoisoflavanone sappanone A, significantly reduces nitric oxide generation triggered by lipopolysaccharide (LPS) and reduces allergic airway inflammation in an ovalbumin-induced asthma model. In BV2 and RAW264.7 cell lines, it modulates the NF-κB and heme oxygenase-1/nuclear factor erythroid 2–related factor 2 (HO-1/Nrf2) signaling pathways, thereby exerting its anti-inflammatory effect (231).

Flavonoids have a wide range of complex anti-inflammatory actions. Pectolinarin exerts its effects by inhibiting the secretion of IL-6 and interleukin-8 (IL-8), and by reducing the production of prostaglandin E2 (PGE_2_) and nitric oxide. HMC exhibits anti-inflammatory, antioxidant, and analgesic activities by suppressing cytokine production and inhibiting NF-κB activation (232).

Kaempferol 3-O-β-D-glucuronide promotes the production of the anti-inflammatory cytokine IL-10 while attenuating the expression of other pro-inflammatory mediators, such as interleukin-1 beta (IL-1β), NO, PGE_2_, and leukotriene B4 (LTB4). Broussochalcone A acts by suppressing both iron-induced lipid peroxidation and nitric oxide synthesis in (LPS)-activated macrophages (233). Research on quercetin has shown that it prevents the phosphorylation of the IκB protein, it inhibits the NF-κB signaling pathway, and reduces the inflammatory response that microglial cells experience when LPS and interferon-gamma (IFN-γ) are present (234, 235).

Moreover, quercetin and apigenin, along with their metabolites, have been shown to suppress microRNA-155 (miR-155) expression, thereby downregulating key pro-inflammatory mediators, including TNF-α, IL-6, and interleukin-1 beta (IL-1β). This regulatory effect further contributes to their overall anti-inflammatory activity (236). In BV-2 microglial cells, treatment with 6-methoxyflavone (6-MeOF) inhibited LPS-induced phosphorylation of key proteins within the NF-κB/IκB, TLR4/MyD88, and phospho-p38 MAPK/JNK signaling pathways (237).

Additionally, 6-MeOF counteracted LPS-induced elevations in nitric oxide, ROS, iNOS, and cyclooxygenase-2 (COX-2), while simultaneously enhancing the expression of the antioxidant proteins heme oxygenase-1 (HO-1) and NAD(P)H quinone dehydrogenase 1 (NQO1). The suppression of LPS–induced nitric oxide production and the strong anti-inflammatory and antioxidant activities observed are confirmed by *in vivo* studies with 6-MeOF (237).

The reduction of proinflammatory mediators and enzymes triggered by LPS, such as tumor necrosis factor-alpha (TNF-α), interleukin-6 (IL-6), nitric oxide, iNOS, and cyclooxygenase-2 (COX-2), is caused by hydrogenated isoflavones (238). Eupatilin exerts protective effects by elevating SOD, GSH, and IL-10 levels, while reducing malondialdehyde, TNF-α, interleukin-1 beta (IL-1β), and IL-6 levels. Additionally, it reduces inflammatory reactions by significantly downregulating the NF-κB signaling pathway (239).

Transcriptomic analyses of flavonoids isolated from *Abrus mollis* and *Abrus cantoniensis* provide deeper mechanistic insight, revealing that their anti-inflammatory activity is mediated through the targeted regulation of genes associated with inflammatory signaling pathways (240). In other studies, epigallocatechin gallate (EGCG) was shown to attenuate H_2_O_2_-induced inflammation and cellular damage by downregulating the cyclic GMP–AMP synthase/stimulator of interferon genes/NLRP3 (cGAS/STING/NLRP3) signaling pathway, thereby enhancing cell viability and reducing apoptosis (241).

In conclusion, flavonoids' capacity to enhance nutritional immunity stems from their ability to modulate complex intracellular signaling cascades. At the molecular level, flavonoids such as quercetin, kaempferol, and apigenin exert significant control over the NF-κB (nuclear factor kappa-light-chain-enhancer of activated B cells) pathway, a central regulator of the innate immune response. By inhibiting the phosphorylation of inhibitory κB (IκB) proteins, flavonoids prevent the translocation of NF-κB into the nucleus, thereby downregulating the expression of pro-inflammatory genes including iNOS, COX-2, and TNF-α. Simultaneously, flavonoids activate the Nrf2 (nuclear factor erythroid 2-related factor 2) signaling pathway, which triggers the transcription of phase II antioxidant enzymes. This dual action, suppressing overactive inflammatory signals while bolstering the cellular antioxidant defense, creates a balanced immunological environment. Additionally, by modulating MAPK pathways, flavonoids influence dendritic cell maturation and antigen-presenting capabilities, effectively bridging the gap between innate and adaptive nutritional immunity. Table 4 delineates the anti-inflammatory and analgesic properties of flavonoids.

**Table 4:** Anti-inflammatory and analgesic activities of flavonoids.

Subclass (with examples)	Study trial	Common natural sources	Primary anti-inflammatory and analgesic mechanisms	Observed outcomes	References
Flavanols (e.g., Epigallocatechin gallate, epicatechin, catechin, and gallocatechin)	*In vivo*	Green tea (*Camellia sinensis*), cocoa, grapes, apples, and berries.	Inhibit NF-κB and MAPK pathways. Downregulate COX-2, iNOS, TNF-α, IL-1β, and IL-6. Reduce prostaglandin and nitric oxide formation.	Decrease edema, hyperalgesia, and cytokine expression in arthritis and colitis models. Improve tissue histology and reduce inflammation-related pain in humans.	(409–411)
Anthocyanidins/anthocyanins (e.g., Cyanidin, delphinidin, malvidin, pelargonidin, peonidin, and petunidin)	Clinical	Berries (blueberry, blackberry, strawberry), grapes, red cabbage, and purple sweet potato.	Block NF-κB activation. Lower COX-2, prostaglandin E_2_, and nitric oxide. Promote anti-inflammatory M2 macrophages.	Reduce paw edema, swelling, and pain. Lower CRP, TNF-α, and IL-6. Improve vascular and systemic inflammatory biomarkers in clinical trials.	(412, 413)
Flavanones (e.g., Hesperidin, naringenin, naringin, and eriodictyol)	*In vivo*	Citrus fruits (oranges, lemons, grapefruits), tomatoes, and oregano.	Inhibit NF-κB and MAPK. Suppress COX-2, iNOS, and PGE_2_. Modulate TRP channels.	Reduce inflammatory pain, oxidative damage, and cytokine release in arthritis and gastritis models. Improve vascular inflammation in humans.	(414, 415)
Flavonols (e.g., Quercetin, kaempferol, myricetin, isorhamnetin, and fisetin)	*In vivo*	Onions, apples, kale, broccoli, berries, and tea.	Suppress NF-κB, MAPK, and STAT signaling. Inhibit COX-2, iNOS, and cytokines. Enhance Nrf2 antioxidant response.	Decrease joint swelling and inflammatory infiltration. Quercetin and kaempferol lessen hyperalgesia and oxidative stress in animal and human studies.	(416, 417)
Isoflavones (e.g., Genistein, daidzein, glycitein, biochanin A, and formononetin)	*In vivo*	Soybeans, chickpeas, red clover, and legumes.	Modulate ER-dependent NF-κB, JAK-STAT, and MAPK signaling. Reduce TNF-α, IL-1β, and oxidative stress.	Attenuate inflammation in cardiovascular and neurodegenerative models. Lower CRP and cytokines in humans, including mild immunomodulatory effects.	(418, 419)
Flavones (e.g., Apigenin, luteolin, baicalein, chrysin, and tangeretin)	*In vivo*	Parsley, celery, chamomile, *Scutellaria baicalensis* (Chinese skullcap), and citrus peel.	Inhibit NF-κB and MAPK. Suppress COX-2, iNOS, and cytokines. stabilize lysosomes and reduce capillary permeability.	Diminishes edema, nociceptive behavior, and inflammation. Apigenin protects against IBD and neuroinflammation.	(420, 421)
Chalcones (e.g., Xanthohumol, licochalcone A, isoliquiritigenin, and butein)	*In vivo*	Hops (*Humulus lupulus*), licorice (*Glycyrrhiza* spp.), *Angelica keiskei* (ashitaba), and *Alpinia* species.	Inhibit IKK/NF-κB and NLRP3 pathways. Suppress COX, LOX, and nitric oxide/prostaglandin synthesis.	Lower edema, pain, and cytokine expression. Prevent chronic inflammatory tissue degeneration. Potent scaffolds for new anti-inflammatory drugs.	(422, 423)
Proanthocyanidins (e.g., Procyanidin B1/B2, Prodelphinidin, and Procyanidin C1)	*In vivo*	Cranberries, cocoa, grapes, apples, and pine bark.	Block NF-κB and AP-1 activation. Inhibit COX-2, iNOS, and TNF-α. Enhance antioxidant defenses.	Reduce edema, inflammatory cell infiltration, and pain in arthritis and colitis models. Improve inflammatory markers in human supplementation studies.	(412, 424)
Dihydroflavonols (e.g., Taxifolin, Aromadendrin, and Dihydromyricetin)	*In vivo*	Douglas fir bark, milk thistle, citrus fruits, and grapes.	Inhibit NF-κB and NLRP3 inflammasome. Suppress COX-2 and cytokine expression.	Attenuate inflammation and oxidative damage in hepatic and neural tissues. Relieve pain and edema in animal models.	(425, 426)
Flavonolignans (e.g., Silybin, Silychristin, Silydianin, and Isosilybin)	*In vivo*	Milk thistle (*Silybum marianum*) seeds.	Downregulate NF-κB, TNF-α, and IL-6. Inhibit COX-2 and promote antioxidant enzyme activity.	Reduce hepatic and systemic inflammation. Relieve pain and oxidative stress in liver injury and arthritis models.	(427, 428)
Aurones (e.g., Aureusidin, Sulfuretin, 4,6-Dihydroxyaurone, and 4-Hydroxyaurone)	*In vivo*	Snapdragons (*Antirrhinum majus*), daisies, *Coreopsis*, and some legumes.	Inhibit NF-κB and COX-2 expression. Modulate cytokine and ROS production.	Exhibit anti-edematous and analgesic activity in inflammatory pain models. Protect tissues from oxidative injury.	(132)

MAPK, mitogen-activated protein kinase; NF-κB, nuclear factor kappa-light-chain-enhancer of activated B cells; iNOS, nitric oxide synthase; CRP, high C-reactive protein; ROS, reactive oxygen species.

### 8.3 Anti-cancer activity

A major focus of contemporary flavonoid research is their potential as anticancer agents. Chowdhury et al. (242) reported that linarigenin induces cell cycle arrest in human lung carcinoma (A549) and hepatoma (HepG2) cell lines under *in vitro* conditions. This effect is mediated by the upregulation of endogenous cyclin-dependent kinase (CDK) inhibitors, along with increased expression of the tumor suppressor genes *p53* and *p21* (243).

In a related study, Gupta et al. (244) reported that apigenin induces G_1_-phase cell cycle arrest in human prostate cancer (LNCaP) cells. This arrest is characterized by a marked reduction in the levels of cyclins D1, D2, and E, along with their associated cyclin-dependent kinases (CDK2, CDK4, and CDK6), accompanied by an upregulation of the CDK inhibitors *p21* and *p27* (244). A similar mechanism was observed in human bladder cancer (T-24) cells, where apigenin treatment increased the levels of phosphorylated p53 (p-p53), total p53, p21, and p27 proteins. These molecular alterations resulted in the downregulation of cyclins A, B1, and E, as well as cyclin-dependent kinases CDK1 and CDK2 and the phosphatase Cdc25C, ultimately inducing G1-phase arrest and suppressing tumor cell proliferation (245).

Further *in vitro* supporting evidence from Hsu et al. (246) demonstrated that baicalein modulates key cell cycle regulators in murine cardiac endothelial cells, leading to arrest at both the G1 and G_2_ phases. This impact was amplified by the upregulation of p15, p21, p53, and cyclin E, and the downregulation of cyclin D2, cyclin A, CDK1, and CDK2 (246). Baicalein was also shown to induce S-phase arrest in lung squamous cell carcinoma and to trigger apoptosis in human lung cancer (CH27) cells. Cyclin-dependent kinase 4 (CDK4), cyclin B1, and cyclin D1 are downregulated, leading to S-phase arrest, interrupting the cell cycle and preventing tumor cell growth (247).

Nobiletin inhibits the growth of human U87 and Hs683 glioma cells by inducing G0/G1-phase cell-cycle arrest, according to research by Lien et al. (248). Inhibition of the Akt and MAPK signaling pathways results in the downregulation of important cell cycle regulators, such as cyclin D1, CDK2, CDK4, and the transcription factor E2F transcription factor 1 (E2F1). Moreover, nobiletin impedes glioma cell migration, further contributing to its overall antitumor efficacy (248).

*Glycine max* and its derivatives are rich in soybean isoflavones, a subclass of flavonoids, which are predominantly composed of daidzin and genistin. These compounds exert estrogen-mimetic effects that contribute to breast cancer protection. However, this activity is manifested only after their biotransformation into the active aglycone forms, daidzein and genistein, through gut microbial metabolism or gastric hydrolysis (249).

Emerging research indicates that the anticancer effects of soybean isoflavones extend beyond their estrogenic activity. For instance, they have the ability to alter how cancer cells express the copper transporter genes CTR1 and ATP7A, thereby interfering with metal ion homeostasis and tumor cell proliferation (250).

Beyond their well-established antioxidant role in protecting against oxidative DNA damage, soybean isoflavones also inhibit tumor progression by directly inducing apoptosis in cancer cells and downregulating oncogene expression. Collectively, through these multifaceted mechanisms, soybean isoflavones represent promising candidates for adjuvant chemotherapeutic applications and may provide therapeutic benefits in the management of malignancies such as ovarian cancer (251, 252).

Apigenin has been shown to promote autophagy by upregulating key autophagic proteins, including Beclin-1, ULK1, ATG5, ATG13, and LC3B. Interestingly, this enhancement of autophagy occurs concurrently with the suppression of key signaling molecules, including AMP-activated protein kinase (AMPK), mechanistic target of rapamycin (mTOR), p70S6 kinase (P70S6K), and ATG4. In addition to its autophagy-inducing activity, apigenin exhibits potent anti-proliferative effects and suppresses tumor growth *in vivo* by triggering ferroptosis, a regulated form of iron-dependent cell death (253).

In an *in vitro* study, Zhang et al. (254) reported that the flavonoid chrysin induces G_2_/M-phase cell cycle arrest in human esophageal squamous carcinoma (KYSE-510) cells, ultimately leading to apoptosis. This effect is mechanistically linked to the upregulation of the tumor suppressor proteins p21 and p53, accompanied by a reduction in cyclin B1 expression (254).

Similarly, human colon cancer (SW480) and stomach adenocarcinoma (SGC7901) cells have been shown to undergo time-dependent apoptosis in response to quercetin. The underlying mechanism involves suppression of both phosphorylated and total STAT3 protein levels, as well as decreased STAT3 mRNA expression. This downregulation consequently reduces survivin expression (255). In gastric cancer cells, quercetin further enhances apoptosis by modulating the balance of Bcl-2 family proteins, downregulating the anti-apoptotic protein Bcl-2 while upregulating the pro-apoptotic protein Bax, thereby lowering the Bcl-2/Bax ratio (255).

A comparable pro-apoptotic shift, marked by increased Bax expression and decreased Bcl-2 levels, has also been observed in human promyelocytic leukemia (HL-60) cells following quercetin treatment (256–258). Oroxylin A exhibits a broad spectrum of anticancer activities, including the induction of apoptosis, inhibition of tumor invasion and metastasis, disruption of glycolytic metabolism, suppression of cellular proliferation and angiogenesis, reversal of drug resistance, and induction of cell cycle arrest (259).

In a separate study, the ability of human breast epithelial MCF10A-ras cells to proliferate is inhibited by eupatilin in a dose- and time-dependent manner. This inhibition is linked to higher levels of p53 and p27 and lower levels of cyclin D1, cyclin B1, CDK1, and CDK2. It seems that inhibiting the Raf/MEK/ERK signaling pathway is the cause of the drop in cyclin D1 (260). In cervical cancer (HeLa) cells, eupafolin induces G_2_/M-phase cell-cycle arrest and promotes apoptosis. The early growth response 1 (Egr-1)–dependent Bax signaling pathway mediates this effect by decreasing cyclin D1 and increasing cyclin B1, p53, and p21 levels (261).

Apoptotic cell death and cell cycle arrest at the S and G_2_/M phases result from molecular changes caused by luteolin, which decreases Akt, polo-like kinase 1 (PLK1), cyclin B1, cyclin A, CDK1, CDK2, and Bcl-xL, and increases p21 and Bax, specifically in estrogen receptor–negative MDA–MB−231 breast cancer cells (262). Furthermore, kaempferol exhibits potential in preventing DNA damage and inhibiting cancer cell proliferation, effects that may be attributed to its strong anti-inflammatory and antioxidant properties (263).

**Figure 4:**
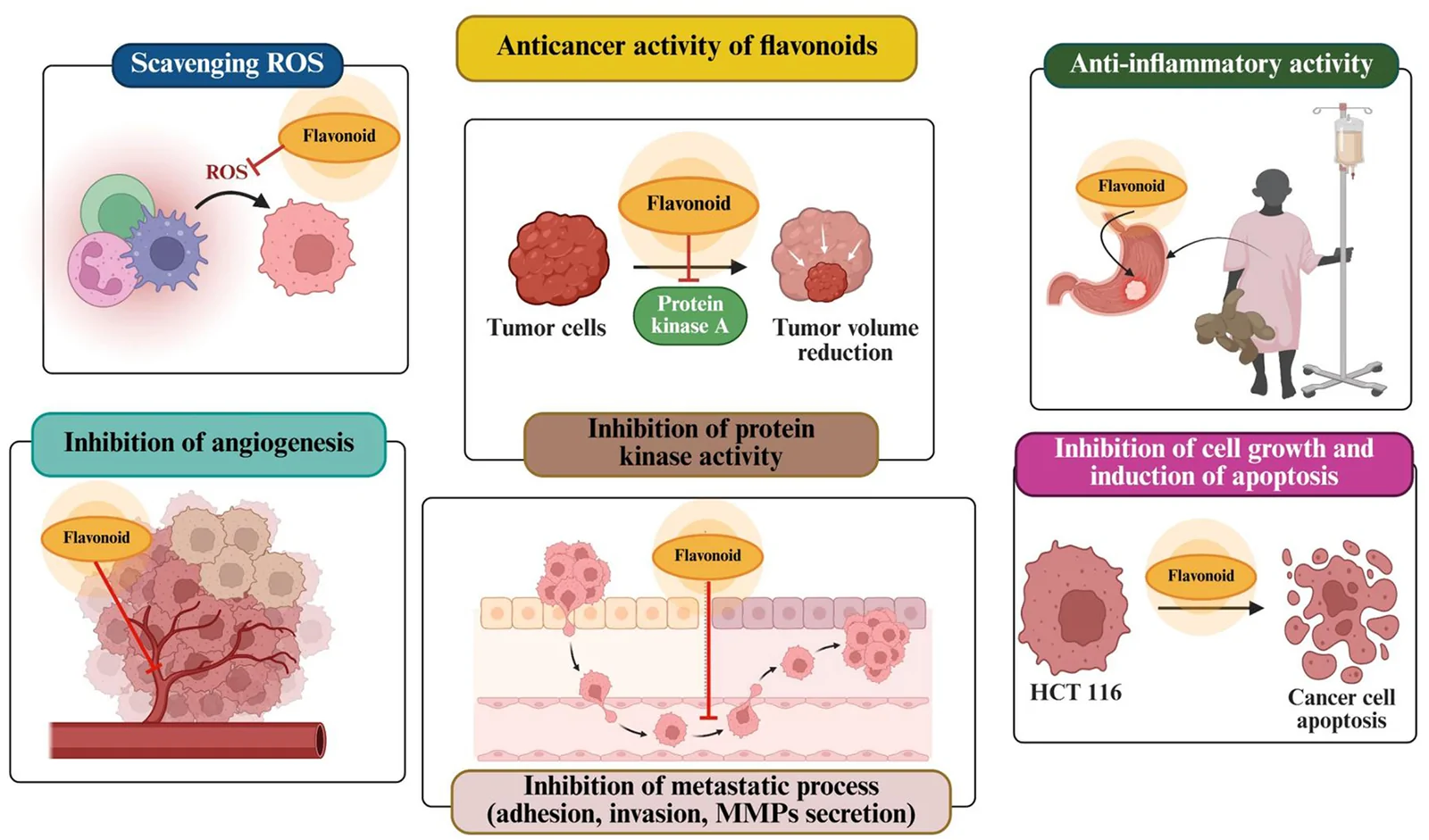
Anticancer activity of flavonoids. ROS, reactive oxygen species; MMP, matrix metalloproteinase.

In a separate study, Wang et al. (264) demonstrated that oroxylin A induces cancer cells to release extracellular vesicles that promote M1-like macrophage polarization, an immunomodulatory response mediated *in vitro* through caspase-3–dependent activation of Rho-associated protein kinase 1 (ROCK1). Figure 4 depicts the anticancer properties of flavonoids, and the anticancer action of flavonoids is demonstrated in Table 5.

**Table 5:** Anti-cancer activity of flavonoids.

Subclass (with examples)	Study trial	Common natural sources	Primary anti-cancer mechanisms	Observed outcomes	References
Flavanols (e.g., Epigallocatechin gallate, epicatechin, catechin, and gallocatechin)	*In vitro*	Green tea (*Camellia sinensis*), cocoa, grapes, apples, and berries.	Inhibit PI3K/Akt/mTOR and MAPK pathways. Induce G1/G2 arrest, apoptosis via caspase activation. Suppress VEGF-mediated angiogenesis.	EGCG reduces tumor size and metastasis, enhances chemosensitivity, and decreases microvessel density in breast, prostate, and colorectal cancers.	(429, 430)
Anthocyanidins/anthocyanins (e.g., Cyanidin, delphinidin, malvidin, pelargonidin, peonidin, and petunidin)	*In vitro*	Berries (blueberry, blackberry, strawberry), grapes, red cabbage, and purple sweet potato.	Block NF-κB and AP-1, downregulate COX-2 and MMP-9, activate caspase-3. Prevent DNA adducts and oxidative mutagenesis.	Inhibit tumor incidence, angiogenesis, and metastasis *in vivo*. Suppress invasion of breast, colon, and lung cancer cells. Strong chemopreventive potential.	(431)
Flavanones (e.g., Hesperidin, naringenin, naringin, and eriodictyol)	*In vitro*	Citrus fruits (oranges, lemons, grapefruits), tomatoes, and oregano.	Modulate PI3K/Akt, MAPK, and p53 pathways. Promote apoptosis. Inhibit migration, angiogenesis, and metastasis. Act as selective pro-oxidant sensitizers.	Decrease tumor volume and inflammatory signaling. Enhance chemotherapy response. Low toxicity supports adjuvant therapy.	(432, 433)
Flavonols, Flavones (e.g., Quercetin, kaempferol, myricetin, isorhamnetin, apigenin, acacetin, baicalein, luteolin, tangeretin, and wogonin and fisetin)	*In vitro*	Onions, apples, kale, broccoli, berries, parsley, celery, red pepper, and tea.	Regulate Bax/Bcl-2 ratio, activate caspase-3/9, arrest cell cycle. Inhibit PI3K/Akt/mTOR, MAPK/ERK, and Wnt/β-catenin. Suppress VEGF and MMPs. Induce intrinsic and extrinsic apoptosis via caspase activation, arrest cell cycle at G1 or G2/M phases, and inhibit PI3K/Akt/mTOR, MAPK/ERK, and Wnt/β-catenin pathways.	Quercetin and kaempferol suppress tumor proliferation and invasion. Increase apoptosis and chemosensitivity in breast, lung, and prostate cancers. Suppress tumor proliferation, inhibit migration and invasion across multiple cell lines, and enhance chemotherapy efficacy through chemosensitization.	(434, 435)
Isoflavones (e.g., Genistein, daidzein, glycitein, biochanin A, and formononetin)	*In vivo*	Soybeans, chickpeas, red clover, and legumes.	Modulate endoplasmic reticulum-dependent signaling. Inhibit tyrosine kinases and induce G_2_/M arrest and caspase-mediated apoptosis. Regulate DNA methylation and histone acetylation.	Reduce the proliferation of hormone-dependent cancers. Lower prostate-specific antigen (PSA) levels and hormone-driven tumor risk. Show chemopreventive effects in human studies.	(436, 437)
Flavones (e.g., Apigenin, luteolin, baicalein, chrysin, and tangeretin)	*In vitro*	Parsley, celery, chamomile, *Scutellaria baicalensis* (Chinese skullcap), and citrus peel.	Inhibit PI3K/Akt/mTOR and MAPK. Downregulate Bcl-2, induce p53, block EMT, and angiogenesis. Helps trigger apoptosis as necessary.	Apigenin and luteolin reduce tumor weight, suppress metastasis, and enhance chemotherapeutic efficacy in xenograft and cell models.	(438, 439)
Chalcones (e.g., Xanthohumol, licochalcone A, isoliquiritigenin, and butein)	*In vitro*	Hops (*Humulus lupulus*), licorice (*Glycyrrhiza* spp.), *Angelica keiskei* (ashitaba), and *Alpinia* species.	Inhibit tubulin polymerization, topoisomerase, tyrosine kinase, and NF-κB. Induce oxidative stress, apoptosis, and autophagy. Suppress angiogenesis.	Lower cancer incidence and metastasis. Trigger apoptosis in liver, colon, and breast tumors. Chalcone derivatives serve as anti-proliferative drug leads.	(440, 441)
Proanthocyanidins (e.g., Procyanidin B1/B2, prodelphinidin, and procyanidin C1)	*In vitro*	Cranberries, cocoa, grapes, apples, and pine bark.	Inhibit PI3K/Akt and NF-κB signaling. promote apoptosis and DNA repair. Block angiogenesis and tumor cell adhesion.	Reduce tumor growth and metastasis in colon, skin, and breast cancers. Enhance chemotherapeutic effects and antioxidant protection.	(442)
Dihydroflavonols (e.g., Taxifolin, aromadendrin, and dihydromyricetin)	*In vitro*	Douglas fir bark, milk thistle, citrus fruits, and grapes.	Modulate MAPK and p53 pathways. induce apoptosis via caspase activation. Inhibit proliferation and oxidative DNA damage.	Suppress tumor growth and metastasis in hepatocellular and melanoma models. Improve chemotherapy response.	(443, 444)
Flavonolignans (e.g., Silybin, silychristin, silydianin, and isosilybin)	*In vitro*	Milk thistle (*Silybum marianum*) seeds.	Inhibit PI3K/Akt and JAK/STAT. block NF-κB. induce p53 and apoptosis. Inhibit angiogenesis and tumor invasion.	Decrease tumor progression and oxidative damage in liver, lung, and prostate models. Exhibit chemopreventive and chemosensitizing effects.	(442)
Aurones (e.g., Aureusidin, sulfuretin, 4,6-dihydroxyaurone, and 4-hydroxyaurone)	*In vitro*	Snapdragons (*Antirrhinum majus*), daisies, *Coreopsis*, and some legumes.	Act as ROS modulators. inhibit COX-2, MMP-9, and NF-κB. Trigger intrinsic apoptosis and DNA damage repair mechanisms.	Show growth-inhibitory and pro-apoptotic effects in breast, colon, and melanoma cell lines. Emerging as novel anti-cancer scaffolds.	(445)

MAPK, mitogen-activated protein kinase; NF-κB, nuclear factor kappa-light-chain-enhancer of activated B cells; EGCG, epigallocatechin gallate; MMPs, matrix metalloproteinase; ROS, reactive oxygen species.

### 8.4 Anti-anxiety activity

Flavonoids exhibit significant therapeutic potential for anxiety disorders, primarily owing to their actions within the central nervous system (265). Animal studies have consistently demonstrated the strong potential of dietary phytoconstituents, particularly flavonoids, in alleviating anxiety (Table 6). One of the primary mechanisms underlying the anxiolytic effects of many compounds involves the positive modulation of gamma-aminobutyric acid type A (GABA) receptors, often through binding at the benzodiazepine (BZD) site or other associated allosteric sites (265).

**Table 6:** Anti-anxiety activity of flavonoids.

Subclass (with examples)	Study trial	Common natural sources	Primary anti-anxiety mechanisms	Observed outcomes	References
Flavanols (e.g., Epigallocatechin gallate, epicatechin, catechin, and gallocatechin)	*In vivo*	Green tea (*Camellia sinensis*), cocoa, grapes, apples, and berries.	Enhance GABAergic transmission. Inhibit monoamine oxidase. Increase dopamine and serotonin. Lower oxidative and inflammatory stress in neurons.	EGCG reduces anxiety-like behavior and corticosterone levels. Improves cognition. Produces calming effects like diazepam in animal and human studies.	(446)
Anthocyanidins/anthocyanins (e.g., Cyanidin, delphinidin, malvidin, pelargonidin, peonidin, and petunidin)	*In vivo*	Berries (blueberry, blackberry, strawberry), grapes, red cabbage, and purple sweet potato.	Alleviate neuroinflammation and oxidative stress. Increase serotonin and dopamine. Upregulate brain-derived neurotrophic factor (BDNF) and neuronal plasticity.	Decrease anxiety-related behaviors and improve mood. Restore neurotransmitter balance in stress models. Anthocyanin-rich diets improve mood in limited human trials.	(447)
Flavanones (e.g., Hesperidin, naringenin, naringin, and eriodictyol)	*In vivo*	Citrus fruits (oranges, lemons, grapefruits), tomatoes, and oregano.	Bind to the benzodiazepine site on GABA(A) receptors. Enhance GABAergic transmission. modulate serotonin and dopamine. Suppress neuroinflammation.	Hesperidin and naringenin reduce anxiety behaviors, prolong sleep, and decrease corticosterone. Citrus flavanones exhibit mild tranquilizing effects without dependency.	(448, 449)
Flavonols (e.g., Quercetin, kaempferol, myricetin, isorhamnetin, and fisetin)		Onions, apples, kale, broccoli, berries, and tea.	Normalize HPA axis activity. enhance GABA and serotonin signaling. Reduce neuroinflammation and oxidative stress.	Quercetin lowers anxiety and cortisol. Improves brain antioxidant enzyme activity. High flavonol intake is linked with reduced anxiety and depression symptoms.	(450)
Isoflavones (e.g., Genistein, daidzein, glycitein, biochanin A, and formononetin)	*In vivo*	Soybeans, chickpeas, red clover, and legumes.	Bind estrogen receptors in the brain. Enhance serotonin production. Modulate neuroendocrine and inflammatory balance.	Soy isoflavones improve anxiety and mood in menopausal and preclinical models. The benefits are mild but consistent in estrogen-deficient states.	(451, 452)
Flavones (e.g., Apigenin, luteolin, baicalein, chrysin, and tangeretin)	*In vivo*	Parsley, celery, chamomile, *Scutellaria baicalensis* (Chinese skullcap), and citrus peel.	Potentiate GABA(A) receptor function. Inhibit monoamine oxidase. Enhance serotonergic tone. Reduce neuroinflammation.	Apigenin, chrysin, and baicalein reduce anxiety and stress responsiveness. Increase open-arm exploration. Show benzodiazepine-like action with high safety.	(453, 454)
Chalcones (e.g., Xanthohumol, licochalcone A, Isoliquiritigenin, and butein)	*In vivo*	Hops (*Humulus lupulus*), licorice (*Glycyrrhiza* spp.), *Angelica keiskei* (ashitaba), and *Alpinia* species.	Modulate GABAergic signaling. Suppress NF-κB and NLRP3 activation. Reduce oxidative and inflammatory stress in the central nervous system.	Xanthohumol and isoliquiritigenin decrease anxiety and immobility. Enhance exploratory behavior. Promising neuroprotective scaffolds for central nervous system drug development.	(423, 455)
Proanthocyanidins (e.g., Procyanidin B1/B2, prodelphinidin, and procyanidin C1)	*In vivo*	Cranberries, cocoa, grapes, apples, and pine bark.	Protect neurons via antioxidant and anti-inflammatory actions. Modulate serotonin and GABA levels. Support neurogenesis.	Decrease anxiety-like behaviors and oxidative markers in hippocampal tissues. Improve learning and memory in stress models.	(456, 457)
Dihydroflavonols (e.g., Taxifolin, aromadendrin, and dihydromyricetin)	*In vivo*	Douglas fir bark, milk thistle, citrus fruits, and grapes.	Reduce neuroinflammation and oxidative damage. Enhance dopaminergic and serotonergic signaling. Stabilize the hypothalamic-pituitary-adrenal (HPA) axis.	Decrease anxiety-related behaviors and neuronal oxidative stress. Protect dopaminergic neurons in animal models.	(456)
Flavonolignans (e.g., Silybin, silychristin, silydianin, and isosilybin)	preclinical	Milk thistle (*Silybum marianum*) seeds.	Attenuate neuroinflammation and oxidative damage. Modulate serotonin, dopamine, and GABA signaling.	Reduce stress-induced anxiety and improve cognition in rodent models. Shown neuroprotective potential in preclinical data.	(458)
Aurones (e.g., Aureusidin, sulfuretin, 4,6-dihydroxyaurone, and 4-hydroxyaurone)	*In vivo*	Snapdragons (*Antirrhinum majus*), daisies, *Coreopsis*, and some legumes.	Act as monoamine oxidase (MAO) inhibitors and GABA modulators. Protect neurons from oxidative injury. Regulate neuroinflammatory signaling.	Exhibit mild anxiolytic and antidepressant-like activity. Improve behavioral responses in stress and anxiety tests *in vivo*.	(459)

GABAA receptor, gamma-aminobutyric acid type A receptor; NF-κB, nuclear factor kappa-light-chain-enhancer of activated B cells; EGCG, epigallocatechin gallate.

For instance, the flavonoid chrysin acts as a competitive ligand at the BZD site of the GABAA receptor, with a Ki of 3 μmol L^−1^. Intraperitoneal administration of a 1 mg kg^−1^ dose in mice produced a pronounced anxiolytic effect. Similarly, apigenin demonstrated anxiolytic activity at a dose of 3 mg kg^−1^ by binding to the BZD site of the GABAA receptor complex, thereby enhancing GABAergic neurotransmission (266).

Wogonin exhibits a high affinity for BZD receptors, with a Ki value of 0.92 μmol L^−1^. Electrophysiological analyses have confirmed that wogonin potentiates the GABA-induced chloride current. This potentiation is selectively blocked by the BDZ receptor antagonist Ro15-1788, confirming that its effects are mediated through this specific receptor pathway (267). In a similar manner, 6-hydroxyflavonoids (6HF) were found to enhance GABA-activated currents in rat cortical neurons through benzodiazepine receptors. The anxiolytic effect of 6HF was abolished by flumazenil, a GABA receptor antagonist, further confirming that its activity is mediated via BZD receptor modulation (268).

According to Ito et al. (269) the hesperidin and its aglycone, hesperetin, significantly reduced immobility time in mice during open-field tests at a dose of 50 mg kg^−1^, without affecting voluntary locomotion. This finding suggests an anxiolytic efficacy comparable to fluoxetine, with hesperetin slightly more potent than hesperidin. According to the theory, the anxiolytic effect of hesperidin may result from its metabolic conversion into hesperetin (269).

Recent reviews have identified 15 natural flavonoid monomers with confirmed anti-anxiety effects, primarily belonging to the flavone, flavonol, and dihydroflavone subclasses, with flavones being the most prevalent. Structure–activity relationship (SAR) analyses indicate that certain structural features, such as the presence of a 2′-hydroxyl group, an 8-methoxy substitution, or a substitution at the 6-position, enhance anxiolytic potency. Conversely, an increased number of hydroxyl groups tends to reduce activity. A key finding is that flavonoid aglycones generally exhibit significantly stronger anxiolytic effects than their corresponding glycosides (269, 270).

These structural insights are vital for advancing the development of flavonoid-based anxiolytic agents and for optimizing the therapeutic use of flavonoid-rich medicinal plants. In one study, a hydroxylic extract of *Spinacia oleracea*, rich in flavonoids and administered at 200 mg kg^−1^, produced anxiolytic effects in the elevated plus maze (EPM) model comparable to those of the standard drug diazepam (2 mg kg^−1^) (270).

Research has identified at least 15 natural flavonoid monomers with demonstrated anti-anxiety properties, including chrysin, apigenin, wogonin, baicalein, and luteolin (270). A critical structural feature shared by these active compounds is the presence of hydroxyl groups at the 5 and 7 positions on the A-ring. Variations in their anxiolytic potency are associated with specific substitutions, such as hydroxyl or methoxy groups, at the 6- and 8-positions on the A-ring or the 3′- and 4′-positions on the B-ring. Empirical studies provide strong evidence for their efficacy. In the EPM, administration of chrysin (1 mg kg^−1^) significantly increased both the number of entries into and the time spent in the open arms. A higher 3 mg kg^−1^ dose also enhanced exploratory behavior in the hole-board test, without inducing muscle relaxation (271).

Similarly, orally administered kaempferol, at doses exceeding 0.02 mg kg^−1^, produced marked anxiolytic effects in mice (272), an outcome that was likewise confirmed for quercetin (273). Further studies confirmed the anxiolytic efficacy of kaempferol (1.0 mg kg^−1^) and quercetin (0.5 and 1.0 mg kg^−1^) in the EPM, whereas myricetin did not produce a significant effect (274). In contrast, both acute and chronic administration of ellagic acid have been shown to induce anxiety-like behavior in mice in the same experimental model (275). The anti-anxiety activity of flavonoids is demonstrated in Table 6.

### 8.5 Antidiabetic activity

Diabetes is widely acknowledged as one of the three most serious diseases posing a major threat to global human health (276). This complex metabolic disorder affects multiple organ systems, prompting extensive research into natural compounds that can lower blood glucose levels. Among these, flavonoids have emerged as a major focus of scientific investigation due to their broad spectrum of pharmacological activities (277).

Flavonoids exert a powerful glucose-lowering effect through a coordinated, multi-targeted mechanism that counteracts the fundamental physiological dysfunctions underlying hyperglycemia, as shown in Table 7. Their actions are multifaceted and often synergistic, encompassing stimulation of insulin secretion from pancreatic β-cells, regulation of enzymes involved in glucose metabolism (278).

**Table 7:** Antidiabetic activity of flavonoids.

Subclass (with examples)	Study trial	Common natural sources	Primary antidiabetic mechanisms	Observed outcomes	References
Flavanols (e.g., Epigallocatechin gallate, Epicatechin, Catechin, and Gallocatechin)	*In vivo*	Green tea (*Camellia sinensis*), cocoa, grapes, apples, and berries.	Activate AMPK and promote GLUT4 translocation. inhibit α-amylase and α-glucosidase. Protect β-cells from oxidative injury.	EGCG lowers fasting glucose and HbA1c, improves insulin release and β-cell recovery. Human studies show enhanced glucose tolerance and antioxidant status.	(409, 460, 461)
Anthocyanidins/Anthocyanins (e.g., Cyanidin, Delphinidin, Malvidin, Pelargonidin, Peonidin, and Petunidin)	*In vivo*	Berries (blueberry, blackberry, strawberry), grapes, red cabbage, and purple sweet potato.	Inhibit α-glucosidase and DPP-4. enhance insulin signaling and glycogen synthesis. Suppress oxidative and inflammatory stress.	Reduce fasting glucose and HbA1c, elevate insulin levels, and improve glucose tolerance. Human studies link higher intake with reduced diabetes risk.	(462, 463)
Flavanones (e.g., Hesperidin, Naringenin, Naringin, and Eriodictyol)	Clinical	Citrus fruits (oranges, lemons, grapefruits), tomatoes, and oregano.	Inhibit α-amylase and α-glucosidase. activate AMPK. Enhance GLUT4 expression and protect β-cells.	Hesperidin and naringenin decrease glucose and lipids, increase insulin release, and improve oxidative balance in diabetic models. Mild improvements were observed clinically.	(464, 465)
Isoflavones (e.g., Genistein and Daidzein)	*In vivo*	Soy and legumes	Stimulate GLUT4-mediated glucose uptake. activate AMPK. Suppress hepatic gluconeogenesis and oxidative stress.	Reduces glucose, HbA1c, and insulin resistance. Improves β-cell function and lipid profile in both animal and human studies.	(466)
Isoflavones (e.g., Genistein, Daidzein, Glycitein, Biochanin A, and Formononetin)	Clinical	Soybeans, chickpeas, red clover, and legumes.	Activate ER-dependent AMPK and PPAR signaling. Promote β-cell proliferation and inhibit apoptosis. Lower hepatic glucose output.	Genistein decreases fasting glucose and enhances insulin sensitivity. Soy isoflavones improve glycemic and metabolic indices in diabetic patients.	(467, 468)
Flavones (e.g., Apigenin, Luteolin, Baicalein, Chrysin, and Tangeretin)	*In vivo*	Parsley, celery, chamomile, *Scutellaria baicalensis* (Chinese skullcap), and citrus peel.	Inhibit intestinal glucose absorption. activate AMPK. Prevents AGE formation. Protect β-cells from oxidative and cytokine injury.	Apigenin, luteolin, and baicalein lower blood glucose and lipids, improve antioxidant defense, and enhance insulin secretion in diabetic models.	(469, 470)
Chalcones (e.g., Xanthohumol, Licochalcone A, Isoliquiritigenin, and Butein)	*In vivo*	Hops (*Humulus lupulus*), licorice (*Glycyrrhiza* spp.), *Angelica keiskei* (ashitaba), and *Alpinia* species.	Activate AMPK. increase glucose uptake and fatty-acid oxidation. Inhibit aldose reductase and improve insulin signaling.	Decrease fasting glucose and lipid levels. Restore hepatic and pancreatic antioxidants. xanthohumol prevents diabetic nephropathy and retinopathy.	(471)
Proanthocyanidins (e.g., Procyanidin B1/B2, Prodelphinidin, and Procyanidin C1)	*In vivo*	Cranberries, cocoa, grapes, apples, and pine bark.	Improve insulin sensitivity by activating AMPK. Suppress carbohydrate-digesting enzymes. Protect β-cells from oxidative stress.	Reduce blood glucose and HbA1c, restore antioxidant status, and protect the liver and pancreas. Prevents complications of diabetes *in vivo*.	(472)
Flavonolignans (e.g., Silybin, Silychristin, Silydianin, and Isosilybin)	*In vivo*	Milk thistle (*Silybum marianum*) seeds.	Activate PPAR-γ and AMPK. Inhibit gluconeogenesis. Protect β-cells via antioxidant mechanisms.	Decrease glucose and HbA1c. Improve insulin sensitivity and reduce diabetic complications in clinical and animal studies.	(473, 474)
Aurones (e.g., Aureusidin, Sulfuretin)	*In vivo/In vitro*	Snapdragons, daisies, and Coreopsis.	Inhibits MAO-B and aldose reductase to reduce oxidative stress and neuroinflammation in the brain.	Reduces stress-induced anxiety behaviors and protects neurons from metabolic-driven damage and cognitive decline.	(475)

MAPK, mitogen-activated protein kinase; NF-κB, nuclear factor kappa-light-chain-enhancer of activated B cells; EGCG, epigallocatechin gallate; MMPs, matrix metalloproteinase; ROS, reactive oxygen species.

This approach involves modulating insulin-signaling proteins to enhance insulin sensitivity and promote glucose uptake in peripheral tissues, such as skeletal muscle and adipose tissue. It also aims to reduce oxidative stress and inflammation, improve lipid metabolism to prevent lipotoxicity, and protect renal tissues from diabetic nephropathy. Activating vital signaling pathways, including AMPK, peroxisome proliferator-activated receptor γ (PPARγ), and PI3K/Akt in the liver and adipose tissue, as well as controlling enzymes involved in hepatic glycolysis and gluconeogenesis, are important methods (278).

Extensive molecular studies back these mechanisms. For instance, fisetin helps regulate glucose levels and enhance insulin sensitivity by inhibiting the expression of two crucial hepatic gluconeogenic enzymes: phosphoenolpyruvate carboxykinase (PEPCK) and glucose-6-phosphatase (G-6-Pase). Research in diabetic rat models demonstrates that fisetin not only reduces blood glucose levels but also increases liver glycogen storage and activates key glycolytic enzymes, including hexokinase, pyruvate kinase, and glucose-6-phosphate dehydrogenase (279, 280).

Similarly, both quercetin and kaempferol reduce hepatic glucose synthesis and intestinal starch digestion, improve skeletal muscle glucose uptake, and protect pancreatic β-cells from damage, thereby exerting their antidiabetic effects. Research by Zang et al. (281) demonstrated that apigenin has been shown to strongly stimulate AMPK phosphorylation in HepG2 liver cells, exhibiting approximately 200 times greater potency than metformin. It also reduces lipid accumulation under high-glucose conditions by inhibiting acetyl coenzyme A carboxylase (ACC) phosphorylation. Since mitochondrial dysfunction plays a key role in insulin resistance, apigenin's ability to maintain mitochondrial health and protect hamster insulinoma tumor (HIT-T15) pancreatic β-cells is of significant (282).

Furthermore, both apigenin and luteolin have been shown to inhibit NF-κB activation and iNOS expression, thereby preventing cytokine-induced suppression of insulin secretion by inflammatory mediators, including interleukin-1β (IL-1β) and interferon-γ (IFN-γ) (283). The antidiabetic effects of flavonoids are also evident in various plant extracts. For instance, the leaf extract of *Belamcanda chinensis*, rich in flavonoids such as swertisin and mangiferin, has demonstrated notable hypoglycemic efficacy (284).

Swertisin, in particular, has been shown to stimulate insulin secretion, promote the conversion of glucose to fructose, and inhibit aldose reductase activity within the polyol pathway, thereby contributing to improved glycemic control (285). Studies on apigenin, lignans, and baicalein in insulin-resistant (IR) HepG2 cells show that these compounds improve glucose uptake and promote glycogen synthesis by activating glucose transporter protein 4 (GLUT4) and increasing the phosphorylation of glycogen synthase kinase-3β (GSK-3β). They also significantly decrease the production of ROS and advanced glycation end products (AGEs), effects linked to the inhibition of the NF-κB/P65 pathway (224).

Yang et al. (286) investigated the effects of total flavonoids from *Hippophae rhamnoides* (TFH) in a rat model of type 2 diabetes mellitus (T2DM), and revealed that TFH alleviates diabetic symptoms by improving lipid profiles and suppressing inflammation. These beneficial outcomes were mediated through the downregulation of protein kinase C alpha (PRKCA), MAPK10, and the p65 subunit of TNF-α, accompanied by a reduction in diacylglycerol (DAG) levels within the DAG/PRKCA/MAPK10/TNF-α/p65 signaling pathway (286).

In a similar vein, the flavonoids apigenin, luteolin, and baicalein have been shown to significantly inhibit the generation of ROS and the formation of advanced glycation end-products (AGEs). This inhibitory action is closely linked to the suppression of NF-κB activation and the phosphorylation of its p65 subunit, thereby establishing a direct molecular connection between flavonoid intake and the mitigation of oxidative and inflammatory stress (285).

The actions of individual flavonoids further underscore the diversity of their therapeutic targets. For example, studies in diabetic rats have revealed that naringenin increases the production of heat shock proteins (HSP-72 and HSP-27) and peroxisome proliferator-activated receptor γ (PPARγ). This molecular cascade ultimately enhanced the phosphorylation of insulin receptor substrate 1 (IRS1) at Tyr612, leading to lower blood glucose levels and improved insulin sensitivity (287).

Puerarin, an isoflavone, exhibits a broad range of hypoglycemic effects, partly by reducing oxidative stress and improving mitochondrial function through modulating the AMPK signaling pathway (288). Numerous investigations have shown that puerarin causes hypoglycemia via upregulating the expression of insulin-like growth factor-1 (IGF-1), insulin receptor substrate-1 (IRS-1), and peroxisome proliferator-activated receptor α (PPARα) (289).

Several puerarin-induced effects include improved hepatic function, protection of pancreatic β-cells, and lowering of blood glucose levels. These include downregulating the expression of uncoupling protein 2 (UCP2) mRNA, activating the AKT signaling pathway downstream of the insulin receptor, and phosphorylating glycogen synthase kinase-3β (GSK-3β) (290).

Puerarin improves glucagon-like peptide-1 (GLP-1) signaling by increasing the levels of the GLP-1 receptor (GLP-1R) and the transcription factor pancreatic and duodenal homeobox-1 (PDX-1). In diabetic mice given a high-fat diet, this activation causes protein kinase B (Akt) to be stimulated, which deactivates FOXO1 and improves glucose tolerance, β-cell proliferation, β-cell destruction, and body weight (291). Genistein is another well-known isoflavone that directly affects pancreatic β-cells by activating the cAMP/protein kinase A (PKA) signaling pathway. This stimulation enhances the number of insulin receptors, encourages the healing of injured β-cells, and boosts the manufacture of insulin (292).

In diabetic rat models, biochanin A has been shown to control body weight gain, reduce blood lipid levels, preserve pancreatic integrity, and normalize hepatic enzyme activities, including AST, ALT, and alkaline phosphatase (ALP) (293). As a class, insulin signaling pathways and related molecular targets in the liver and adipose tissue are modulated by isoflavonoids, which are known to maintain glucose homeostasis mainly by preserving β-cell mass and function (293).

Specifically, biochanin A mitigates oxidative stress and lipid abnormalities by enhancing glucose uptake through the upregulation of visfatin expression, a mechanism crucial for managing obesity-associated diabetes. Additionally, EGCG, a major flavonoid in green tea, exhibits both direct and indirect antidiabetic effects. It can modulate nitric oxide synthase activity in skeletal muscle to promote vasodilation and exerts an insulin-mimetic effect on glucose metabolism, further contributing to improved glycemic control (294).

Moreover, EGCG activates AMP-activated protein kinase (AMPK) and peroxisome proliferator-activated receptor gamma coactivator 1-alpha (PGC-1α), which in turn inhibit acetyl-CoA carboxylase (ACC) activity and enhances the expression of glucose transporter 4 (GLUT4). Through these mechanisms, EGCG effectively ameliorates insulin resistance and improves metabolic homeostasis (295, 296).

Beyond modulating established metabolic pathways, certain flavonoids show a remarkable ability to promote the formation of new insulin-producing cells. Swertisin acts as an inducer of islet differentiation, encouraging the conversion of pancreatic stem or progenitor cells into functional pancreatic β-cells. This complex process involves the activation of multiple signaling pathways, including p38 MAPK, neuregulin-3, the Smad protein cascade, and the MEK-ERK pathways. Collectively, these mechanisms demonstrate swertisin's potential as an antidiabetic agent that stimulates endogenous β-cell regeneration (297).

In conclusion, this body of research demonstrates that the primary hypoglycemic mechanism of flavonoids lies in their ability to preserve β-cell viability, improve insulin sensitivity and overall energy metabolism by activating important metabolic signaling pathways in the liver and adipose tissue. Nonetheless, comprehensive *in vivo* studies examining the antidiabetic efficacy of flavonoids remain relatively limited.

Current research is increasingly focused on identifying the precise molecular targets through which flavonoids exert their antidiabetic effects *in vivo*. Through the inhibition of hepatic gluconeogenesis, reduction of insulin resistance, prevention of lipid accumulation and oxidation, and suppression of the enzymatic activities of glucose-6-phosphatase (G-6-Pase), phosphoenolpyruvate carboxykinase (PEPCK), and peroxisome proliferator-activated receptor gamma coactivator 1-alpha (PGC-1α), anthocyanins regulate the metabolism of glucose and lipids (298).

Given that anthocyanins are widely present in common dietary sources, their strategic incorporation into daily nutrition offers a practical and promising approach for the prevention and management of diabetes and its associated metabolic complications. The anti-diabetic activity of flavonoids is demonstrated in Table 7.

### 8.6 Antibacterial activity

Novel antibacterial agents derived from natural sources are of paramount importance due to the growing public health concern posed by antibiotic-resistant bacteria. A study by Wang et al. (299) successfully isolated several flavonoids from *Ziziphus jujuba* using semi-HPLC (299).

*Escherichia coli, Shigella*, and *Pseudomonas aeruginosa* were effectively inhibited by quercetin, which showed the broadest antibacterial spectrum in antimicrobial assays. The minimum inhibitory concentration (MIC) values determined were 125 μg mL^−1^ for *Shigella*, and 250 μg mL^−1^ for both *E. coli* and *P. aeruginosa*. Notably, rutin and hyperin displayed superior potency against *E. coli*, each with an MIC of 62.5 μg mL^−1^, while hyperin showed the greatest activity against *Bacillus subtilis* (MIC 250 μg mL^−1^) (300).

Further investigation demonstrated that the antibacterial activity of these flavonoids is pH-dependent, most effective under acidic conditions (pH <6) and gradually diminishing as alkalinity increases. The presence of metal ions, including Na^2+^, Ca^2+^, K^+^, Fe^2+^, and Mg^2+^, was found to enhance the antimicrobial efficacy of several compounds. Specifically, these ions potentiated quercetin's activity against *E. coli, Shigella*, and *P. aeruginosa*, as well as rutin's action against *E. coli*. Among them, calcium and potassium ions were particularly effective in amplifying hyperin's antibacterial potency (301).

Collectively, these findings highlight the significant potential of *Ziziphus jujuba*-derived flavonoids as natural antibacterial agents, offering promising applications in both the food preservation and pharmaceutical industries (299). Supporting this, Górniak et al. (302) reported that the flavonoid gancaonin G exhibits potent antibacterial activity against both *Streptococcus mutans* and methicillin-resistant *Staphylococcus aureus* (MRSA) strains (302).

**Figure 5:**
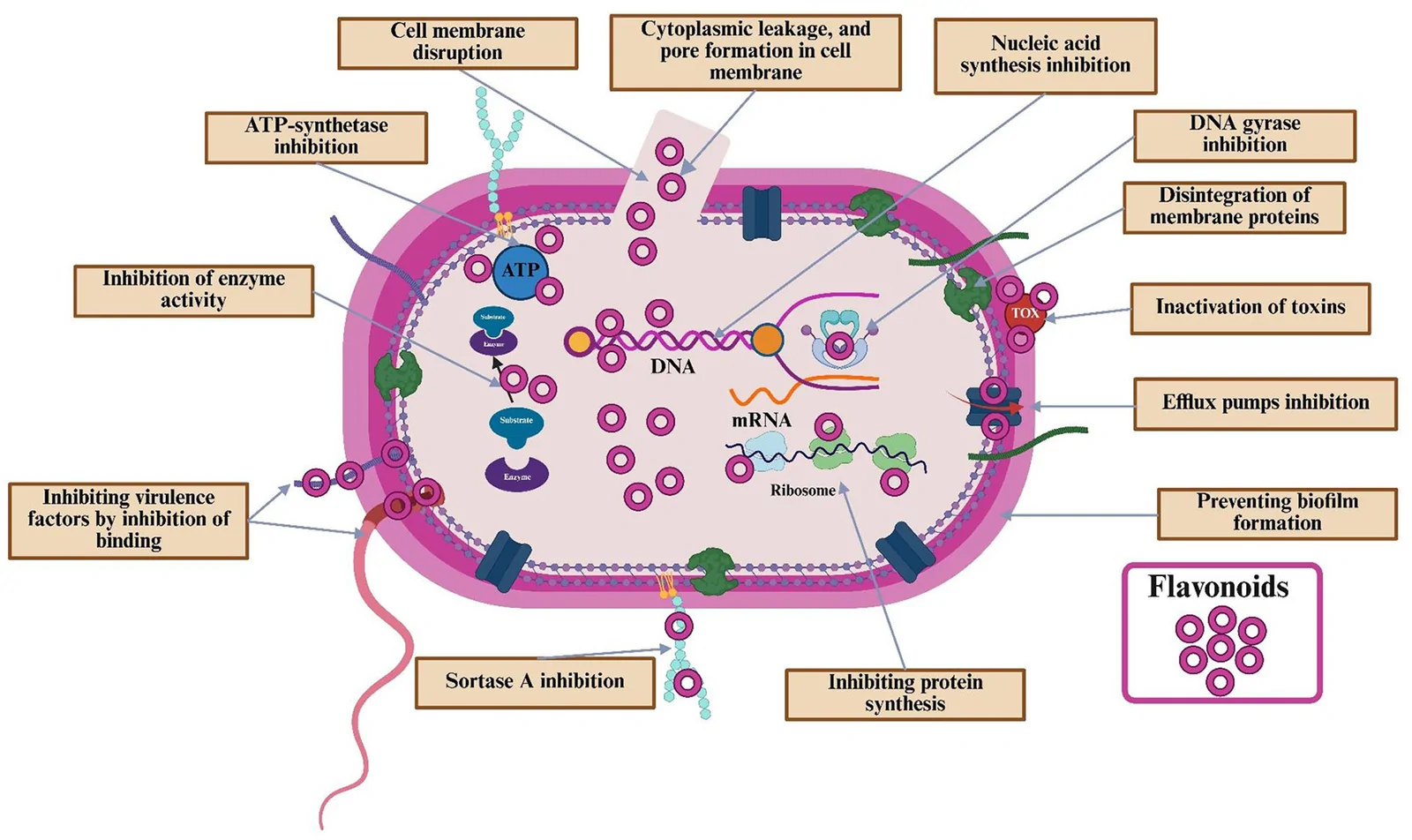
Antibacterial activity of flavonoids.

Consequently, the continued discovery and characterization of flavonoids with efficacy against drug-resistant pathogens remains a critical area of research, offering valuable prospects for the development of novel antimicrobial therapies (302). Figure 5 depicts the antibacterial activities of flavonoids, and the antibacterial action of flavonoids is demonstrated in Table 8.

**Table 8:** Antibacterial activity of flavonoids.

Subclass (with examples)	Study trial	Common natural sources	Primary antibacterial mechanisms	Observed outcomes	References
Flavanols (e.g., Epigallocatechin gallate, epicatechin, catechin, and gallocatechin)	*In vitro*	Green tea (*Camellia sinensis*), cocoa, grapes, apples, and berries.	Disrupt bacterial membranes and wall integrity. Inhibit β-lactamase and key metabolic enzymes. Interfere with peptidoglycan-membrane interactions. Inhibit quorum sensing and biofilm formation.	EGCG reduces viable counts of *Staphylococcus aureus, Streptococcus mutans*, and *Escherichia coli*. decreases biofilm biomass. Enhances β-lactam and fluoroquinolone activity against resistant strains.	(476, 477)
Anthocyanidins/Anthocyanins (e.g., Cyanidin, delphinidin, malvidin, pelargonidin, peonidin, and petunidin)	*In vitro*	Berries (blueberry, blackberry, strawberry), grapes, red cabbage, and purple sweet potato.	Damage bacterial membranes. generate oxidative stress. Inhibits extracellular polysaccharide production and quorum sensing. Chelate redox-active metals.	Reduce growth and biofilms of *Pseudomonas aeruginosa, Escherichia coli*, and oral streptococci. decrease virulence factors such as pyocyanin and elastase. exhibit anti-adhesive, anti-virulence properties.	(478, 479)
Flavanones (e.g., Hesperidin, naringenin, naringin, and eriodictyol)	*In vitro*	Citrus fruits (oranges, lemons, grapefruits), tomatoes, and oregano.	Disrupt membrane potential and energy metabolism. Inhibit cell-wall and nucleic-acid enzymes. Downregulate efflux pumps and quorum-sensing systems.	Inhibit *Staphylococcus aureus, Listeria monocytogenes*, and *Escherichia coli*. decrease motility and biofilm maturation. Naringenin restores antibiotic sensitivity in Gram-negative bacteria.	(476, 480)
Flavonols (e.g., Quercetin, kaempferol, myricetin, isorhamnetin, and fisetin)	*In vitro*	Onions, apples, kale, broccoli, berries, and tea.	Inhibit DNA gyrase and topoisomerase IV. Block fatty-acid and peptidoglycan synthesis. Disrupt membranes. inhibit efflux pumps and biofilm signaling.	Quercetin and kaempferol suppress MRSA and uropathogenic *Escherichia coli* growth. Reduce toxin and adhesion gene expression. demonstrate broad anti-virulence activity.	(481, 482)
Isoflavones (e.g., Genistein, daidzein, glycitein, biochanin A, and formononetin)	*In vitro*	Soybeans, chickpeas, red clover, and legumes.	Inhibit topoisomerases and tyrosine kinases. Impair membrane integrity. Suppress quorum-sensing virulence factors. Chelate metal cofactors.	Genistein inhibits Gram-positive cocci and biofilm formation (*Staphylococcus aureus, Enterococcus*). Reduces acid production and adherence of oral pathogens. Moderate antibacterial, strong anti-virulence potential.	(483)
Flavones (e.g., Apigenin, luteolin, baicalein, chrysin, and tangeretin)	*In vitro*	Parsley, celery, chamomile, *Scutellaria baicalensis* (Chinese skullcap), and citrus peel.	Inhibit DNA and protein synthesis. Block FtsZ polymerization. Reduce membrane fluidity. Interfere with quorum-sensing regulators and EPS biosynthesis.	Suppress growth of *Staphylococcus aureus, Bacillus subtilis*, and streptococci. Disrupt biofilm architecture. Lower secretion of hemolysins and proteases. Act as anti-virulence and bacteriostatic agents.	(484, 485)
Chalcones (e.g., Xanthohumol, licochalcone A, isoliquiritigenin, and butein)	*In vitro*	Hops (*Humulus lupulus*), licorice (*Glycyrrhiza* spp.), *Angelica keiskei* (ashitaba), and *Alpinia* species.	Disrupt bacterial membranes. Inhibit peptidoglycan and fatty-acid synthesis. Block efflux pumps and early biofilm attachment.	Xanthohumol and licochalcone A are active against MRSA and *Pseudomonas aeruginosa*. Reduce biofilm formation. restore antibiotic susceptibility. Serve as dual-action antibacterial adjuvants.	(486)
Proanthocyanidins (e.g., procyanidin B1/B2, prodelphinidin, and procyanidin C1)	*In vitro*	Cranberries, cocoa, grapes, apples, and pine bark.	Prevent bacterial adhesion to epithelial surfaces. Inhibit glycosyl transferases and fimbriae formation. Suppress quorum sensing.	Cranberry proanthocyanidins inhibit *E. coli* and *Helicobacter pylori* adhesion. Reduce urinary-tract infections and dental biofilms. Exhibit anti-adhesive rather than bactericidal effects.	(487, 488)
Dihydroflavonols (e.g., Taxifolin, aromadendrin, and dihydromyricetin)	*In vitro*	Douglas fir bark, milk thistle, citrus fruits, and grapes.	Interfere with bacterial nucleic-acid synthesis and membrane potential. Inhibit peptidoglycan enzymes. Act as antioxidant protectants in infected tissues.	Demonstrate activity against *Staphylococcus aureus* and *Escherichia coli*. reduce biofilm formation and oxidative stress responses in bacteria.	(485)
Flavonolignans (e.g., silybin, silychristin, silydianin, and isosilybin)	*In vitro*	Milk thistle (*Silybum marianum*) seeds.	Damage bacterial membranes. Inhibit ATP synthase and topoisomerases. Attenuate biofilm formation.	Show antibacterial effects against *Staphylococcus aureus, Escherichia coli*, and *Candida* biofilms. Enhance antibiotic efficacy in combined treatments.	(489, 490)
Aurones (e.g., Aureusidin, sulfuretin, 4,6-dihydroxyaurone, and 4-hydroxyaurone)	*In vitro*	Snapdragons (*Antirrhinum majus*), daisies, *Coreopsis*, and some legumes.	Inhibit bacterial DNA gyrase and FtsZ polymerization. Disrupt cell-membrane potential and integrity.	Exhibit growth-inhibitory and anti-biofilm effects against *Staphylococcus aureus, Escherichia coli*, and *Bacillus* species. Promising antibacterial scaffolds.	(491)

EGCG, epigallocatechin gallate.

### 8.7 Antiviral activity

Current research has increasingly focused on natural compounds, particularly flavonoids, for their broad-spectrum antiviral potential (303). Extensive evidence shows that flavonoids possess antiviral activity against a broad spectrum of viruses, including adenoviruses, coronaviruses, polioviruses, respiratory syncytial virus, herpesviruses, coxsackieviruses, hepatitis viruses, and dengue virus (304).

Several flavonoids have shown notable antiviral efficacy, as shown in Table 9. For instance, isoscutellarein-8-methyl ether and rutin have exhibited strong inhibitory activity against the influenza virus, with rutin also displaying antipolioviral effects. Supporting this, Wang et al. (305) reported that total flavonoids extracted from *Astragalus* produced a potent therapeutic effect in guinea pigs infected with human herpesvirus (HSV21), successfully curing the associated skin infection.

**Table 9:** Antiviral activity of flavonoids.

Subclass (with examples)	Study trial	Common natural sources	Primary antiviral mechanisms	Observed outcomes	References
Flavanols (e.g., Epigallocatechin gallate, epicatechin, catechin, and gallocatechin)	*In vitro*	Green tea (*Camellia sinensis*), cocoa, grapes, apples, and berries.	Block viral entry by binding to surface glycoproteins and host receptors. Inhibit viral enzymes (reverse transcriptase, RNA polymerase, proteases). Disrupt viral envelopes and replication.	EGCG inhibits >90% replication of influenza, hepatitis B, HSV, and SARS-CoV-2. Reduces viral load and cytopathic effects. Broad-spectrum action confirmed across DNA and RNA viruses.	(492, 493)
Anthocyanidins/Anthocyanins (e.g., Cyanidin, delphinidin, malvidin, pelargonidin, peonidin, and petunidin)	*In vitro*	Berries (blueberry, blackberry, strawberry), grapes, red cabbage, and purple sweet potato.	Inhibit viral adsorption and penetration. Block neuraminidase and viral polymerase. Modulate immune responses via antioxidant and anti-inflammatory effects.	Decrease viral titers in influenza and RSV. Reduce tissue inflammation and oxidative damage. Anthocyanin-rich diets are linked to lower incidence and severity of respiratory infections.	(494)
Flavanones (e.g., Hesperidin, naringenin, naringin, and eriodictyol)	*In vitro*	Citrus fruits (oranges, lemons, grapefruits), tomatoes, and oregano.	Bind to viral surface proteins and host ACE2 receptors. Inhibit viral proteases and polymerases. Suppress cytokine storms and inflammation.	Hesperidin and naringenin inhibit replication of influenza, dengue, Zika, and SARS-CoV-2. Lower viral RNA levels and improve survival. Docking and biological studies confirm strong enzyme binding.	(495, 496)
Flavonols (e.g., Quercetin, kaempferol, myricetin, isorhamnetin, and fisetin)	*In vitro*	Onions, apples, kale, broccoli, berries, and tea.	Inhibit reverse transcriptase, integrase, and proteases. Block spike–receptor binding. Interfere with viral replication and protein maturation.	Quercetin and kaempferol reduce replication of HSV, dengue, and SARS-CoV-2. Quercetin enhances recovery from respiratory infections, especially with vitamin C or zinc.	(497, 498)
Isoflavones (e.g., Genistein, daidzein, glycitein, biochanin A, and formononetin)	*In vitro*	Soybeans, chickpeas, red clover, and legumes.	Inhibit viral tyrosine kinases and gene transcription. Enhance interferon signaling and antiviral cytokine release.	Genistein inhibits the replication of herpesviruses, enteroviruses, and HIV. Functions as a host-directed antiviral with broad activity and low cytotoxicity.	(499)
Flavones (e.g., Apigenin, luteolin, baicalein, chrysin, and tangeretin)	*In vitro*	Parsley, celery, chamomile, *Scutellaria baicalensis* (Chinese skullcap), and citrus peel.	Bind and inhibit viral proteases, polymerases, and helicases. Limit viral entry. Suppress pro-inflammatory cytokine storms.	Luteolin and baicalein strongly inhibit dengue, influenza, and SARS-CoV-2. Reduce viral RNA and protein levels. Inhibit SARS-CoV-2 3CL protease and modulate host immunity.	(500, 501)
Chalcones (e.g., Xanthohumol, licochalcone A, isoliquiritigenin, and butein)	*In vitro*	Hops (*Humulus lupulus*), licorice (*Glycyrrhiza* spp.), *Angelica keiskei* (ashitaba), and *Alpinia* species.	Inhibit viral protease and nucleocapsid assembly. Block replication and packaging. Activate interferon-stimulated antiviral genes.	Xanthohumol and related chalcones inhibit dengue, hepatitis C, HIV, and SARS-CoV-2 replication. Suppress 3CL protease. Lower viral loads and inflammation.	(502, 503)
Proanthocyanidins (e.g., Procyanidin B1/B2, prodelphinidin, and procyanidin C1)	*In vitro*	Cranberries, cocoa, grapes, apples, and pine bark.	Block viral attachment and fusion. bind viral surface proteins. Inhibit neuraminidase and protease enzymes.	Cranberry and grape proanthocyanidins reduce influenza and rotavirus infection rates. They show broad inhibition of viral adhesion and propagation.	(504, 505)
Dihydroflavonols (e.g., Taxifolin, aromadendrin, and dihydromyricetin)	*In vitro*	Douglas fir bark, milk thistle, citrus fruits, and grapes.	Inhibit viral polymerases and helicases. Suppress replication and ROS-driven viral propagation.	Taxifolin reduces influenza and hepatitis C viral replication *in vitro*. Decreases viral-induced oxidative stress and inflammatory signaling.	(492, 506)
Flavonolignans (e.g., Silybin, silychristin, silydianin, and isosilybin)	*In vitro*	Milk thistle (*Silybum marianum*) seeds.	Block viral RNA polymerase and NS5B enzyme activity. Inhibit entry and replication of enveloped viruses.	Silymarin and silibinin inhibit HCV, HBV, and dengue replication. Lower viral RNA and improve hepatic function in infected models and clinical settings.	(507)
Aurones (e.g., Aureusidin, sulfuretin, 4,6-dihydroxyaurone, and 4-hydroxyaurone)	*In vitro*	Snapdragons (*Antirrhinum majus*), daisies, *Coreopsis*, and some legumes.	Inhibit viral polymerases and proteases. Block genome replication and assembly.	Show antiviral activity against HSV, influenza, and enteroviruses. Suppress viral replication and promote host antiviral responses *in vitro*.	(508)

EGCG, epigallocatechin gallate.

Studies investigating total flavonoids extracted from *Viola kunawarensis* var. TFVK have shown noticeable anti-influenza virus effects in both *in vitro* and *in vivo* models. These studies, using Western blot and cytopathic effect (CPE) assays, suggest that the main antiviral mechanism likely involves direct inhibition of viral M2 and NS1 protein expression (306).

Additionally, by inhibiting the expression and nuclear translocation of the host cell's p65 protein, TFVK may interrupt the NF-κB signaling cascade and inhibit the synthesis of pro-inflammatory proteins, thereby exerting indirect antiviral effects. Collectively, these findings underscore the therapeutic promise of flavonoids as multifunctional antiviral agents, capable of targeting both viral replication and host inflammatory responses (306).

In the search for effective treatments against SARS-CoV-2, a high-throughput screening of 1,019 flavonoid compounds identified apigenin and a galloylated pinocembrin derivative (PGHG) as the most potent inhibitors of viral replication, highlighting their potential as promising lead molecules for antiviral drug development (307).

In a separate study, the flavonoids sakuranetin (SEK) and velutin (VEL), isolated from *Rhus retinorrhoea*, demonstrated notable inhibitory activity against the hepatitis B virus (HBV). At a concentration of 12.5 μg mL^−1^, SEK and VEL reduced HBsAg levels by approximately 58.8% and 56.4%, respectively, and HBeAg levels by 55.5% and 52.4%. Molecular docking analyses further substantiated these results, revealing that both flavonoids form stable complexes with HBV polymerase and capsid proteins, suggesting a structure-based inhibitory mechanism underlying their antiviral effects (308).

Moreover, the flavonoid luteolin has demonstrated broad-spectrum antiviral activity against a variety of pathogens, including coronaviruses, influenza viruses, and enteroviruses. The modulation of several key signaling pathways—such as PI3K-AKT, TLR4/8, NF-κB, Nrf2/HO-1, and MAPK—is central to its mechanism of action in controlling viral infection and replication (309).

Additionally, luteolin, with its direct antiviral effects, enhances the body's antioxidant defenses and non-specific immune responses, contributing to improved resistance against viral pathogens. Its antiviral efficacy is further mediated through the regulation of critical cellular receptors and cytokines, including p53, NLRP3, TNF-α, HNF-4α, and various interleukins, collectively leading to the suppression of intracellular viral replication and attenuation of virus-induced inflammation (309). The antiviral activity of flavonoids is demonstrated in Table 9.

## 9 Therapeutic effects of flavonoids

### 9.1 Alcoholic fatty liver and non-alcoholic fatty liver

The rising global prevalence of non-alcoholic fatty liver disease (NAFLD) and alcoholic fatty liver disease (AFLD) has intensified efforts to identify effective therapeutic agents. In the USA, the flavonoid dihydromyricetin is marketed as a dietary supplement for hangover prevention (310); however, emerging research suggests it has much broader therapeutic potential, extending beyond its traditional use.

Studies conducted in mouse models of alcohol-related liver disease (ALD) have shown that intraperitoneal administration of dihydromyricetin (5–10 mg kg^−1^) significantly upregulates the expression of key ethanol-metabolizing enzymes. This treatment concurrently reduces serum levels of inflammatory cytokines and chemokines, indicating both metabolic and anti-inflammatory benefits in mitigating alcohol-induced hepatic injury (311).

Other flavonoids, including baicalein and vitexin, have demonstrated protective effects in alcohol-related liver disease (ALD) rodent models. Baicalein has been reported to alleviate ethanol-induced hepatic injury and strengthen antioxidant defenses (312), whereas vitexin notably reversed alcohol-induced alterations in key serum biochemical markers, including AST, ALT, total cholesterol (TC), triglycerides (TG), and total bilirubin (TBIL). Moreover, vitexin improved liver histology and metabolic function, underscoring its potential as a multifunctional hepatoprotective agent (313).

In contrast, no pharmaceutical agent has yet been approved for the treatment of NAFLD. Cao et al. (314) examined the effectiveness of quercetin in NAFLD using both *in vitro* and *in vivo* models. The study utilized specific pharmacological inhibitors targeting distinct phases of autophagy: 3-methyladenine (3-MA) for initiation and chloroquine (CQ) for degradation, alongside inhibitors of critical regulatory molecules, compound C for AMP-activated protein kinase (AMPK), and EX-527 for sirtuin 1 (SIRT1). The findings indicated that quercetin mitigates NAFLD primarily by activating the AMPK pathway, thereby facilitating mitophagy and improving hepatic cellular homeostasis (314).

Wogonin exhibits a dual protective role against both liver fibrosis and hepatocellular carcinoma (HCC). It mitigates fibrosis by inhibiting hepatic stellate cell (HSC) activation and inducing HSC apoptosis. This effect is mediated through the enhanced activity of caspase-9 and caspase-3, coupled with an increased Bax/Bcl-2 ratio, indicating activation of the intrinsic apoptotic pathway and highlighting wogonin's potential as a therapeutic agent for chronic liver diseases (315).

In liver cancer, wogonin inhibits the proliferation of carcinoma cells, induces cell cycle arrest, and modulates key metabolic pathways by directly targeting glycogen synthase kinase-3β (GSK-3β), thereby contributing to its anticancer and metabolic regulatory effects (316). Separately, oroxylin A provides hepatoprotective effects by reducing hepatocyte pyroptosis through the suppression of caspase-1 activation within the NLRP3 inflammasome pathway, thereby mitigating inflammatory cell death and protecting liver tissue integrity (317).

Quercetin alleviates AFLD through multiple interconnected mechanisms. It suppresses ethanol-induced overexpression of perilipin 2 (PLIN2), promotes lipophagy by enhancing autophagic lysosome formation, and restores mitochondrial integrity, collectively contributing to improved lipid metabolism and hepatocellular function (318).

Furthermore, quercetin counteracts alcohol-induced lipid accumulation, inflammation, and oxidative stress by blocking the purinergic P2X7 receptor (P2X7R), thereby inhibiting NLRP3 inflammasome activation. This mechanism effectively reduces inflammatory cytokine release and attenuates hepatic injury associated with chronic alcohol exposure (319).

The hesperidin derivative 4-MCH mitigates alcohol-induced hepatic inflammation by significantly reducing interleukin-6 (IL-6) and tumor necrosis factor-alpha (TNF-α) release, suppressing NF-κB activation, and upregulating peroxisome proliferator-activated receptor gamma (PPARγ) expression in murine macrophages, thereby exerting potent anti-inflammatory and hepatoprotective effects (320).

**Figure 6:**
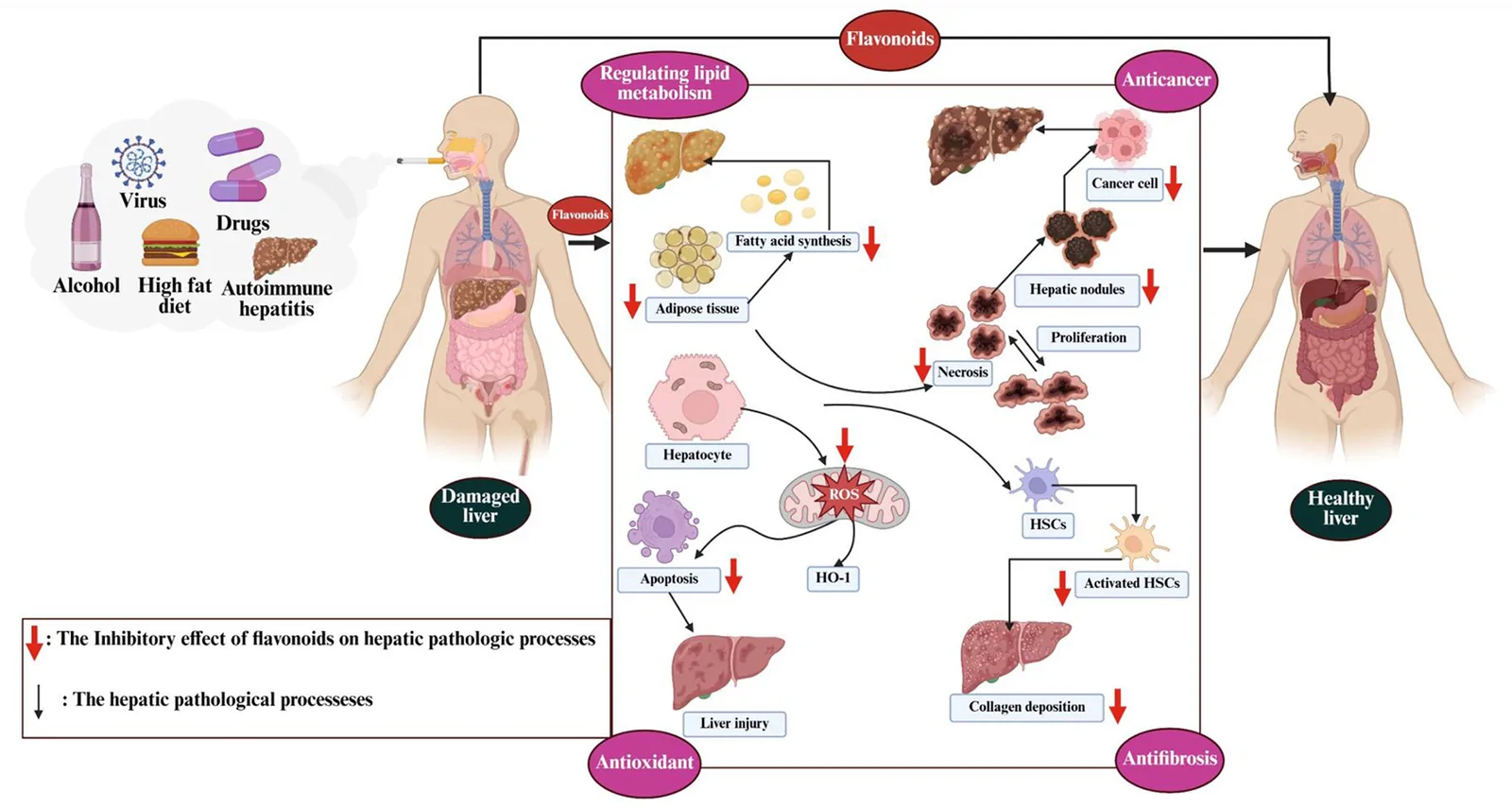
Therapeutic role of flavonoids on liver diseases.

Finally, puerarin ameliorates alcohol-induced hepatic steatosis by regulating the AMP-activated protein kinase alpha/acetyl-CoA carboxylase (AMPKα/ACC) signaling pathway, thereby restoring lipid homeostasis and preventing excessive fat accumulation in the liver (321) and this agent reduces chronic alcoholic liver injury in rats by modulating the COX-2 and arachidonate 5-lipoxygenase (ALOX5) pathways, contributing to improved liver health and reduced inflammation (322). The therapeutic effect that flavonoids have in the treatment of liver disorders is illustrated in Figure 6.

### 9.2 Pulmonary fibrosis

Pulmonary fibrosis is a progressive and chronic interstitial lung disease with diverse etiologies, characterized by pronounced pathological heterogeneity, high mortality, and the extensive disruption of normal lung tissue architecture. Although pirfenidone and nintedanib have been approved as current therapeutic options, the development of more effective pharmacological interventions remains an urgent and unmet clinical necessity (323).

The adenosine A2a receptor (A2aR) has recently been identified as a key regulator of inflammatory and fibrotic signaling pathways. The flavonoid baicalin exhibits potent antifibrotic activity through its interaction with this receptor (324). Mechanistically, A2aR activation mitigates bleomycin (BLM)-induced pulmonary fibrosis by inhibiting the activation of transforming growth factor β1 (TGF-β1) and downregulating extracellular signal-regulated kinases (ERK1/2), thereby suppressing fibroblast proliferation and extracellular matrix deposition (324).

In a related finding, baicalein may inhibit fibrosis by suppressing fibroblast proliferation. This antifibrotic effect occurs through induction of G0/G1 cell-cycle arrest, downregulation of cyclins A, D, and E, and increased intracellular calcium (Ca^2+^) concentration, collectively leading to reduced fibroblast activity and attenuation of fibrotic progression (325). An alternative mechanism proposed for baicalin involves inhibiting the transforming growth factor β1 (TGF-β1)/Smad signaling pathway by upregulating Sirtuin 3 (Sirt3) *in vivo*, thereby attenuating fibroblast activation and collagen deposition and enhancing its antifibrotic efficacy (326).

Morin, a flavonoid derived from Moraceae plants, exerts strong antifibrotic activity by suppressing the differentiation of fibroblasts into myofibroblasts and ameliorating bleomycin (BLM)-induced pathological lung damage in mouse models. Furthermore, it has been shown to inhibit the activation of NIH-3T3 fibroblast cells stimulated by transforming growth factor β1 (TGF-β1), highlighting its potential as a therapeutic candidate for pulmonary fibrosis (327).

Similarly, silymarin exerts a significant protective effect against pulmonary fibrosis by reducing hydroxyproline content and collagen fiber deposition in lung tissue. It also modulates key molecular markers, leading to decreased levels of transforming growth factor β1 (TGF-β1) and fibronectin, while increasing the expression of matrix metallopeptidase 2 (MMP-2) and interferon-gamma (IFN-γ). These combined actions contribute to the attenuation of fibrotic remodeling and the restoration of pulmonary tissue integrity (328).

In mice, bleomycin (BLM)-induced inflammation and collagen deposition are reduced due to calycosin's activation of the Nrf2/HO-1 signaling pathway, its promotion of apoptosis, and its potent antifibrotic and anti-inflammatory effects (329). A proposed mechanism involves the upregulation of autophagy-related proteins, including LC3, beclin1, and PINK1, accompanied by a decrease in p62 levels, indicating enhanced autophagic flux. Furthermore, calycosin prevents the release of degradative lysosomal enzymes and shields pulmonary tissue from progressive fibrotic damage by maintaining autophagy through upregulation of transcription factor EB (TFEB) and lysosome-associated membrane protein 1 (LAMP1) (329).

Another study suggests that calycosin mitigates pulmonary fibrosis by modulating the microRNA-375 (miR-375)/Yes-associated protein 1 (YAP1) signaling pathway, thereby suppressing transforming growth factor β1 (TGF-β1)-induced epithelial-mesenchymal transition (EMT). This regulatory mechanism helps preserve epithelial integrity, inhibit fibroblast activation, and attenuate extracellular matrix accumulation, thereby contributing to the overall antifibrotic effect of calycosin (330). Furthermore, ginkgetin, isolated from *G. biloba*, alleviates oxidative stress and lung fibrosis primarily via an AMPK-dependent signaling mechanism (331).

There is increasing recognition of the critical pathogenic role of mitochondrial dysfunction in the development and progression of pulmonary fibrosis, although the underlying molecular mechanisms remain incompletely understood. Therefore, future research should focus on elucidating how flavonoids modulate mitochondrial function, as these insights may uncover novel therapeutic strategies to mitigate fibrotic lung disease and restore cellular energy homeostasis.

### 9.3 Cardiovascular diseases

Flavonoids hold significant promise as both nutraceutical and pharmaceutical agents for enhancing endothelial function and managing cardiovascular diseases. Their multifaceted biological activities, including antioxidant, anti-inflammatory, vasodilatory, and lipid-regulating effects, make them valuable candidates for preventing and mitigating vascular dysfunction and associated cardiovascular complications (332).

Therefore, maintaining a diet rich in flavonoids is strongly recommended to reduce cardiovascular risk, as these compounds improve endothelial function, regulate blood pressure, inhibit low-density lipoprotein cholesterol (LDL) oxidation, and enhance vascular health, collectively contributing to the prevention of cardiovascular diseases (333).

The mechanisms underlying the cardioprotective effects of flavonoids are multifaceted. For instance, in an *in vitro* study, during ROS scavenging, rutin undergoes oxidation to form quinone derivatives. These metabolites subsequently activate the Nrf2 antioxidant signaling pathway, enhancing the expression of cytoprotective enzymes and providing localized defense in regions of elevated oxidative stress. Through this mechanism, rutin contributes to vascular protection and reduces overall cardiovascular disease risk (334).

Similarly, hesperidin exerts both antioxidant and anti-inflammatory effects, which are associated with reduced serum triglyceride (TG) levels and slowed progression of atherosclerosis in patients with cardiovascular diseases following coronary artery bypass grafting (CABG). These actions contribute to improved vascular health and enhanced post-surgical cardiovascular outcomes (335).

Studies in animal models demonstrate that chrysin mitigates isoproterenol-induced heart failure, hemodynamic dysfunction, and structural damage to cardiac tissue, in addition to reducing fibrosis and collagen deposition (336). Moreover, genistein inhibits crucial pro-atherogenic processes by suppressing the secretion and gene expression of adhesion molecules and chemokines in endothelial cells exposed to oxidized low-density lipoprotein (oxLDL). In addition, it counteracts angiotensin-induced atherosclerotic damage, thereby contributing to the preservation of endothelial integrity and the prevention of vascular inflammation and plaque formation (337).

### 9.4 Other biological effects

In the field of natural product drug development, flavonoids constitute a major class of bioactive compounds with substantial potential for therapeutic discovery, particularly for addressing serious, multifactorial diseases. Alzheimer's disease (AD), a progressive neurodegenerative disorder characterized by gradual cognitive decline and an incompletely understood etiology, remains a major global health challenge. To explore the neuroprotective and anti-inflammatory roles of flavonoids in Alzheimer's pathology, the pharmacological activities and underlying mechanisms of 13 plant-derived flavonoids, including apigenin, luteolin, naringin, and quercetin, were investigated by Zhang and Yan (338). Their research aimed to elucidate how these compounds modulate Alzheimer's-related neuroinflammation and contribute to the mitigation of neuronal damage.

The molecular mechanisms by which polymethoxyflavones (PMFs) alleviate various chronic diseases were particularly highlighted in a comprehensive review by Gan et al. (339), who examined the biological roles, metabolism, biosynthesis, extraction methods, and sources of PMFs, a distinctive dietary flavonoid found in plants, including *Citrus reticulata*.

Some flavonoids exhibit significant neuroprotective characteristics. For instance, kaempferol and its derivatives provide protection via various mechanisms, including antioxidant properties, maintenance of blood–brain barrier integrity, and inhibition of amyloidogenic fibril formation involving critical proteins such as amyloid-β (Aβ), tau (τ), and α-synuclein. In neurodegenerative disorders such as Alzheimer's disease, mitigating neuroinflammation and preserving neuronal viability can be achieved by inhibiting microglial activation, reducing pro-inflammatory cytokine release, and restoring mitochondrial membrane potential (340).

Quercetin has similarly been found to reduce depression-like behaviors induced by corticosterone, an effect partly linked to decreased neuroinflammation and oxidative stress (341). Proanthocyanidins, known for their strong antioxidant and anti-inflammatory properties, have been shown to alleviate symptoms of skin allergies and allergic asthma. Their therapeutic action involves inhibiting the synthesis and release of inflammatory mediators, including histamine, serotonin, prostaglandins, and leukotrienes, and preventing the degranulation of basophils and mast cells. Through these mechanisms, proanthocyanidins help reduce allergic inflammation and stabilize immune responses in hypersensitivity conditions (342).

These compounds may also alleviate gastrointestinal disorders and arthritis by inhibiting enzymes such as hyaluronidase and histidine decarboxylase (343). Furthermore, proanthocyanidins have been shown to possess notable anti-radiation capabilities (344). Some flavonoids also exhibit hormone-regulating functions. Isoflavonoids derived from whole grains, nuts, soybeans, and flaxseeds act as selective estrogen receptor modulators (SERMs), displaying estrogen-antagonistic effects in high-estrogen environments and agonistic activity under low-estrogen conditions (345).

Flavonoids also influence depressive disorders through the gut–brain axis. By modulating the composition of gut microbiota, they can significantly alleviate depressive symptoms, a mechanism of particular relevance given the well-documented alterations in gut microbial profiles associated with depression (346).

**Figure 7:**
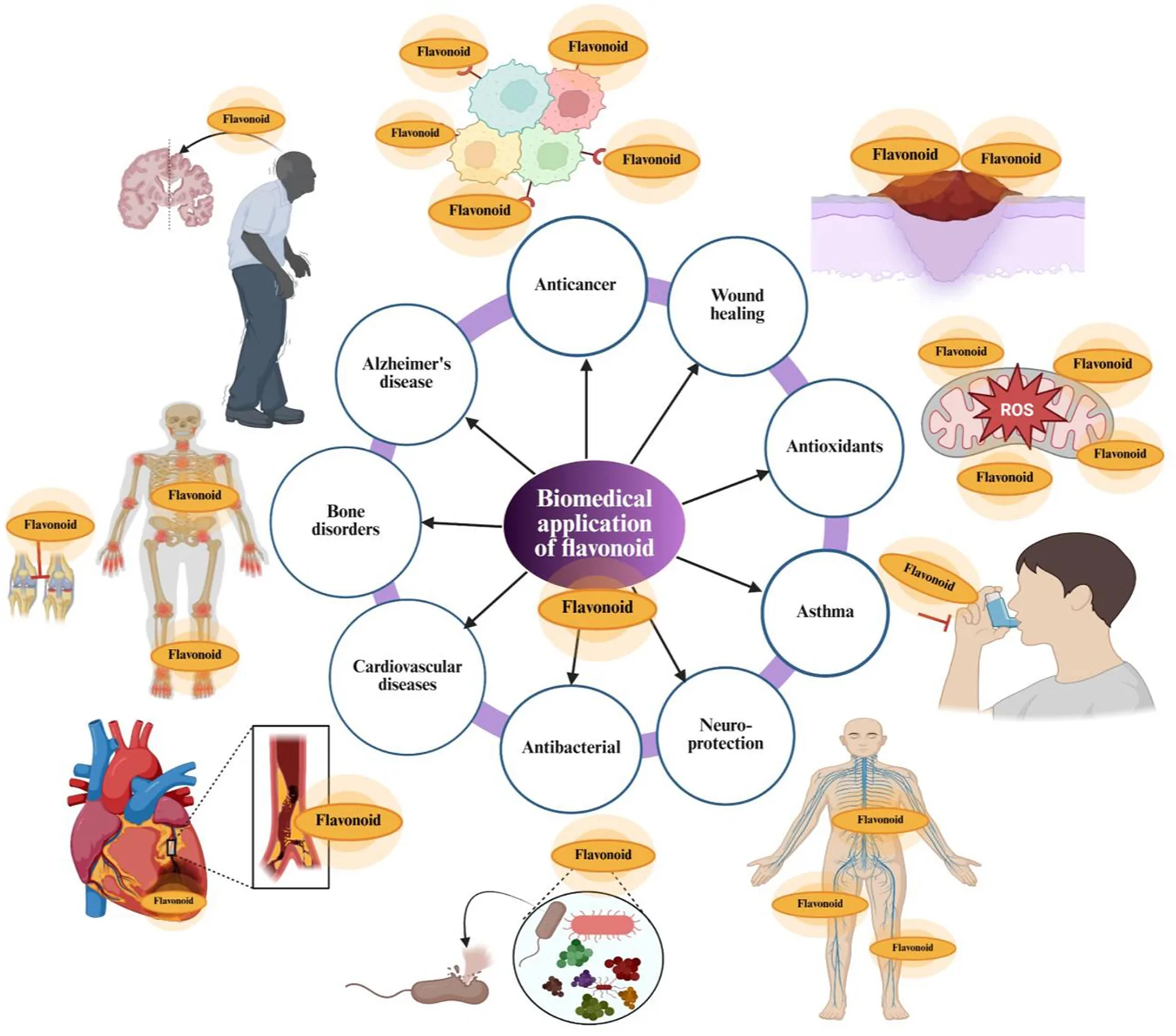
Different therapeutic effects of flavonoids.

Beyond these neurological and metabolic functions, specific flavonoids like licoflavone B, obtained from *Glycyrrhiza inflata*, exhibit targeted anti-parasitic activity, demonstrating IC_50_ values of 23.78 μM and 31.50 μM against *Schistosoma mansoni* ATPase and ADPase, respectively (347). Therefore, as new health challenges continue to emerge, research interest in the pharmacological functions of flavonoids is expected to increase correspondingly, reflecting their diverse therapeutic potential and broad biomedical relevance. Figure 7 illustrates numerous therapeutic benefits of flavonoids.

**Figure 8:**
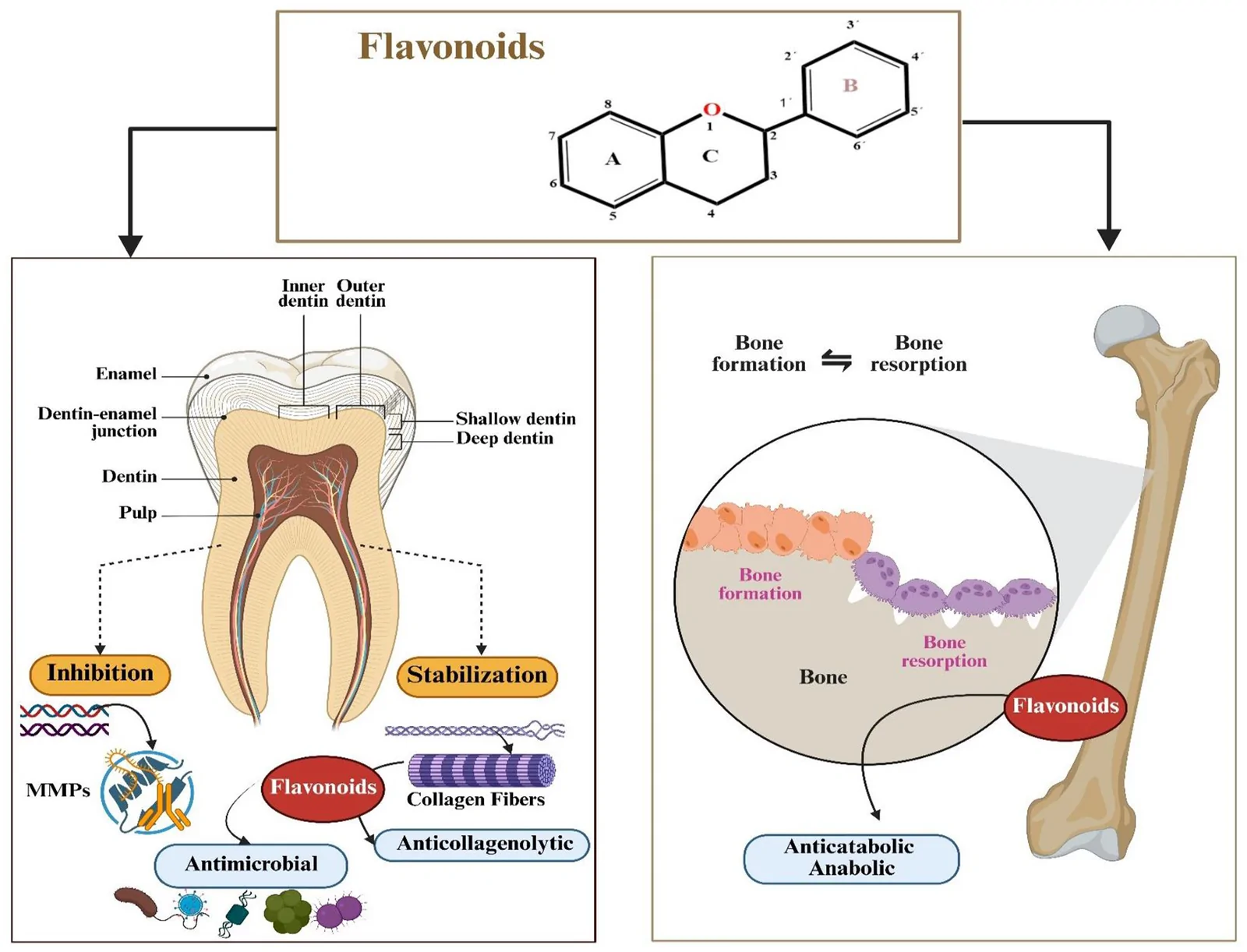
Biological effects of flavonoids on bone formation and dental tissue (the dentin matrix). MMP, matrix metalloproteinase.

Flavonoids are increasingly recognized for their potential to positively influence bone remodeling—the continuous process of bone breakdown (resorption) and formation. They exert a protective effect by addressing the imbalance characteristic of conditions such as osteoporosis, in which excessive osteoclast-mediated bone resorption outpaces osteoblast-mediated bone formation. Mechanistically, various flavonoids have been shown to inhibit osteoclast differentiation and activity, thereby suppressing the rate of bone resorption. Simultaneously, many flavonoids, particularly isoflavones such as genistein, which structurally mimic estrogen, have been reported to promote osteoblast proliferation and differentiation, thereby favoring new bone formation. Figure 8 depicts the biological impacts of flavonoids on bone development and dental tissue, namely the dentin matrix.

## 10 Applications of flavonoids in food and pharmaceutical products

### 10.1 Food processing

According to Ren et al. (348), different food processing techniques can markedly influence the biological activity of flavonoids. Thermal methods such as boiling and baking, along with enzymatic treatments like enzymolysis, can add or remove specific chemical groups, thereby modifying the fundamental structures and potentially altering the bioavailability and functionality of these compounds (349).

Such structural modifications can enhance the functional properties of flavonoids, thereby increasing antioxidant, anti-inflammatory, and anticancer activities (350). A notable example arising from thermal processing is the formation of styryl flavonoids in meats and fortified foods, which exhibit strong disease-preventive potential. For instance, 6-C-(E-phenylethenyl)-naringenin was shown to increase total SOD activity by 63.3% in aged human umbilical vein endothelial cells (HUVECs), a result that far exceeded the 7.2% increase observed with naringenin (351).

Structural modifications can also improve food stability and functional properties. For example, epicatechin has been shown to inhibit starch retrogradation in chestnut more effectively than either epicatechin gallate or oligomeric proanthocyanidins, highlighting the impact of molecular structure on food functionality and preservation (352).

Moreover, heat treatment facilitates interactions between flavonoids and macromolecules such as proteins and polysaccharides. The resulting complexes not only enhance the nutritional value of foods but also improve sensory characteristics, including color, flavor, and overall palatability (352, 353).

### 10.2 Beverage industry

In the beverage industry, flavonoids function as powerful natural antioxidants and antimicrobial agents. They extend shelf life and enhance product preservation by preventing lipid oxidation, protecting essential vitamins and enzymes, and inhibiting microbial growth, thereby maintaining both the quality and nutritional integrity of beverages (354, 355). Their ability to scavenge hypochlorous acid (HOCl) is closely influenced by their molecular structure. The presence of hydroxyl groups on the A- and B-rings enhances chlorination reactivity, whereas the C2=C3 double bond reduces this activity, reflecting a delicate balance between structural features and antioxidant function (356).

Flavonoids, such as rutin, can also enhance the color profiles of beverages such as wine by increasing the concentration of anthocyanins and related pigments, resulting in a richer and more intense red hue that improves both visual appeal and perceived quality (357, 358). Furthermore, the number and spatial arrangement of hydroxyl and methoxy groups in flavonoid molecules influence their charge distribution and functional behavior in multiphase food systems. In citrus-based products, flavonoids play a key role in reducing turbidity in juices and jams, thereby enhancing clarity, flavor, and overall quality by influencing antioxidant activity, sedimentation, and particle dynamics. Structural modifications can further optimize these properties (359).

For instance, enzymatic conversion of bitter flavonoids into non-bitter dihydrochalcones effectively eliminates undesirable tastes in orange juice, while enzymatic deglycosylation has been shown to increase the antioxidant capacity of both oranges and orange juice, improving their nutritional and sensory value (359).

### 10.3 Food additives

Certain dihydrochalcones (DHCs), including phlorizin, phloretin, trilobatin, and neohesperidin dihydrochalcone (NHDC) are natural constituents of sweet tea (*Lithocarpus polystachyus* Rehd.) and citrus fruits. These compounds exhibit remarkable therapeutic potential for diabetes management by regulating blood glucose levels, modulating lipid metabolism, and mitigating oxidative stress and inflammatory responses, thereby improving metabolic balance and insulin sensitivity (360, 361).

As non-classical flavonoids, dihydrochalcones (DHCs) also function as potent natural sweeteners and flavor modifiers in food applications. The hydrogenation of the C2–C3 double bond increases molecular flexibility, thereby enabling stronger, more stable binding to sweet taste receptors. In neohesperidin dihydrochalcone (NHDC), specific hydrogen bonds formed by the C′2–OH and C′6–OH groups, along with hydrophobic interactions involving the C4–methoxy moiety and receptor side chains, play crucial roles in sweetness perception and receptor affinity (362).

Despite the growing market demand, the natural extraction of chalcones and DHCs remains costly and inefficient. Chalcones, which serve as key intermediates in flavonoid biosynthesis, possess structurally simple yet highly versatile chemical scaffolds, making them particularly valuable for pharmaceutical design and drug development. A wide range of synthetic and naturally occurring chalcone derivatives have demonstrated strong anticancer potential. For instance, licochalcone A, isolated from licorice root (*Glycyrrhiza glabra*), exhibits anticancer, anti-inflammatory, and antibacterial activities, underscoring the therapeutic versatility of this class of compounds (179, 363).

Tamoxifen, a chalcone-based therapeutic, functions as a selective estrogen receptor modulator and is used to treat breast and ovarian cancers (364). Similarly, the synthetic flavonoid flavopiridol acts as a broad-spectrum cyclin-dependent kinase (CDK) inhibitor. It competes with ATP to arrest the cell cycle, thereby exerting anticancer effects (365).

### 10.4 Packaging and cosmetics

Owing to their unique chemical architecture, flavonoids are increasingly being used in the packaging and preservation of both food and pharmaceutical products. A key characteristic of these compounds is the abundance of hydroxyl groups within their structures. These groups serve as crucial sites for extensive hydrogen bonding and participation in various chemical reactions (366). This attribute allows flavonoids to form strong hydrogen or covalent bonds with other molecules or polymer matrices, ensuring their stable and durable incorporation into thin films and composite materials. Consequently, flavonoids are effectively employed across a wide range of applications, including food packaging, medical devices, and sanitary products (366, 367).

Illustratively, a food-grade emulsion produced through the covalent cross-linking of soluble krill protein with rutin exhibits superior physical properties and greater resistance to oxidative degradation (368). Similarly, a coating composed of carboxymethyl chitosan and quercetin has been successfully applied to fresh-cut apples, enhancing fruit firmness, inhibiting browning, prolonging shelf life, and preserving overall quality (369).

The excellent biocompatibility and favorable safety of flavonoids further establish them as promising candidates for chemical modification in biomedical applications. For instance, a biodegradable poly(ester polyurethane) incorporating quercetin and phosphocholine exhibits enhanced blood compatibility and antimicrobial activity, making it well suited for the fabrication of durable medical devices intended for blood-contact applications (145).

Beyond their applications in packaging and biomedicine, flavonoids are also distinguished by their capacity to modulate various biological and enzymatic pathways. One of their principal enzymatic targets is tyrosinase, a key enzyme involved in melanin biosynthesis. In food systems, tyrosinase activity induces enzymatic browning, leading to undesirable changes in flavor, color, and nutritional value, and potentially forming toxic oxidative by-products. In human skin, the same enzyme catalyzes melanin formation, and its excessive activity is linked to hyperpigmentation disorders such as melasma, freckles, and age spots (370).

Numerous flavonoids, including dihydromorin from *Artocarpus heterophyllus* and quercetin-3,4′-O-diglucoside from red onion, have been identified as potent tyrosinase inhibitors (354, 355). This inhibitory capacity is strongly influenced by molecular structure; modifications such as methylation, glycosylation, and saturation of the C2=C3 bond typically reduce activity, whereas hydroxylation can modulate a compound's affinity and inhibitory potency (355).

Flavonoids also offer protection against skin damage and aging induced by ultraviolet radiation, underscoring their value as ingredients in sunscreens and cosmetic formulations (371). The bioactivity of flavonoids extends into several other vital domains. Numerous botanical medicines formulated to support women's health contain flavonoids exhibiting estrogen-like properties. Certain structural features, such as the presence of an isopentenyl group at the C8 position, are known to enhance estrogenic activity, while the C2–C3 double bond contributes to greater potency and selectivity toward estrogen receptors (372).

Furthermore, the hydroxyl groups in flavonoids exhibit a strong affinity for certain metal ions, enabling them to adsorb and remove hazardous environmental pollutants, including heavy metals. For example, naringin is capable of chelating lead ions, thereby facilitating water purification through the reduction of lead concentrations (373, 374).

**Figure 9:**
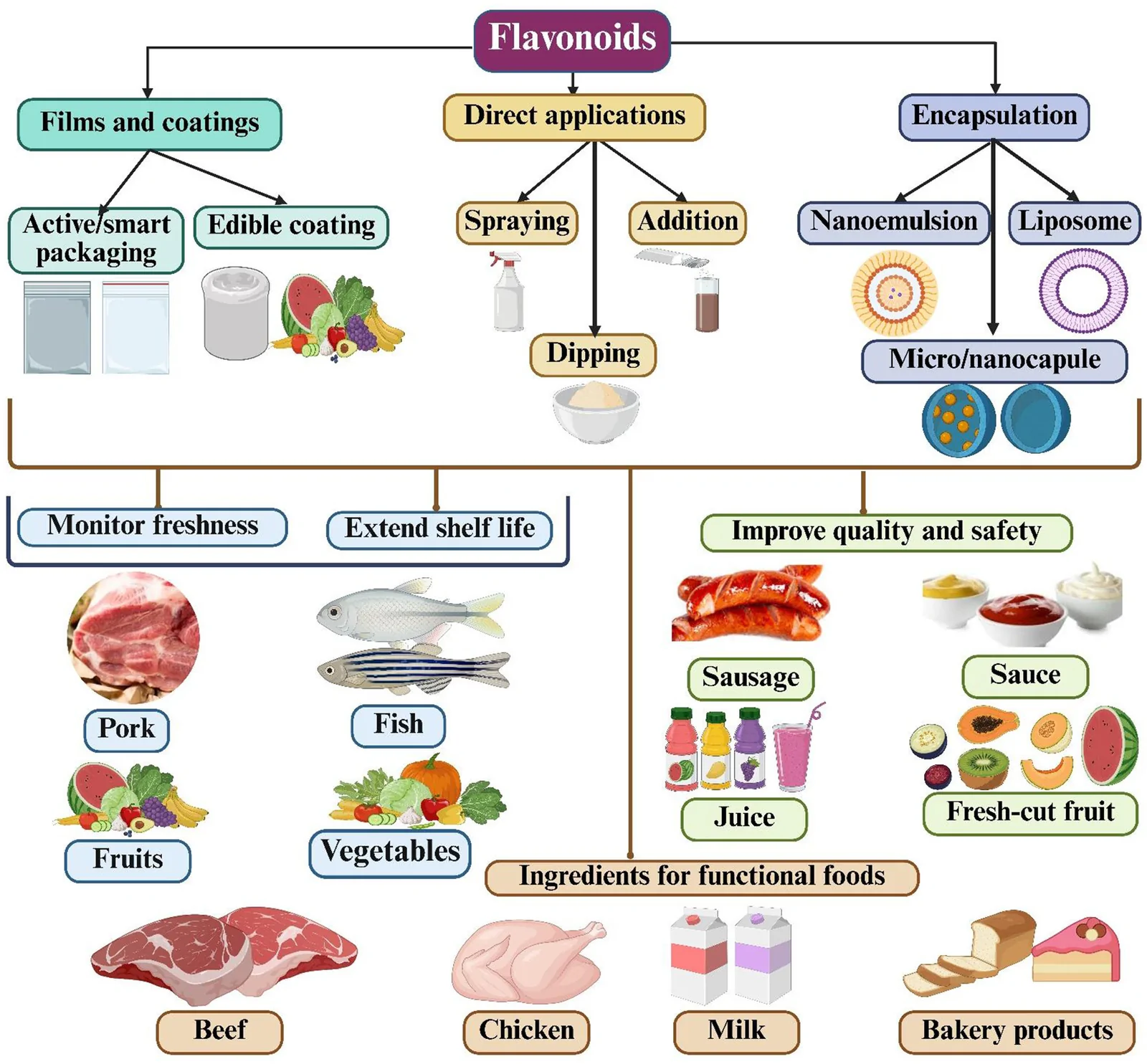
Applications of flavonoids in food processing.

Recent studies have also demonstrated that fluorescent products derived from anthocyanins can be synthesized using azobis(2-amidinopropane) dihydrochloride, offering a novel approach for detecting oxidative processes in both food science and biomedical research (375). Figure 9 highlights the applications of flavonoids in food processing.

### 10.5 Other applications

Flavonoids perform crucial, specialized physiological functions in plants, significantly strengthening their defense mechanisms and reproductive success. They enhance resistance to pests and pathogens, promote fertility, and offer protection against ultraviolet radiation. For example, quercetin, a flavonoid identified in the leaf extracts of castor bean (*Ricinus communis* L.), exhibits lethal, ovicidal, and oviposition-deterrent effects on the beetle *Callosobruchus chinensis* L (376).

Anthocyanins, in contrast, are known to enhance disease resistance, as observed in certain grape cultivars that display increased tolerance to downy mildew. Moreover, specific root exudates, such as taxol released from potato and onions, can stimulate neighboring plants like tomato to attract beneficial *Bacillus* bacteria, thereby promoting overall plant health (377). The application of specific flavonoids in agriculture as growth regulators is well established. They are utilized to promote plant development and alleviate both abiotic and biotic stresses (378).

Mechanistic studies in *Arabidopsis thaliana* have further revealed that flavonols containing a C3–OH group regulate auxin transport by modulating P-glycoprotein activity and influencing key regulatory proteins, such as phosphatases and kinases (379).

## 11 New drug development and clinical synergy

Several commercially available pharmaceutical formulations now incorporate flavonoid compounds or standardized botanical flavonoid extracts (380). One example is Kang-En-Bei, an oral patch prescribed for mild recurrent oral ulcers, a condition traditionally attributed to excessive internal heat in the heart and spleen. This preparation consists of total flavonoids extracted from *Abelmoschus manihot* flowers and has demonstrated efficacy in relieving symptoms such as oral mucosal ulceration, localized erythema, swelling, and burning pain (380).

Recognized as the first novel Chinese medicine globally approved for the prevention and treatment of urolithiasis, Guangshitong^®^ is a total flavonoid capsule developed by Wuhan Jiulong Renfu Pharmaceutical Co., Ltd. Derived from the purified total flavonoids of *Desmodium styracifolium*, its therapeutic effects include clearing heat, dispelling dampness, promoting diuresis, and facilitating stone expulsion (132).

Additionally, a proprietary Chinese medicine originally derived from *Pueraria lobata* (Kudzu root) has been reformulated to standardize its total flavonoid content. Cardiovascular and cerebrovascular diseases, which involve hypertension, hyperlipidemia, migraine, coronary heart disease, myocardial infarction, angina pectoris, retinal vascular blockage, and sudden deafness, are the primary diseases treated by these preparations. Important components such as daidzin, methoxy puerarin, hydroxy puerarin, and puerarin are added to them (381).

Contemporary research continues to elucidate novel mechanisms of action for specific flavonoids. For instance, apigenin has been shown to mitigate *Mycoplasma pneumoniae* infection by activating the PPARγ signaling pathway. This activation elevates *Uhrf1* mRNA expression, enhances DNA methylation of the *TNF-*α promoter, and consequently suppresses *TNF-*α mRNA expression. The resulting decrease in *TNF-*α autocrine signaling helps prevent necroptotic cell death in alveolar macrophages. This finding uncovers a previously unrecognized protective mechanism against macrophage injury, underscoring the significant therapeutic potential of apigenin for *M. pneumoniae* infection (381).

Flavonoids frequently display beneficial synergistic effects, both in combination with other flavonoids and alongside conventional pharmaceutical agents. In colorectal cancer (CRC), numerous flavonoids exhibit both preventive and therapeutic activities. They can act synergistically with established anti-CRC treatments to promote apoptosis, inhibit carcinogenesis, and suppress tumor proliferation (382).

For instance, luteolin has been shown to act synergistically with lysosomal adenoviruses to selectively enhance tumor cell cytotoxicity and apoptosis. This agent selectively spares normal lung epithelial cells while enhancing the sensitivity of CRC cells to the chemotherapeutic drug oxaliplatin by modulating the Nrf2 signaling pathway. Moreover, the co-administration of EGCG or resveratrol with oxaliplatin or cisplatin has been reported to potentiate apoptotic effects and suppress tumor cell proliferation in CRC models (382).

Studies have shown that catechol-type flavonoids—specifically 7, 8-dihydroxyflavone, myricetin, and luteolin—disrupt bacterial iron homeostasis by reducing ferric iron (Fe^3+^) to its ferrous form (Fe^2+^). This mechanism produces a strong synergistic effect when these flavonoids are co-administered with the antibiotic colistin. Furthermore, these compounds enhance colistin's antibacterial efficacy by interfering with the bacterial two-component regulatory system pmrA/pmrB, leading to alterations in membrane charge that promote colistin binding and intensify membrane damage (383).

As a promising pharmacological agent, baicalin has shown activity against SARS-CoV-2 in various screenings. Additionally, a modified baicalin derivative is currently in phase II clinical trials targeting influenza treatment in adults, reflecting its potential therapeutic application in viral infections, suggesting considerable potential for broader antiviral drug development (384). Finally, the combination of puerarin and naloxone has been shown to improve recovery outcomes in patients with traumatic cerebral infarction (385).

## 12 Limitations

The limitations in research on flavonoids' biological activity, nutritional and immunological properties, and benefits to human health are summarized below.

### 12.1 Intrinsic physicochemical limitations

The intrinsic physiochemical limitations include planar structure, poor water and lipid solubility, low chemical stability, very low bioavailability, and inefficient absorption.

### 12.2 Limitations of conventional delivery systems

(i) Non-covalent interactions with carriers are weak and easily disrupted in acidic, high-ionic gastrointestinal conditions, causing premature release and poor systemic stability; (ii) many traditional carriers lack acid resistance and are unstable in the gastrointestinal tract; and (iii) short intestinal transit time makes sufficient, site-specific intestinal delivery difficult.

### 12.3 Pathophysiological constraints at the target site

High ROS levels in the inflamed intestine can oxidize quercetin to inactive quinones, thereby inactivating it exactly where it is needed.

### 12.4 Safety and clinical-use limitations

(i) Difficult, imprecise quantification of flavonoids in complex preparations, and (ii) risk of adverse reactions (from mild allergy/fever to anaphylactic shock and rare fatalities), likely linked to very stable, hard-to-separate charge-transfer complexes with serum lipoproteins.

### 12.5 Sourcing and manufacturing bottlenecks

(i) Plant extraction is limited by slow growth, low natural abundance, and environmental variability; (ii) total chemical synthesis is complex, multistep, low-yield, costly, and environmentally unfriendly; and (iii) microbial biosynthesis is promising, but current titers, yields, and productivities are still too low for economic industrial production.

## 13 Future prospects

Current research into the pharmacological properties of flavonoids is advancing rapidly, with the mechanisms of action and therapeutic potential of numerous compounds becoming increasingly well understood. These emerging insights are expected to catalyze major developments in the field. Future investigations are likely to focus on designing innovative drug delivery systems to enhance stability, bioavailability, and targeted therapeutic efficacy.

Additionally, the comprehensive functional elucidation of pivotal genes governing the flavonoid biosynthetic pathway will support metabolic engineering strategies to enhance biosynthetic efficiency and maximize production yields. It will also aid in structurally modifying flavonoid molecules to fine-tune their pharmacokinetic and pharmacodynamic profiles. A cornerstone of modern drug development lies in the chemical modification of flavonoid structures, a strategy aimed at optimizing their biological activity, improving solubility and metabolic stability, and minimizing potential toxicity (386).

The targeted incorporation of distinct functional groups onto the A and B rings, along with the synthesis or structural modification of the C ring, represents a vital aspect of this process. These structural engineering approaches are essential for creating novel flavonoid derivatives with enhanced biological activities and represent a central focus within contemporary flavonoid chemistry. Furthermore, elucidating the complex mechanisms of flavonoid biosynthesis and advancing cellular synthesis technologies are of paramount importance for addressing the persistent challenge of their limited natural availability, thereby paving the way for scalable and sustainable production (151).

Advances in metabolomics and genomics are poised to accelerate this progress by enabling the more efficient identification of gene clusters responsible for flavonoid biosynthesis. Through protein-directed evolution, metabolic engineering, and gene editing, key genetic components can be modularized into standardized “production parts,” facilitating the rational and efficient design of optimized metabolic pathways.

Future breakthroughs in this area will depend on streamlining chassis cell genomes and minimizing non-target metabolic fluxes, thereby improving overall biosynthetic efficiency. At the same time, the antimicrobial properties of flavonoids have emerged as a major area of research in recent decades, underscoring their potential as natural alternatives to conventional antimicrobial agents (387).

However, the clinical development of oral flavonoid-based therapeutics faces considerable obstacles, primarily due to their poor water solubility, rapid degradation, and extensive first-pass metabolism. These inherent limitations have prompted the development of innovative nano-formulations and advanced delivery systems to improve both solubility and stability. Among these strategies, encapsulation within nano-carriers has proven particularly effective, markedly enhancing the pharmacokinetic properties and safety profiles of flavonoids.

For example, topical delivery, when achieved through integration into nanomedicine formulations, enables more efficient penetration of the skin barrier while simultaneously reducing systemic side effects. Overall, the utilization of nanoparticles and related nano-formulations offers significant advantages, including high encapsulation efficiency, low toxicity, enhanced physicochemical stability, and precise targeted delivery to specific biological sites (388).

Consequently, the continuous innovation of advanced delivery platforms will undoubtedly persist as a central and critical goal in future flavonoid research (388).

## 14 Conclusions

Flavonoids possess wide-ranging bioactivities, yet their poor and variable bioavailability, driven by low solubility, extensive first-pass and microbial metabolism, and inter-individual differences in gut microbiota, remains a primary barrier to consistent clinical efficacy. SAR data remains largely empirical, focusing on simple correlations rather than quantitative, predictive models that integrate structure, pharmacokinetics, and metabolism, even though metabolites are often the true bioactive species *in vivo*.

Methodological heterogeneity across the *in vitro* and animal studies, including differences in doses, preparations, endpoints, and often non-standardized or impure extracts, limits comparability and weakens the evidence base for rational design and dosing in humans. Clinically, there is a striking imbalance between massive preclinical work and relatively few, small, or low-quality human trials, with major gaps in long-term safety, optimal dose ranges, and effects at both low and high exposures.

Pharmacokinetic, drug–drug interaction, and toxicity profiles (including organ-specific and endocrine effects) are insufficiently characterized for most compounds, hampering regulatory acceptance and precision use. On the production side, plant extraction, chemical synthesis, and current biosynthetic platforms all face challenges with scalability, cost, or process-efficiency, slowing access to standardized, reproducible flavonoid preparations for rigorous clinical testing.

Collectively, these limitations underscore the need for standardized extracts, advanced delivery systems, integrated SAR–metabolism models, and well-designed clinical trials to translate flavonoids into dependable therapeutics.
